# Synthesis and biological evaluation of pyridylpiperazine hybrid derivatives as urease inhibitors

**DOI:** 10.3389/fchem.2024.1371377

**Published:** 2024-03-13

**Authors:** Muhammad Akash, Sumera Zaib, Matloob Ahmad, Sadia Sultan, Sami A. Al-Hussain

**Affiliations:** ^1^ Department of Chemistry, Government College University Faisalabad, Faisalabad, Pakistan; ^2^ Department of Basic and Applied Chemistry, Faculty of Science and Technology, University of Central Punjab, Lahore, Pakistan; ^3^ Faculty of Pharmacy, Universiti Teknologi MARA, Puncak Alam, Selangor Darul Ehsan, Malaysia; ^4^ Atta-ur-Rahman Institute for Natural Products Discovery (AuRIns), Universiti Teknologi MARA, Puncak Alam, Selangor Darul Ehsan, Malaysia; ^5^ Department of Chemistry, College of Science, Imam Mohammad Ibn Saud Islamic University (IMSIU), Riyadh, Saudi Arabia

**Keywords:** pyridylpiperazine hybrid derivatives, heterocyclic compounds, urease inhibition, molecular docking, ADMET studies

## Abstract

Urease, a nickel-dependent enzyme found in various life forms, catalyzes urea breakdown, concluding nitrogen metabolism by generating ammonia and carbamate. This process causes a rise in pH, supports the survival of pathogens, and can lead to infections such as gastric disorders like ulcers and cancer in humans. *Helicobacter pylori* employs urease for survival in the acidic environment of the stomach and in protein synthesis. To treat such infections and inhibit the growth of pathogens, it is mandatory to obstruct urease activity; therefore, derivatives of 1-(3-nitropyridin-2-yl)piperazine were synthesized (**5a-o**; **7a-k**). All these newly synthesized compounds were investigated for urease inhibition by *in vitro* inhibition assays. The results showed that **5b** and **7e** are the most active inhibitors, having IC_50_ values of 2.0 ± 0.73 and 2.24 ± 1.63 µM, respectively. These IC_50_ values are lower than the IC_50_ value of the standard thiourea, which was 23.2 ± 11.0 µM. The hemolysis potential of **5b, 5c, 5i, 7e,** and **7h** was also determined; **7e** and **7h** exhibited good biocompatibility in human blood cells. Through *in silico* analysis, it was shown that both these potent inhibitors develop favorable interactions with the active site of urease, having binding energies of −8.0 (**5b**) and −8.1 (**7e**) kcal/mol. The binding energy of thiourea was −2.8 kcal/mol. Moreover, **5b and 7e** have high gastrointestinal permeability as predicted via computational analysis. On the other hand, the IC_50_ value and binding energy of precursor compound **3** was 3.90 ± 1.91 µM and −6.1 kcal/mol, respectively. Consequently, **5b** and **7e** can serve as important inhibitors of urease.

## 1 Introduction

Urease (urea amidohydrolase E.C. 3.5.1.5) is a nickel-dependent enzyme prevalent across diverse life forms, encompassing animals, plants, fungi, and bacteria (Mazzei et al., 2020). It facilitates the breakdown of urea into ammonia and carbamate, which is a biochemical process marking the end stage of nitrogen metabolism (Naseer et al., 2022; Lin et al., 2012). It results in the alkylation effect (rise in pH); hence, it supports the survival of numerous pathogenic microorganisms in the host body (Konieczna et al., 2012; Maier and Benoit, 2019; Ahmad et al., 2023). In humans, gastrointestinal infections are mostly caused by the alkaline pH, which leads to significant complications such as gastric ulcers and gastric cancer (Sohrabi et al., 2022). Pathogen-mediated gastric disorders are mainly attributed to *Helicobacter pylori*, which is a Gram-negative bacterium (Sukri et al., 2023). This bacterium utilizes urease activity not only to survive in the acidic environment of the stomach but also to synthesize proteins (Sohrabi et al., 2022). Owing to this significance, urease represents nearly 10% of the total content of proteins (Mazzei et al., 2020). In addition to *H. pylori*, urease activity is also crucial for the survival of *Staphylococcus aureus* (Zhou et al., 2019)*, Mycobacterium tuberculosis, Yersinia enterocolitica* (Righetto et al., 2020), and *Cryptococcus neoformans*, which cause kidney diseases (rodents), tuberculosis (humans), yersiniosis (humans) (Bhagat and Virdi, 2009), and cryptococcosis (humans) (Cox et al., 2000), respectively. Therefore, urease is a significant target to limit the survival of various pathogenic organisms. However, in this research paper, our core focus is to discover potential urease inhibitors that will help limit the survival of these pathogens.

The structure of urease is made up of four domains: the *N*-terminal *αβ* domain; *αβ* domain; *β* domain; and (*αβ*)8 TIM barrel domain, which consists of an active site and flap region. The active site encompasses two nickel atoms that are spaced apart by a distance of approximately 4 Å (Balasubramanian and Ponnuraj, 2010). One nickel interacts with a terminal water molecule and two histidine residues; the second nickel atom interacts with aspartate, two histidine atoms, and a solvent molecule. In addition, the active site binds with the substrate and initiates catalysis due to the presence of two histidine residues in it (Carter et al., 2009). Several inhibitors have been discovered such as sulphamethazine, sulphamethoxazole (Hamad et al., 2020), hydroxamic acids (Liu et al., 2018), benzimidazole (Zaman et al., 2019), *bis*-indole, and quinazoline-4(3*H*)-one (Uddin et al., 2020). Nevertheless, heterocycles with nitrogen atoms like pyridine and piperazine are very interesting due to their increased pharmacological activities (Moghadam et al., 2022; Naseer et al., 2022). Apart from pharmaceutical roles, inhibition of urease in agriculture is also essential to limit ammonia volatilization in the environment. Urease inhibitors play a mandatory role in prolonging the urea hydrolysis process, diminishing nitrogen (N) losses, and enhancing bioavailability (Matczuk and Siczek, 2021).

**FIGURE 1:**
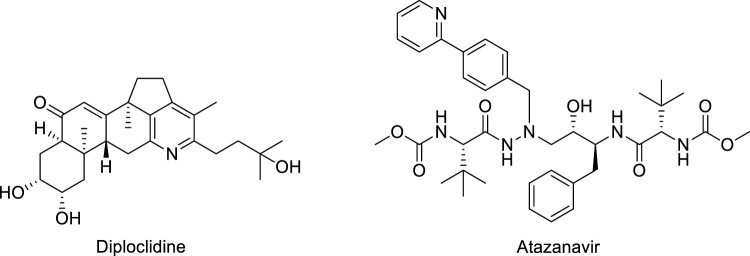
Structures of pyridine-based drugs.

**FIGURE 2:**
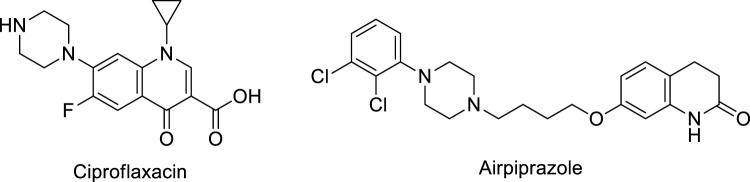
Structures of piperazine-based drugs.

**FIGURE 3:**
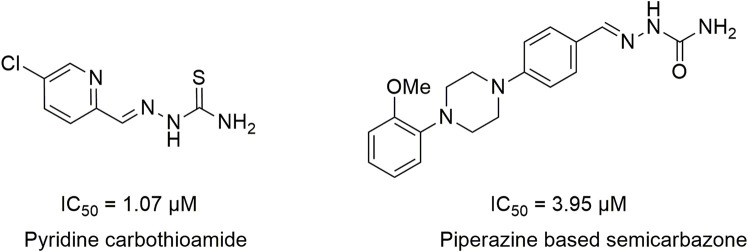
Structures of pyridine carbothioamides and piperazine-based semicarbazones as potent urease inhibitors.

Literature studies revealed that pyrido-*N*-substituted piperazines are key scaffolds for various enzymatic inhibitions (Swanson et al., 2005; Kumar et al., 2014). Pyridine is a backbone in the skeleton of marketed drugs (Jayasinghe et al., 2003; Chaves et al., 2021) (Figure 1). Piperazine is also a key ingredient for various biologically active scaffolds. They are core fragments of a variety of drugs (Campoli-Richards et al., 1988; Croxtall, 2012) (Figure 2). In recent literature, pyridine carbothioamide (Naseer et al., 2022) and piperazine-based semicarbazone (Moghadam et al., 2022) were found to be effective urease inhibitors (Figure 3). The coupling of two or more pharmacologically important scaffolds is a diverse approach that can produce hybrid molecules with enhanced biological activity. In continuation of our previous research work dealing with the development of various enzyme inhibitors, for example, α-amylase and α-glucosidase inhibitors (Khan et al., 2021; Khan et al., 2022; Saddique et al., 2022), we herein report a novel series of pyridine-based piperazines as potent urease inhibitors.

## 2 Results and discussion

### 2.1 Chemistry

**SCHEME 1:**
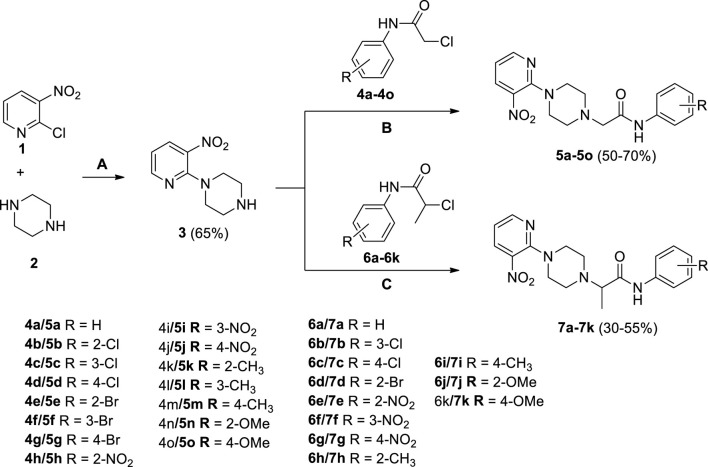
Synthesis of 2-(4-(3-nitropyridin-2-yl)piperazin-1-yl)-*N*-arylacetamide derivatives **5a-5o** and *N*-aryl-2-(4-(3-nitropyridin-2-yl)piperazin-1-yl)propanamide derivatives **7a-7k**. Reagents and conditions: **(A)** acetonitrile, reflux, 12 h **(B)** K_2_CO_3_, acetonitrile, reflux, 18–36 h **(C)** K_2_CO_3_, acetonitrile, reflux, 24–48 h.

Several 2-(4-(3-nitropyridin-2-yl)piperazin-1-yl)-*N*-arylacetamide derivatives **5a-5o** and *N*-aryl-2-(4-(3-nitropyridin-2-yl)piperazin-1-yl)propanamide derivatives **7a-7k** were synthesized, as shown in Scheme 1. The reaction of 2-chloro-3-nitropyridine (**1**) with excess piperazine (**2**) under reflux for 12 h in acetonitrile produced pyridinylpiperazine (**3**) in 65% yield. The presence of a nitro group at the 3-position withdraws the electron at the 2-position, making it a strong electrophilic center that facilitates the attack of the nucleophilic nitrogen atom of piperazine under nucleophilic aromatic substitution reaction. The reaction of piperazine (**3**) and 2-chloro-*N*-arylacetamides **4a-4o** in the presence of potassium carbonate in acetonitrile under reflux for 18–36 h afforded compounds **5a-5o** in 50%–70% yield. Similarly, piperazine (**3**) and 2-chloro-*N*-arylpropanamides **6a-6k** reacted in the presence of potassium carbonate in acetonitrile under reflux for 24–48 h furnished compounds **7a-7k** in 30%–55% yield. The compounds **4a-4o** and **6a-6k** were accessible from already reported methodologies (Cormier et al., 2012; Lavorato et al., 2017; Faiz et al., 2019; Abdel-Latif et al., 2020). All the synthesized compounds, **3**, **5a-5o,** and **7a-7k**, were purified by column chromatography and structures were established using spectroscopic methodologies. The HRMS justifies the chemical formula of each compound in terms of [M]^+^, [M+H]^+^, and [M+2H]^+2^ peaks. The nitrogenous heterocyclic compounds having basic nitrogen (nitrogen with available lone-pair) may protonate, causing peaks greater than [M+H]^+^ like [M+2H]^+^ (Joyce and Richards, 2011). The ^1^HNMR spectra of **3**, **5a-5o,** and **7a-7k** revealed the presence of methylene as well as all aromatic protons. Similarly, ^13^CNMR spectra of **3**, **5a-5o,** and **7a-7k** revealed the presence of C=O in all compounds. The FT-IR spectra of **3**, **5a-5o**, and **7a-7k** include the peaks of functionalities like NH (3,343–3,185 cm^−1^), C=O (1,698–1,664), NO_2_ (1,597–1,586 cm^−1^), and CH_2_ (1,513–1,481 cm^−1^), which is comparable with literature (Krishnakumar and Muthunatesan, 2006; George et al., 2018; Crocker et al., 2022).

### 2.2 Evaluation of structure-activity relationship

The inhibitory potential of piperazine **(3)**, 1-(3-nitropyridin-2-yl)piperazine derivatives (**5a-o and 7a-k**) and standard (thiourea) against jack bean urease was determined by the indophenol method, as shown in Table 1 **.** The thiourea was selected as a positive standard because it has been well-characterized and well-documented as a standard inhibitor of urease in literature. According to the results elucidated in Table 1, all the compounds showed good inhibitory activities against urease, with their IC_50_ values ranging between 2.0 ± 0.73 μM and 14.12 ± 0.67 μM. Moreover, piperazine **(3)** and thiourea have IC_50_ values of 3.90 ± 1.91 and 23.2 ± 11.0 μM, respectively.

**TABLE 1:** The IC_50_ values of all the compounds have been elucidated in tabular form.

Nucleus	Compound Codes	Substituents (R)	IC_50_ ± SEM (µM)
	3		3.90 ± 1.91
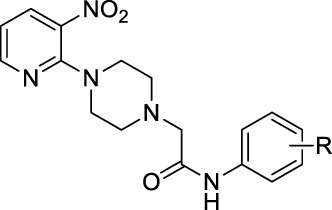	5a	H	3.58 ± 0.84
	5b	2-Cl	2.0 ± 0.73
	5c	3-Cl	2.13 ± 0.82
	5d	4-Cl	8.25 ± 0.41
	5e	2-Br	4.47 ± 0.44
	5f	3-Br	5.24 ± 0.45
	5g	4-Br	8.43 ± 0.41
	5h	2-NO_2_	7.12 ± 0.39
	5i	3-NO_2_	2.56 ± 0.55
	5j	4-NO_2_	4.19 ± 0.41
	5k	2-Me	14.12 ± 0.67
	5l	3-Me	7.14 ± 0.46
	5m	4-Me	9.37 ± 0.40
	5n	2-OMe	5.33 ± 0.44
	5o	4-OMe	5.21 ± 0.47
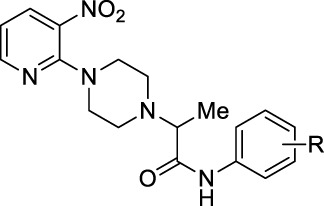	7a	H	7.41 ± 0.44
	7b	3-Cl	6.70 ± 0.37
	7c	4-Cl	7.38 ± 0.37
	7d	2-Br	6.74 ± 0.49
	7e	2-NO_2_	2.24 ± 1.63
	7f	3-NO_2_	7.66 ± 0.37
	7g	4-NO_2_	6.13 ± 0.40
	7h	2-Me	3.01 ± 0.45
	7i	4-Me	5.65 ± 0.40
	7j	2-OMe	5.32 ± 0.41
	7k	4-OMe	5.95 ± 0.43
Thiourea (standard)			23.2 ± 11.0

1-(3-Nitropyridin-2-yl)piperazine is the static motif in all the test compounds, which were further diversified by the attachment of *N*-phenylpropionamide and *N*-phenylisobutyramide. This diversification resulted in two sets of compounds: the first set of compounds (**5a-o**) contain 2-(4-(3-nitropyridin-2-yl)piperazin-1-yl)-*N*-arylacetamide nucleus (nucleus A) while the second set of compounds (**7a-k**) contain N-aryl-2-(4-(3-nitropyridin-2-yl)piperazin-1-yl)propanamide nucleus (nucleus B). **5b** and **5c** in the first set are potent inhibitors because they have the lowest and comparable IC_50_ values, which are 2.13 ± 0.82 µM and 2.0 ± 0.73 µM, respectively. This ideal activity of **5b** is attributed to the presence of an electron-withdrawing group (EWG) chlorine (Cl) at *ortho*-position (*ortho-*position) *of* the aryl group of *N*-phenylpropionamide. The urease inhibitory activity has a tendency to decrease (IC_50_ = 4.47 ± 0.44 µM) when Cl is substituted with another halogen bromine (Br) (**5e**). This decrease in activity is because of the decrease in electronegativity and the increase in size of Br. In addition, attachment of another electrophilic group, NO_2_ (**5h**), causes a decrease in the urease inhibitory activity (IC_50_ = 7.12 ± 0.39 µM). Similarly, the presence of methyl (**5k**) and methoxy (**5n**) also resulted in the decreased inhibitory potential of nucleus A against urease due to their electron-donating nature.

The aryl group of nucleus A was also substituted at meta-position with electron-donating groups (EDGs) and EWGs. In the absence of any substitution at the aryl group (**5a**), the inhibitory activity of nucleus A against urease was observed to be 3.58 ± 0.84 µM. The urease inhibitory potential of **5c** and **5i** were comparable due to the presence of electrophilic groups, namely, Cl (IC_50_ = 2.13 ± 0.82 µM) and NO_2_ (IC_50_ = 2.56 ± 0.55 µM) at meta-positions, accordingly. Contrarily, a decrease in inhibitory potential, with an IC_50_ value of 5.24 ± 0.45 µM, was observed when Br was substituted at the meta-position of the aryl group (**5f)**. The substitution of EDG such as methyl at the meta-position of aryl group (**5l)** resulted in the inactivation of nucleus A (IC_50_ = 7.14 ± 0.46 µM).

The substitution of EDGs and EWGs at *para*-position of the aryl group of nucleus A did not result in potent inhibitors of the urease enzyme. **5d** with Cl substitution had an IC_50_ value of 8.25 ± 0.41 µM, which increased by replacing Cl with another EWG, namely, NO_2_ (IC_50_ = 4.19 ± 0.41 µM) (**5j**). Contrarily, substituting Cl with Br (**5g**) did not significantly alter the inhibitory potential of nucleus A (IC_50_ = 8.43 ± 0.41 µM). The presence of methyl group (**5m)** inactivated nucleus A (IC_50_ = 9.37 ± 0.40 µM); on the other hand, the substitution of methoxy at *para-*position (**5o)** increased the urease inhibitory potential of nucleus A (IC_50_ = 5.21 ± 0.47 µM).

In the case of nucleus B, the substitution of the aryl group at ortho-position increased the urease inhibitory potential of **7a**, **7d**, **7e**, **7h**, and **7j**. An amplification tendency in the inhibitory activity of nucleus B (IC_50_ = 6.74 ± 0.49 µM) was observed upon substitution with an EWG, namely, Br at ortho-position (**7d**) as compared to **7a** (IC_50_ = 7.41 ± 0.44 µM). The addition of ortho-NO_2_ (**7e**) or ortho-methyl (**7h**) resulted in a increased inhibitory activity of nucleus B against urease [IC_50_ = 4.19 ± 0.41 µM (**7e)**; IC_50_ = 3.01 ± 0.45 µM (**7h**)]. Similarly, the presence of methoxy substituent at ortho-position (**7j**), also enhanced the inhibition of nucleus B (IC_50_ = 5.32 ± 0.41 µM).

**FIGURE 4:**
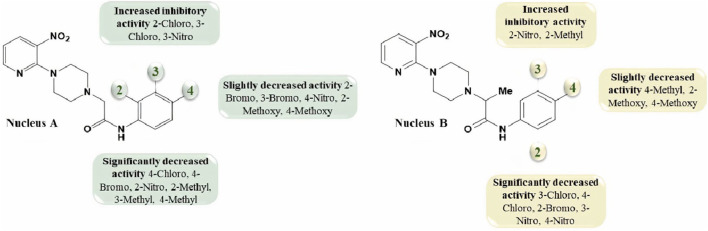
The variations in the urease inhibitory activity of nucleus A and B due to different substituents.

Compounds **7c, 7g, 7i, and 7k** are para-substituted variants of nucleus B, and their IC_50_ ranged between 5.65 and 7.38 µM. The urease inhibitory potential of EWGs Cl (**7c**) and NO_2_ (**7g)** were 7.38 ± 0.37 µM and 6.13 ± 0.40 µM, respectively. The inhibitory activity of nuclease B was further increased when methyl (**7i)** and methoxy (**7k**) substituents were introduced at the ortho-position of the aryl group. The IC_50_ value of **7i** was 5.65 ± 0.40 µM, while the IC_50_ value of **7k** was 5.95 ± 0.43 µM. Only two meta-substituted variants at the aryl group of nucleus B were obtained; it is quite interesting to know that both these substituents are EWGs: Cl-substituted **7b** (IC_50_ = 6.70 ± 0.37 µM) and NO_2_-substituted **7f** (IC_50_ = 7.66 ± 0.37 µM) (Figure 4).

### 2.3 Hemolysis potential

**FIGURE 5:**
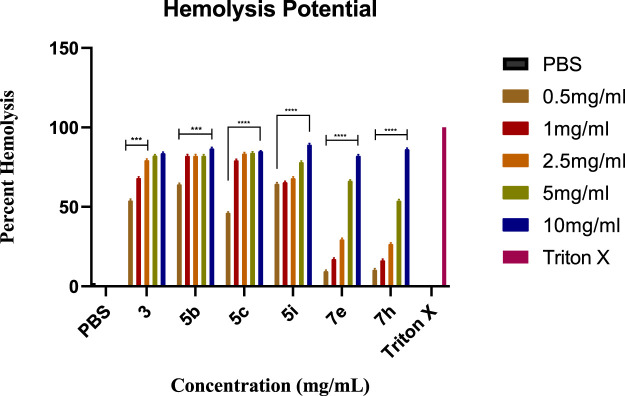
The graph represents the comparison of hemolysis activity of Triton X-100, PBS, **3**, **5b**, **5c**, **5i**, **7e**, and **7h** at different concentrations.

1-(3-nitropyridin-2-yl)piperazine **3** and its derivatives (**5b**, **5c**, **5i**; **7e**, **7h**) were analyzed for hemolysis activity due to their remarkable urease inhibition potential. Results revealed that, out of all screened compounds, **7e** and **7h** exhibited good biocompatibility in human blood cells depending upon hemolysis percentage at 0.5 mg/mL and 1 mg/mL nearer to that of PBS. These results showed that compounds **7e** and **7h** are favorable for injection at low concentrations due to good biocompatibility and the results were found statistically significant after the application of one-way ANOVA as the value of p was found to be less than 0.05. Experimental data on hemolysis percentage have been depicted in Figure 5.

### 2.4 ADMET investigation

admetSAR, ProTox-II, and eMolTox were used for the *in silico* pre-clinical evaluation of the precursor compound **3** and the most potent compounds, which were selected based on the lowest IC_50_. **5b** was chosen from the first set of compounds while **7e** was scrutinized from the second set of compounds to investigate their druggable properties. The ADMET evaluation of compound **3** predicted that it follows Lipinski’s rule of five, having a molecular weight of 208.096 g/mol, five hydrogen bond acceptors, one hydrogen bond donor, two rotatable bonds, and a topological polar surface area (TPSA) of 71.3 Å^2^. Moreover, it has a probability of 0.9693 for crossing the blood-brain barrier(BBB). Compound **3** is also a non-inhibitor and non-substrate of cytochrome P450 (CYP) enzymes in the hepatic tissue, except for CYP450 1A2, as shown in Table 2. The toxic substructure prediction interpreted that compound **3** may produce toxicophores (Supplementary Table S1). Moreover, acute rat toxicity can be reduced *in vivo* trials by administering the dose via oral route because this route has the highest LD_50_ value of 332.2 mg/kg and belongs to predicted toxicity class 4 (Supplementary Table S2).

**TABLE 2:** The pre-clinical investigation of **3**, **5b**, and **7e**.

Model	Compound 3		5b		7e	
	Result	Probability	Result	Probability	Result	Probability
Absorption						
Blood-Brain Barrier	BBB+	0.9693	BBB+	0.9202	BBB+	0.8223
Human Intestinal Absorption	HIA+	0.9947	HIA+	0.9841	HIA+	0.9744
Caco-2 Permeability	Caco2-	0.5181	Caco2-	0.5856	Caco2-	0.5655
P-glycoprotein Substrate	Substrate	0.6357	Substrate	0.6785	Substrate	0.7732
P-glycoprotein Inhibitor	Non-Inhibitor	0.8143	Inhibitor	0.5310	Non-inhibitor	0.6557
	Non-Inhibitor	0.8079	Non-Inhibitor	0.6847	Non-inhibitor	0.8700
Distribution						
Subcellular localization	Mitochondria	0.7632	Mitochondria	0.8867	Mitochondria	0.8363
Metabolism						
CYP450 2C9 Substrate	Non-substrate	0.8282	Non-substrate	0.8245	Non-substrate	0.7524
CYP450 2D6 Substrate	Non-substrate	0.7450	Non-substrate	0.8024	Non-substrate	0.7968
CYP450 3A4 Substrate	Non-substrate	0.6142	Substrate	0.5694	Substrate	0.5510
CYP450 1A2 Inhibitor	Inhibitor	0.9264	Non-inhibitor	0.5585	Non-inhibitor	0.8725
CYP450 2C9 Inhibitor	Non-inhibitor	0.5542	Non-inhibitor	0.6006	Inhibitor	0.6430
CYP450 2D6 Inhibitor	Non-inhibitor	0.9465	Non-inhibitor	0.8002	Non-inhibitor	0.9124
CYP450 2C19 Inhibitor	Non-inhibitor	0.5000	Inhibitor	0.7483	Non-inhibitor	0.6819
CYP450 3A4 Inhibitor	Non-inhibitor	0.8685	Inhibitor	0.5960	Non-inhibitor	0.6327
CYP Inhibitory Promiscuity	High CYP Inhibitory Promiscuity	0.6539	High CYP Inhibitory Promiscuity	0.9537	High CYP Inhibitory Promiscuity	0.7548
Toxicity						
Human Ether-a-go-go-Related Gene Inhibition	Strong inhibitor	0.8507	Strong inhibitor	0.6399	Weak inhibitor	0.6133
	Inhibitor	0.6789	Inhibitor	0.7389	Inhibitor	0.6545
AMES Toxicity	AMES toxic	0.7449	AMES toxic	0.6624	AMES toxic	0.7466
Carcinogens	Non-carcinogens	0.8023	Non-carcinogens	0.7178	Non-carcinogens	0.7897
Fish Toxicity	Low FHMT	0.6501	High FHMT	0.6395	High FHMT	0.5597
*Tetrahymena* Pyriformis Toxicity	High TPT	0.9334	High TPT	0.9880	High TPT	0.9616
Honey Bee Toxicity	Low HBT	0.8436	Low HBT	0.9218	Low HBT	0.9102
Biodegradation	Not readily biodegradable	0.9109	Not readily biodegradable	0.9729	Not readily biodegradable	0.7090
Carcinogenicity (Three-class)	Danger	0.3640	Non-required	0.3975	III	0.6596

The results obtained via eMolTox revealed that **5b** has a molecular weight of 375.11 g/mol, five rotatable bonds, six hydrogen bond acceptors, and one hydrogen bond donor. Moreover, the probability of **5b** penetrating the brain is 0.9202, and easily crosses the gastrointestinal lining with a probability of 0.9841, as shown in Table 2. Its topological polar surface area is 91.61 Å^2^ and LogP is 2.404. In addition, it may be toxic to the nervous system, being a modulator of P2X purinoceptor 7, and can also produce toxicophores (substructures) (Supplementary Table S1). Furthermore, GUSAR analysis predicted that **5b** can be administered to rats via the subcutaneous route because this route has the highest LD_50_ (1,360 mg/kg) with a predicted toxicity class 5 (Supplementary Table S2).

Pre-clinical analysis of the most potent inhibitor of the second set of compounds predicted that **7e** has six rotatable bonds, 29 heavy atoms, 12 aromatic heavy atoms, 134.75 Å^2^ TPSA, and 2.047 LogP. It belongs to the soluble class of compounds and has high gastrointestinal absorption with a probability of 0.9744. In addition, the blood-brain barrier penetration probability is 0.8223 and it is a non-carcinogenic and non-inhibitor of renal organic cation transporter and P-glycoprotein (P-gp), as represented in Table 2. Apart from the ADMET analysis, the toxicophores were also predicted via eMolTox, which includes substructures having the ability to covalently bind with protein and DNA. Moreover, toxic substructures may also be produced (Supplementary Table S1). Acute rat toxicity was predicted via GUSAR, which showed that oral administration of **7e** to rats will be non-toxic because of the highest LD_50_ value (911.1 mg/kg) (Supplementary Table S2).

Although compounds **5b** and **7e** are the lead inhibitors of urease in this research, their gastrointestinal absorption determined by *in silico* analysis needs to be limited. This can be reduced by selecting the best drug delivery system, such as nanoparticles (Gupta et al., 2023).

### 2.5 Molecular docking studies

The molecular docking of the most potent inhibitors of both sets of compounds was performed by AutoDockTools 1.5.7 and SeeSAR version 12.1.0. The most druggable binding site of the urease (PDB id: 4H9M) was identified via the binding site mode of SeeSAR and has a DoGSiteScore of 0.57. After docking by AutoDockTools 1.5.7, nine poses for each of the potent inhibitors were generated and the best poses with the lowest binding energy were selected for the visualization of intermolecular interactions. The binding energy of the best pose of precursor compound **3**, **5b**, **7e**, and **thiourea** was −6.1, −8.0, −8.1, and −2.8 kcal/mol, respectively, as shown in Table 3.

**TABLE 3:** The binding energies of the best-docked poses of **5b** and **7e** with urease.

Potent inhibitors	Binding energies (kcal/mol)
**3**	−6.1
**5b**	−8.0
**7e**	−8.1
Thiourea	−2.8

**FIGURE 6:**
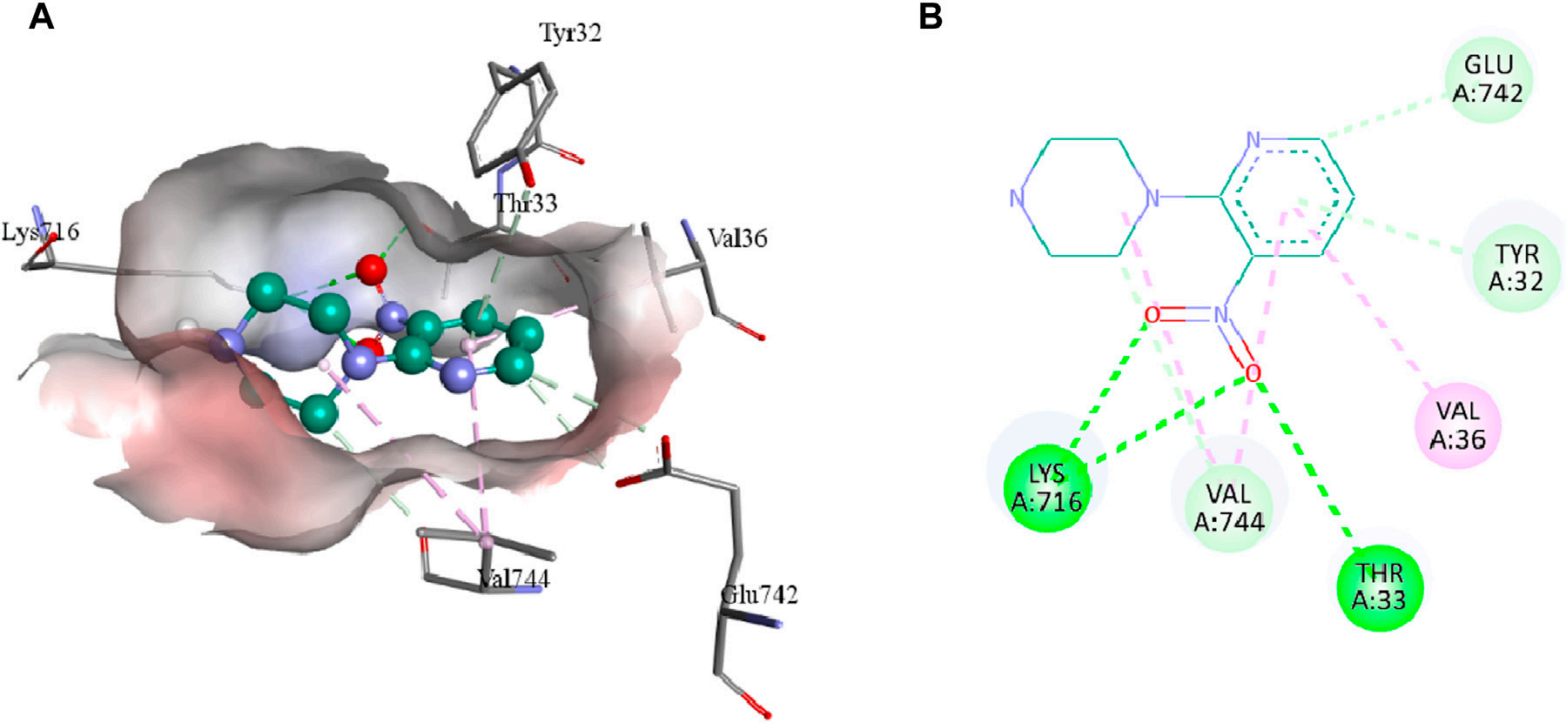
The 3D **(A)** and 2D **(B)** representation of molecular interactions of compound **3** with the binding site residues of urease. Compound **3** is represented by a green color while the dotted lines are showing the molecular interactions. Green: conventional hydrogen interaction; Light blue: carbon hydrogen interaction; Pink: hydrophobic interaction.

The intermolecular interactions of **3**, **5b,** and **7e** were visualized by BIOVIA Discovery Studio molecular visualizer 2021. According to the results, compound **3** develops hydrogen and hydrophobic interactions with the binding site residues of urease. Lys716 forms two conventional hydrogen interactions: one with O8 (2.99 Å) and the other with O9 (2.95 Å) of compound **3**. Similarly, Thr33 also forms conventional hydrogen interactions with the O8 (3.05 Å) of the ligand. Compound **3** also interacts with the Glu742 and Val744 by forming carbon hydrogen interactions. The pyridine ring of compound **3** forms a π-donor hydrogen and two π-alkyl interactions with Tyr32 (4.13 Å), Val36 (4.76 Å) and Val744 (4.81 Å) of urease. Additionally, Val744 also develops alkyl interaction with the piperazine ring (5.03 Å) of compound **3,** as represented in Figure 6.

**FIGURE 7:**
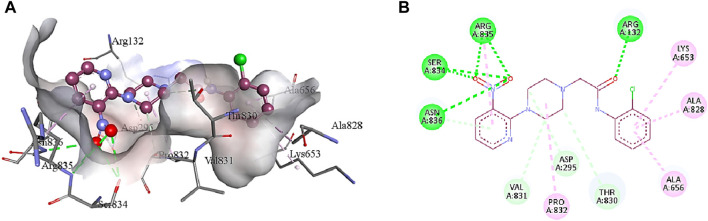
The interactions between the binding site of urease and **5b** can be visualized in 3D **(A)** and 2D **(B)** format. Dotted lines of various colors indicated these intermolecular interactions. Green represents conventional hydrogen bonds; light blue indicates carbon hydrogen bonds; pink highlights the alkyl and π-alkyl bonds.

**5b** interacts with the active site residues of urease by developing alkyl, π-alkyl, π-donor hydrogen interactions, carbon hydrogen interactions, and conventional hydrogen interactions (Table 4). Lys653, Ala656, and Ala828 interact by π-alkyl interactions (5.47 Å; 4.04 Å; 4.72 Å) with the aromatic ring of **5b**. Another π-alkyl interaction exists between Arg835 of the active pocket and the pyridine ring. The alkyl interaction is formed by Pro832 with the piperazine ring (5.01 Å) which also develops carbon hydrogen bonds with Asp295 (3.69 Å), Thr830 (3.69 Å), and Val831 (3.43 Å). In addition, the formation of conventional hydrogen interactions with the **5b** atoms is attributed to the presence of Arg132 (3.27 Å), Ser834 (3.21 Å), Arg835 (2.91 Å) and Asn836 (3.06 Å). Asn836 (3.50 Å) also develops a carbon hydrogen interaction with the pyridine ring of the ligand, as indicated in Figure 7.

**TABLE 4:** The detailed description of intermolecular interactions between urease residues and compound 3 and potent ligands **5b** and **7e** along with their bond lengths.

Potent compounds	Binding interactions			
	Ligand atom	Receptor residue	Interaction type	Distance (Å)
**3**	O8	Thr33	H-bond	3.05
	O8	Lys716	H-bond	2.99
	O9	Lys716	H-bond	2.95
	C2	Glu742	H-bond	3.65
	C2	Glu742	H-bond	3.47
	C16	Val744	H-bond	3.74
	Pyridine ring	Tyr32	π-donor H-bond	4.13
	Piperazine ring	Val744	Alkyl	5.03
	Pyridine ring	Val744	π-alkyl	4.81
	Pyridine ring	Val36	π-alkyl	4.76
**5b**	O20	Arg132	H-bond	3.27
	O9	Asn836	H-bond	3.06
	O9	Ser834	H-bond	3.21
	O9	Arg835	H-bond	2.91
	O8	Ser834	H-bond	2.91
	O8	Arg835	H-bond	3.32
	C14	Thr830	H-bond	3.69
	C15	Val831	H-bond	3.43
	C11	Asp295	H-bond	3.69
	Pyridine ring	Asn836	π-donor H-bond	3.50
	Piperazine ring	Pro832	Alkyl	5.01
	Aromatic ring	Lys653	π-alkyl	5.47
	Aromatic ring	Ala656	π-alkyl	4.04
	Aromatic ring	Ala828	π-alkyl	4.72
	Pyridine ring	Arg835	π-alkyl	4.98
**7e**	O8	Arg132	H-bond	3.23
	O9	Arg132	H-bond	3.00
	O9	Asn297	H-bond	3.03
	N3	Thr830	H-bond	2.89
	C2	Thr830	H-bond	3.34
	O29	Arg835	H-bond	3.27
	O29	Asn836	H-bond	3.02
	O19	Asn836	H-bond	2.81
	Piperazine ring	Asp295	H-bond	3.43
	Aromatic ring	Asn836	π-donor H-bond	4.01
	Pyridine ring	Asp652	π-anion	3.87
	Pyridine ring	Ala656	π-alkyl	4.79
	Aromatic ring	Leu71	π-alkyl	4.94
	Aromatic ring	Arg835	π-alkyl	4.59

**FIGURE 8:**
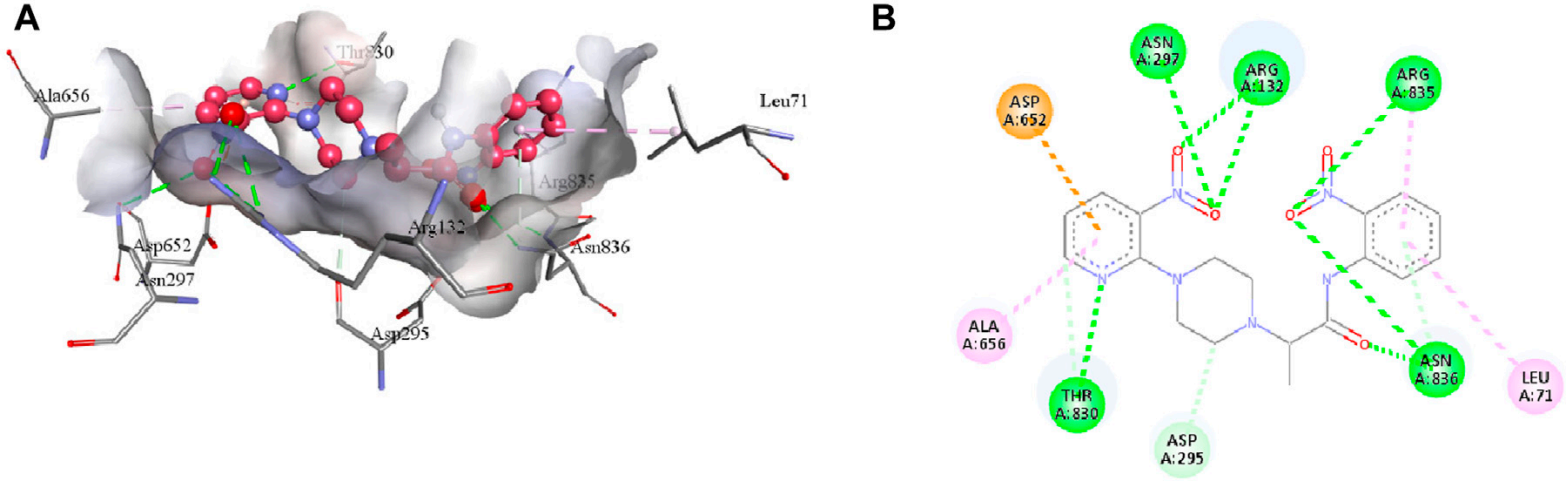
The 3D **(A)** and 2D **(B)** visualization of molecular interactions between **7e** and the active site residues of urease. These intermolecular interactions are indicated by dotted lines of varying colors. Green represents conventional hydrogen bonds; light blue indicates carbon hydrogen and π-donor hydrogen bonds; pink highlights the π-alkyl bonds; orange is an indicator of π-anion interaction.

Compound **7e** forms the conventional hydrogen, π-alkyl, π-anion, π-donor hydrogen, and carbon hydrogen interactions with the residues of the active pocket of urease (Table 4). Both the NO_2_ groups develop conventional hydrogen interactions with the Arg132 (3.23 Å), Asn297 (3.03 Å), Arg835 (3.27 Å), and Asn836 (3.02 Å); Asp652 forms π-anion interaction (3.87 Å) with the pyridine ring of **7e**. Another conventional hydrogen interaction is formed by the Thr830 with the nitrogen of the pyridine ring (2.89 Å), with which Thr830 also interacts by carbon hydrogen interaction (3.34 Å). The same pyridine ring of **7e** also develops π-alkyl interaction with the Ala656 (4.79 Å) of the urease active site. Additionally, Leu71 and Asn836 interact with the aromatic ring of **7e** via π-alkyl interactions. Lastly, Asn295 forms a carbon hydrogen interaction with the piperazine ring of the ligand (3.43 Å), as indicated in Figure 8.

## 3 Materials and methods

### 3.1 General

All the chemicals, reagents, and solvents were purchased from Alfa Aesar (Kandal, Germany) and were utilized without any further purification. The ^1^HNMR (400 MHz) and ^13^CNMR (100 MHz) were recorded in dimethyl sulfoxide (DMSO) Bruker DPX spectrophotometer (Bruker; Zürich, Switzerland). The chemical shifts were recorded in ppm reference to tetramethylsilane. The HRMS of all the synthesized compounds were recorded with LCMS/MS Q-TOF (Agilent Technologies 6520, Senta Clara, Ca, United States). Thin-layer chromatography (CHCl_3_/MeOH) was used in combination with a Spectroline E-Series UV lamp to monitor the progress of chemical reactions (Alfa Aesar, Kandal, Germany). Compounds **3**, **5a-5o,** and **7a-k** were produced according to Scheme 1.

### 3.2 Procedure for the synthesis of 1-(3-nitropyridin-2-yl)piperazine (3)

40.5 g (472 mmol) of piperazine (**2**) was dissolved in 100 mL of acetonitrile on stirring. A solution of 15 g (94.30 mmol) of 2-chloro-3-nitropyridine (**1**) in 50 mL of acetonitrile was added to it. The mixture was refluxed for 12 h on constant stirring. The reaction was monitored every hour with the help of TLC. The yellow solid (**3**) produced was extracted with chloroform and purified by column chromatography using silica (0.040–0.063 mm) in chloroform/methanol (90:10) with 65% (12.76 g) yield.

#### 3.2.1 1-(3-Nitropyridin-2-yl)piperazine (**3**)

Yield 65%; Brownish-yellow solid; m.p. 77°C–79°C. IR: ν (cm^−1^): 3,174 (NH), 1,590 (NO_2_), 1,556 (C=C), 1,435 (C=N), 1,230 (C-N). ^1^H NMR (400 MHz) δ (ppm): 8.29–8.30 (m, 1H, Ar-H), 8.15 (dd, 1H, J = 7.95 Hz, 1.8 Hz, Ar-H), 6.82-6.84 (m, 1H, Ar-H), 3.23–3.25 (m, 4H, piperazinyl), 2.71–2.73 (m, 4H, piperazinyl). ^13^C NMR (100 MHz) δ (ppm): 152.7, 152.5, 136.4, 132.8, 114.3, 48.7 (2C), 45.0(2C). HRMS: m/z [M+2H]^+2^ calculated: 210.1106; found: 210.2011.

### 3.3 Procedure for the synthesis of 2-(4-(3-nitropyridin-2-yl)piperazin-1-yl)-N-arylacetamide derivatives **5a-5o**

A solution of 2-chloro-N-arylacetamides **4a-4o** (0.15 mmol) was prepared in 10 mL of acetonitrile and added to the stirred mixture of 1-(3-nitropyridin-2-yl)piperazine (**3**) (0.15 mmol) and potassium carbonate (0.3 mmol) in 15 mL of acetonitrile. The reaction mixture was refluxed for 18–36 h on stirring by monitoring with TLC. On the addition of water, colored (yellow to red) precipitates of 2-(4-(3-nitropyridin-2-yl)piperazin-1-yl)-N-arylacetamide derivatives **5a-5o** were formed. The produced solid was collected and purified by column chromatography. The calculated yield was found to be 50%–70%.

#### 3.3.1 2-(4-(3-Nitropyridin-2-yl)piperazin-1-yl)-N-phenylacetamide (**5a**)

Yield 64%; Orange solid; m.p. 79°C–80°C. IR: ν (cm^−1^): 3,301 (NH), 1,677 (C=O), 1,589 (NO_2_), 1,552 (C=C), 1,500 (CH_2_), 1,436 (C=N), 1,237 (C-N). ^1^H NMR (400 MHz) δ (ppm): 9.86 (s, 1H, NH), 8.31–8.33 (m, 1H, Ar-H), 8.18 (dd, 1H, J= 8.1 Hz, 1.65 Hz, Ar-H), 7.52 (d, 2H, J= 7.9 Hz, Ar-H), 7.28 (d, 2H, J= 7.6 Hz, Ar-H), 7.06 (t, 1H, J= 7.35 Hz, Ar-H), 6.86–6.88 (m, 1H, Ar-H), 3.38 (Br s, 4H, piperazinyl), 3.19 (s, 2H, methylene), 2.60 (Br s, 4H, piperazinyl). HRMS: m/z [M+H]^+^ calculated: 342.1561; found: 342.1817.

#### 3.3.2 N-(2-Chlorophenyl)-2-(4-(3-nitropyridin-2-yl)piperazin-1-yl)acetamide (**5b**)

Yield 69%; Yellow solid; m.p. 108°C–109°C. IR: ν (cm^−1^): 3,248 (NH), 1,690 (C=O), 1,593 (NO_2_), 1,556 (C=C), 1,507 (CH_2_), 1,433 (C=N), 1,232 (C-N). ^1^H NMR (400 MHz) δ (ppm): 9.94 (s, 1H, NH), 8.41–8.42 (m, 1H, Ar-H), 8.25–8.27 (m, 1H, Ar-H), 8.21 (dd, 1H, J= 8.25 Hz, 1.6 Hz, Ar-H), 7.52 (dd, 1H, J= 8.05, 1.45 Hz), 7.34–7.37 (m, 1H, Ar-H), 7.13–7.17 (m, 1H, Ar-H), 6.92–6.94 (m, 1H, Ar-H), 3.45 (Br s, 4H, piperazinyl), 3.25 (s, 2H, methylene), 2.68 (Br s, 4H, piperazinyl). ^13^C NMR (δ): 168.4 (C=O), 152.0 (Ar-C), 151.9 (Ar-C), 135.8 (Ar-C), 134.1 (Ar-C), 132.7 (Ar-C), 129.2 (Ar-C), 127.8 (Ar-C), 125.2 (Ar-C), 123.3 (Ar-C), 121.8 (Ar-C), 114.2 (Ar-C), 60.9 [N(CH_2_)CO], 52.2 (piperazine, 2 x NCH_2_), 47.7 (piperazine, 2 x NCH_2_). ^1^HRMS: m/z [M+2H]^+^ calculated: 377.1244; found: 377.3016.

#### 3.3.3 N-(3-Chlorophenyl)-2-(4-(3-nitropyridin-2-yl)piperazin-1-yl)acetamide (**5c**)

Yield 55%; Yellow solid; m.p. 71°C–73°C. IR: ν (cm^−1^): 3,276 (NH), 1,664 (C=O), 1,593 (NO_2_), 1,552 (C=C), 1,491 (CH_2_), 1,429 (C=N), 1,236 (C-N). ^1^H NMR (400 MHz) δ (ppm): 9.97 (s, 1H, NH), 8.39–8.40 (m, 1H, Ar-H), 8.23 (dd, 1H, J = 8.15 Hz, 1.7 Hz, Ar-H), 7.85–7.86 (m, 1H, Ar-H), 7.55–7.57 (m, 1H, Ar-H), 7.33 (t, 1H, J = 8.15 Hz, Ar-H), 7.10–7.13 (m, 1H, Ar-H), 6.88–6.91 (m, 1H, Ar-H), 3.42 (Br s, 4H, piperazinyl), 3.20 (s, 2H, methylene), 2.61 (Br s, 4H, piperazinyl). ^13^C NMR (δ): 169.1 (C=O), 152.5 (Ar-C), 152.4 (Ar-C), 140.4 (Ar-C), 136.3 (Ar-C), 133.4 (Ar-C), 133.0 [ClC(CH)_2_], 130.8 (Ar-C), 123.6 (Ar-C), 119.6 (Ar-C), 118.5 (Ar-C), 114.3 (Ar-C), 61.8 [N(CH_2_)CO], 52.8 (piperazine, 2 × NCH_2_), 47.8 (piperazine, 2 × NCH_2_). HRMS: m/z [M+2H]^+^ calculated: 377.1244; found: 377.2965.

#### 3.3.4 N-(4-Chlorophenyl)-2-(4-(3-nitropyridin-2-yl)piperazin-1-yl)acetamide (**5d**)

Yield 61%; Yellow solid; m.p. 99°C–100°C. IR: ν (cm^−1^): 3,334 (NH), 1,685 (C=O), 1,595 (NO_2_), 1,554 (C=C), 1,487 (CH_2_), 1,433 (C=N), 1,239 (C-N). ^1^H NMR (400 MHz) δ (ppm): 9.95 (s, 1H, NH), 8.32–8.34 (m, 1H, Ar-H), 8.18 (dd, 1H, J= 8.1 Hz, 1.75 Hz), 7.56–7.59 (m, 2H, Ar-H), 7.30–7.32 (m, 2H, Ar-H), 6.85–6.88 (m, 1H, Ar-H), 3.38 (Br s, 4H, piperazinyl), 3.15 (s, 2H, methylene), 2.56 (Br s, 4H, piperazinyl). ^13^C NMR (δ): 169.3 (C=O), 152.5 (Ar-C), 152.4 (Ar-C), 137.4 (Ar-C), 136.3 (Ar-C), 133.0 (Ar-C), 129.0 (3 × Ar-C), 128.0 (Ar-C), 122.0 (Ar-C), 114.5 (Ar-C), 61.5 [N(CH_2_)CO], 52.6 (piperazine, 2 × NCH_2_), 47.8 (piperazine, 2 × NCH_2_). HRMS: m/z [M+2H]^+^ calculated: 377.1244; found: 377.3023.

#### 3.3.5 N-(2-Bromophenyl)-2-(4-(3-nitropyridin-2-yl)piperazin-1-yl)acetamide (**5e**)

Yield 51%; Yellow solid; m.p. 109°C–110°C. IR: ν (cm^−1^): 3,260 (NH), 1,694 (C=O), 1,587 (NO_2_), 1,551 (C=C), 1,507 (CH_2_), 1,433 (C=N), 1,232 (C-N). ^1^H NMR (400 MHz) δ (ppm): 9.99 (s, 1H, NH), 8.30–8.32 (m, 1H, Ar-H), 8.17 (dd, 1H, J= 8.1 Hz, 1.7 Hz, Ar-H), 8.87–8.88 (m, 1H, Ar-H), 7.43–7.45 (m, 1H, Ar-H), 7.23–7.25 (m, 2H, Ar-H), 6.85–6.87 (m, 1H, Ar-H), 3.37 (Br s, 4H, piperazinyl), 3.16 (s, 2H, methylene), 2.56 (Br s, 4H, piperazinyl). ^1^H NMR (400 MHz) δ (ppm): 9.97 (s, 1H, NH), 8.29-8.31 (m, 1H, Ar-H), 8.15 (dd, 1H, J= 8.1 Hz, 1.7 Hz, Ar-H), 8.85–8.88 (m, 1H, Ar-H), 7.44–7.46 (m, 1H, Ar-H), 7.22–7.25 (m, 2H, Ar-H), 6.84–6.86 (m, 1H, Ar-H), 3.35 (Br s, 4H, piperazinyl), 3.15 (s, 2H, methylene), 2.56 (Br s, 4H, piperazinyl l). HRMS: m/z [M+H]^+^ calculated: 420.0666; found: 420.0865.

#### 3.3.6 N-(3-Bromophenyl)-2-(4-(3-nitropyridin-2-yl)piperazin-1-yl)acetamide (**5f**)

Yield 50%; Orange-yellow solid; m.p. 101°C–102°C. IR: ν (cm^−1^): 3,254 (NH), 1,670 (C=O), 1,593 (NO_2_), 1,552 (C=C), 1,489 (CH_2_), 1,429 (C=N), 1,236 (C-N). ^1^H NMR (400 MHz) δ (ppm): 9.97 (s, 1H, NH), 8.29-8.31 (m, 1H, Ar-H), 8.15 (dd, 1H, J= 8.1 Hz, 1.7 Hz, Ar-H), 8.85–8.88 (m, 1H, Ar-H), 7.44–7.46 (m, 1H, Ar-H), 7.22–7.25 (m, 2H, Ar-H), 6.84–6.86 (m, 1H, Ar-H), 3.35 (Br s, 4H, piperazinyl), 3.15 (s, 2H, methylene), 2.56 (Br s, 4H, piperazinyl l)^13^C NMR (δ): 169.2 (C=O), 151.9 (2 × Ar-C), 139.0 (Ar-C), 136.0 (Ar-C), 132.6 (Ar-C), 130.7 (Ar-C), 126.8 (Ar-C), 122.4 (Ar-C), 121.4 (Ar-C), 118.8 (Ar-C), 114.2 (Ar-C), 60.6 [N(CH_2_)CO], 51.8 (piperazine, 2 × NCH_2_), 47.2 (piperazine, 2 × NCH_2_).

#### 3.3.7 N-(4-Bromophenyl)-2-(4-(3-nitropyridin-2-yl)piperazin-1-yl)acetamide (**5g**)

Yield 53%; Yellow solid; m.p. 115°C–116°C. IR: ν (cm^−1^): 3,334 (NH), 1,686 (C=O), 1,593 (NO_2_), 1,552 (C=C), 1,485 (CH_2_), 1,431 (C=N), 1,239 (C-N). ^1^H NMR (400 MHz) δ (ppm): 9.96 (s, 1H, NH), 8.31 (m, 1H, Ar-H), 8.18–8.21 (m, 1H, Ar-H), 7.46 (t, 4H, J= 9.15 Hz, Ar-H), 6.86–6.88 (m, 1H, Ar-H), 3.37 (Br s, 4H, piperazinyl), 3.31 (s, 2H, methylene), 2.57 (Br s, 4H, piperazinyl). HRMS: m/z [M+H]^+^ calculated: 420.0666; found: 420.1088.

#### 3.3.8 N-(2-Nitrophenyl)-2-(4-(3-nitropyridin-2-yl)piperazin-1-yl)acetamide (**5h**)

Yield 65%; Pale-yellow solid; m.p. 113°C–115°C. IR: ν (cm^−1^): 3,185 (NH), 1,687 (C=O), 1,597 (NO_2_), 1,556 (C=C), 1,492 (CH_2_), 1,435 (C=N), 1,226 (C-N). ^1^H NMR (400 MHz) δ (ppm): 11.55 (s, 1H, NH), 8.58 (dd, 1H, J= 8.5 Hz, 1.33 Hz, Ar-H), 8.42–8.43 (m, 1H, Ar-H), 8.26 (dd, 1H, J= 8.05 Hz, 1.7 Hz, Ar-H), 8.19 (dd, 1H, J= 8.4 Hz, 1.55 Hz, Ar-H), 7.76–7.79 (m, 1H, Ar-H), 7.30–7.33 (m, 1H, Ar), 6.92–6.96 (m, 1H, Ar-H), 3.48 (Br s, 4H, piperazinyl), 3.26 (s, 2H, methylene), 2.68 (Br s, 4H, piperazinyl). ^13^C NMR (δ): 170.1 (C=O), 152.6 (Ar-C), 152.5 (Ar-C), 137.8 (Ar-C), 136.3 (Ar-C), 136.1 (Ar-C), 133.9 (Ar-C), 133.2 (Ar-C), 126.2 (Ar-C), 124.3 (Ar-C), 122.3 (Ar-C), 114.6 (Ar-C), 61.8 [N(CH_2_)CO], 52.9 (piperazine, 2 × NCH_2_), 48.1 (piperazine, 2 × NCH_2_). HRMS: m/z [M+H]^+^ calculated: 387.1411; found: 387.3554.

#### 3.3.9 N-(3-Nitrophenyl)-2-(4-(3-nitropyridin-2-yl)piperazin-1-yl)acetamide (**5i**)

Yield 58%; Orange-yellow solid; m.p. 139°C–141°C. IR: ν (cm^−1^): 3,276 (NH), 1,690 (C=O), 1,597 (NO_2_), 1,552 (C=C), 1,481 (CH_2_), 1,431 (C=N), 1,232 (C-N). ^1^H NMR (400 MHz) δ (ppm): 10.28 (s, 1H, NH), 8.69–8.70 (m, 1H, Ar-H), 8.40–8.41 (m, 1H, Ar-H), 8.24 (dd, 1H, J= 8.05 Hz, 1.7 Hz, Ar-H), 8.02–8.04 (m, 1H, Ar-H), 7.91–7.94 (m, 1H, Ar-H), 7.61 (t, 1H, J= 8.2 Hz, Ar-H), 6.89-6.91 (m, 1H, Ar-H), 3.46 (Br s, 4H, piperazinyl), 3.25 (s, 2H, methylene), 2.63 (Br s, 4H, piperazinyl). ^13^C NMR (δ): 169.6 (C=O), 152.5 (Ar-C), 152.5 (Ar-C), 148.4 (Ar-C), 140.2 (Ar-C), 136.3 (Ar-C), 133.0 (Ar-C), 130.5 (Ar-C), 126.2 (Ar-C), 118.5 (Ar-C), 114.3 (Ar-C), 61.8 [N(CH_2_)CO], 52.8 (piperazine, 2 × NCH_2_), 47.8 (piperazine, 2 × NCH_2_). HRMS: m/z [M+H]^+^ calculated: 387.1411; found: 387.1437.

#### 3.3.10 N-(4-Nitrophenyl)-2-(4-(3-nitropyridin-2-yl)piperazin-1-yl)acetamide (**5j**)

Yield 60%; Pale-yellow solid; m. p. 145°C–146°C. IR: ν (cm^−1^): 3,211 (NH), 1,694 (C=O), 1,589 (NO_2_), 1,552 (C=C), 1,500 (CH_2_), 1,448 (C=N), 1,236 (C-N). ^1^H NMR (400 MHz) δ (ppm): 10.28 (s, 1H, NH), 8.69–8.70 (m, 1H, Ar-H), 8.40–8.41 (m, 1H, Ar-H), 8.24 (dd, 1H, J= 8.05 Hz, 1.7 Hz, Ar-H), 8.02–8.04 (m, 1H, Ar-H), 7.91–7.94 (m, 1H, Ar-H), 7.61 (t, 1H, J= 8.2 Hz, Ar-H), 6.89–6.91 (m, 1H, Ar-H), 3.49 (Br s, 4H, piperazinyl), 3.27 (s, 2H, methylene), 2.63 (Br s, 4H, piperazinyl). HRMS: m/z [M+H]^+^ calculated: 387.1339; found: 387.1550.

#### 3.3.11 2-(4-(3-Nitropyridin-2-yl)piperazin-1-yl)-N-(ortho-tolyl)acetamide (**5k**)

Yield 58%; Yellow solid; m.p. 105°C–106°C. IR: ν (cm^−1^): 3,304 (NH), 1,685 (C=O), 1,586 (NO_2_), 1,554 (C=C), 1,513 (CH_2_), 1,452 (C=N), 1,238 (C-N). ^1^H NMR (400 MHz) δ (ppm): 9.43 (s, 1H, NH), 8.41–8.43 (m, 1H, Ar-H), 8.26 (dd, 1H, J= 8.1 Hz, 1.7 Hz, Ar-H), 7.72 (dd, 1H, J= 8.05 Hz, 1.35 Hz, Ar-H), 7.16–7.24 (m, 2H, Ar-H), 7.04–7.08 (m, 1H, Ar-H), 6.91–6.93 (m, 1H, Ar-H), 3.51 (Br s, 4H, piperazinyl), 3.19 (s, 2H, methylene), 2.66 (Br s, 4H, piperazinyl), 2.24 (s, 3H, methyl). HRMS: m/z [M+2H]^+2^ calculated: 357.1790; found: 357.1865.

#### 3.3.12 2-(4-(3-Nitropyridin-2-yl)piperazin-1-yl)-N-(meta-tolyl)acetamide (**5L**)

Yield 64%; Orange-yellow solid; m.p. 95°C–96°C. IR: ν (cm^−1^): 3,301 (NH), 1,686 (C=O), 1,591 (NO_2_), 1,551 (C=C), 1,507 (CH_2_), 1,433 (C=N), 1,231 (C-N). ^1^H NMR (400 MHz) δ (ppm): 9.71 (s, 1H, NH), 8.39–8.40 (m, 1H, Ar-H), 8.24 (dd, 1H, J= 8.05 Hz, 1.7 Hz, Ar-H), 7.43–7.47 (m, 2H, Ar-H), 7.18 (t, 1H, J= 7.75 Hz, Ar-H), 6.87–6.89 (m, 2H, Ar-H), 3.44 (Br s, 4H, piperazinyl), 3.17 (s, 2H, methylene), 2.61 (Br s, 4H, piperazinyl), 2.27 (s, 3H, methyl). HRMS: m/z [M+H]^+^ calculated: 356.1717; found: 356.2223.

#### 3.3.13 2-(4-(3-Nitropyridin-2-yl)piperazin-1-yl)-N-(para-tolyl)acetamide (**5m**)

Yield 54%; Yellow solid; m.p. 112°C–113°C. IR: ν (cm^−1^): 3,295 (NH), 1,683 (C=O), 1,588 (NO_2_), 1,551 (C=C), 1,505 (CH_2_), 1,448 (C=N), 1,236 (C-N). ^1^H NMR (400 MHz) δ (ppm): 9.74 (s, 1H, NH), 8.29–8.30 (m, 1H, Ar-H), 8.16 (dd, 1H, J= 8.05 Hz, 1.65 Hz, Ar-H), 7.36–7.38 (m, 2H, Ar-H), 7.07 (d, 2H, J= 8.25 Hz, Ar-H), 6.84–6.86 (m, 1H, Ar-H), 3.46 (Br s, 4H, piperazinyl), 3.13 (s, 2H, methylene), 2.55 (Br s, 4H, piperazinyl), 2.18 (s, 3H, methyl). ^13^C NMR (δ): 169.1 (C=O), 152.5 (Ar-C), 152.4 (Ar-C), 136.4 (Ar-C), 135.5 (Ar-C), 134.0 (2 × Ar-C), 133.0 (Ar-C), 129.6 (2 × Ar-C), 120.6 (Ar-C), 114.6 (Ar-C), 61.4 [N(CH_2_)CO], 55.9 (CH_3_), 52.5 (piperazine, 2 × NCH_2_), 47.8 (piperazine, 2 × NCH_2_). HRMS: m/z [M+H]^+^ calculated: 356.1644; found: 356.2183.

#### 3.3.14 N-(2-Methoxyphenyl)-2-(4-(3-nitropyridin-2-yl)piperazin-1-yl)acetamide (**5n**)

Yield 53%; Orange-yellow solid; m.p. 120°C–121°C. IR: ν (cm^−1^): 3,301 (NH), 1,683 (C=O), 1,593 (NO_2_), 1,556 (C=C), 1,504 (CH_2_), 1,437 (C=N), 1,221 (C-N). ^1^H NMR (400 MHz) δ (ppm): 9.73 (s, 1H, NH), 8.42–8.43 (m, 1H, Ar-H), 8.27 (dd, 1H, J= 8.1 Hz, 1.65 Hz, Ar-H), 8.18 (d, 1H, J= 7.9 Hz, Ar-H), 7.06–7.07 (m, 2H, Ar-H), 6.92–6.95 (m, 2H, Ar-H), 3.88 (s, 3H, methoxy), 3.44 (Br s, 4H, piperazinyl), 3.19 (s, 2H, methylene), 2.64 (Br s, 4H, piperazinyl). ^13^C NMR (δ): 167.8 (C=O), 152.0 (Ar-C), 151.9 (Ar-C), 148.2 (Ar-C), 135.8 (Ar-C), 132.6 (Ar-C), 126.0 (Ar-C), 123.9 (Ar-C), 120.5 (Ar-C), 119.0 (Ar-C), 114.1 (Ar-C), 110.9 (Ar-C), 61.0 [N(CH_2_)CO], 55.9 (CH_3_), 52.8 (piperazine, 2 × NCH_2_), 47.8 (piperazine, 2 × NCH_2_). HRMS: m/z [M+2H]^+2^ calculated: 373.1739; found: 373.1768.

#### 3.3.15 N-(4-Methoxyphenyl)-2-(4-(3-nitropyridin-2-yl)piperazin-1-yl)acetamide (**5o**)

Yield 64%; Orange-yellow solid; m.p. 107°C–108°C. IR: ν (cm^−1^): 3,310 (NH), 1,677 (C=O), 1,590 (NO_2_), 1,552 (C=C), 1,508 (CH_2_), 1,437 (C=N), 1,236 (C-N). ^1^H NMR (400 MHz) δ (ppm): 9.65 (s, 1H, NH), 8.39−8.40 (m, 1H, Ar-H), 8.23−8.25 (m, 1H, Ar-H), 7.52−7.55 (m, 2H, Ar-H), 6.86−6.91 (m, 3H, Ar-H), 3.48 (s, 3H, methoxy), 3.44 (Br s, 4H, piperazinyl), 3.15 (s, 2H, methyl), 2.60 (Br s, 4H, piperazinyl). ^13^C NMR (δ) 168.1 (C=O), 155.9 (Ar-C), 152.5 (Ar-C), 152.4 (Ar-C), 136.3 (Ar-C), 133.0 (Ar-C), 132.1 (Ar-C), 121.7 (2 × Ar-C), 114.3 (Ar-C), 114.2 (2 × Ar-C), 61.8 [N(CH_2_)CO], 55.6(OCH_3_), 52.8 (piperazine, 2 × NCH_2_), 47.9 (piperazine, 2 × NCH_2_). HRMS: m/z [M+H]^+^ calculated: 372.1666; found: 372.1987.

### 3.4 Procedure for the synthesis of N-aryl-2-(4-(3-nitropyridin-2-yl)piperazin-1-yl)propanamide derivatives **7a-7k**

A solution of 2-chloro-N-arylpropanamides **6a-6k** (0.15 mmol) was prepared in 10 mL of acetonitrile and added to the stirred mixture of 1-(3-nitropyridin-2-yl)piperazine (**3**) (0.15 mmol) and potassium carbonate (0.3 mmol) in 15 mL of acetonitrile. The reaction mixture was refluxed for 24–48 h on stirring by monitoring with TLC. On the addition of water colored (yellow to red) precipitates of N-aryl-2-(4-(3-nitropyridin-2-yl)piperazin-1-yl)propanamide derivatives **7a-7k** was formed. The produced solid was collected, and purified by column chromatography. The calculated yield was found to be 30%–55%.

#### 3.4.1 2-(4-(3-Nitropyridin-2-yl)piperazin-1-yl)-N-phenylpropanamide (**7a**)

Yield 43%; Yellow solid; m.p. 92°C–94°C. IR: ν (cm^−1^): 3,323 (NH), 1,690 (C=O), 1,593 (NO_2_), 1,556 (C=C), 1,487 (CH_2_), 1,433 (C=N), 1,238 (C-N). ^1^H NMR (400 MHz) δ (ppm): 9.93 (s, 1H, NH), 8.29–8.31 (m, 1H, Ar-H), 8.15–8.17 (m, 1H, Ar-H), 7.51 (d, 2H, J= 8 Hz, Ar-H), 7.28 (t, 2H, J= 7.75 Hz, Ar-H), 7.04–7.07 (m, 1H, Ar-H), 6.84–6.86 (m, 1H, Ar-H), 3.35 (Br s, 4H, piperazinyl), 3.25 (q, 1H, J = 6.70 Hz, CH), 2.54–2.63 (m, 4H, piperazinyl), 1.18 (d, 3H, J= 6.9 Hz, methyl). HRMS: m/z [M+H]^+^ calculated: 356.1717; found: 356.2021.

#### 3.4.2 N-(3-Chlorophenyl)-2-(4-(3-nitropyridin-2-yl)piperazin-1-yl)propanamide (**7b**)

Yield 38%; Yellow solid; m.p. 107°C–109°C. IR: ν (cm^−1^): 3,291 (NH), 1,694 (C=O), 1,590 (NO_2_), 1,556 (C=C), 1,504 (CH_2_), 1,429 (C=N), 1,232 (C-N). ^1^H NMR (400 MHz) δ (ppm): 10.06 (s, 1H, NH), 8.32–8.33 (m, 1H, Ar-H), 8.19 (dd, 1H, J= 8.15 Hz, 1.7 Hz, Ar-H), 7.98 (dd, 1H, J= 8.1 Hz, 1.6 Hz, Ar-H), 7.47 (dd, 1H, J= 8.1 Hz, 1.45 Hz, Ar-H), 7.29–7.33 (m, 1H, Ar-H), 7.12–7.15 (m, 1H, Ar-H), 6.87–6.90 (m, 1H, Ar), 3.40 (Br s, 4H, piperazinyl), 3.27 (q, 1H, J = 6.70 Hz, CH), 2.57–2.65 (m, 4H, piperazinyl), 1.18 (d, 3H, J= 7 Hz, methyl).

#### 3.4.3 N-(4-Chlorophenyl)-2-(4-(3-nitropyridin-2-yl)piperazin-1-yl)propanamide (**7c**)

Yield 42%; Red-orange solid; m.p. 57°C–59°C. IR: ν (cm^−1^): 3,278 (NH), 1,673 (C=O), 1,595 (NO_2_), 1,556 (C=C), 1,487 (CH_2_), 1,429 (C=N), 1,239 (C-N). ^1^H NMR (400 MHz) δ (ppm): 10.04 (s, 1H, NH), 8.29–8.30 (m, 1H, Ar-H), 8.15 (dd, 1H, J= 8.1 Hz, 1.7 Hz, Ar-H), 7.53–7.57 (m, 2H, Ar-H), 7.53–7.56 (m, 2H, Ar-H), 6.83–6.85 (m, 1H, Ar-H), 3.34 (Br s, 4H, piperazinyl), 3.25 (q, 1H, J = 6.90 Hz, CH), 2.52–2.61 (m, 4H, piperazinyl), 1.16 (d, 3H, J= 6.85, methyl). ^13^C NMR (δ): 172.3 (C=O), 152.5 (Ar-C), 152.4 (Ar-C), 137.3 (Ar-C), 136.3 (Ar-C), 133.0 (Ar-C), 129.0 (2 × Ar-C), 128.1 (Ar-C), 122.0 (2 × Ar-C), 114.5 (Ar-C), 63.6 [N(CH)CO], 49.2 (piperazine, 2 × NCH_2_), 48.1 (piperazine, 2 × NCH_2_), 13.4 (CH_3_). HRMS: m/z [M+H]^+^ calculated: 390.1327; found: 390.2126.

#### 3.4.4 N-(2-Bromophenyl)-2-(4-(3-nitropyridin-2-yl)piperazin-1-yl)propanamide (**7d**)

Yield 45%; Yellow solid; m.p. 91°C–94°C. IR: ν (cm^−1^): 3,289 (NH), 1,692 (C=O), 1,587 (NO_2_), 1,556 (C=C), 1,503 (CH_2_), 1,429 (C=N), 1,234 (C-N). ^1^H NMR (400 MHz) δ (ppm): 10.04 (s, 1H, NH), 8.32–8.34 (m, 1H, Ar-H), 8.19 (dd, 1H, J= 8.05 Hz, 2.05 Hz, Ar-H), 8.00 (dd, 1H, J= 8.15 Hz, 1.55 Hz, Ar-H), 7.62 (dd, 1H, J= 8 Hz, 1.3 Hz, Ar-H), 7.33–7.36 (m, 1H, Ar-H), 7.05–7.08 (m, 1H, Ar-H), 6.87–6.90 (m, 1H, Ar-H), 3.43 (Br s, 4H, piperazinyl), 3.36 (q, 1H, J = 6.75 Hz, CH), 2.58–2.65 (m, 4H, piperazinyl), 1.18 (d, 3H, J= 6.95 Hz, methyl). HRMS: m/z [M+H]^+^ calculated: 434.0822; found: 434.2365.

#### 3.4.5 N-(2-Nitrophenyl)-2-(4-(3-nitropyridin-2-yl)piperazin-1-yl)propanamide (**7e**)

Yield 55%; Yellow solid; m.p. 124°C–125°C. IR: ν (cm^−1^): 3,293 (NH), 1,698 (C=O), 1,592 (NO_2_), 1,560 (C=C), 1,489 (CH_2_), 1,420 (C=N), 1,221 (C-N). ^1^H NMR (400 MHz) δ (ppm): 11.6 (s, 1H, NH), 8.55 (dd, 1H, J= 8.45 Hz, 1.35 Hz, Ar-H), 8.40–8.41 (m, 1H, Ar-H), 8.24–8.28 (m, 1H, Ar-H), 8.17 (dd, 1H, J= 8.4 Hz, 1.55 Hz, Ar-H), 7.74–7.78 (m, 1H, Ar-H), 7.29–7.32 (m, 1H, Ar-H), 6.90–6.93 (m, 1H, Ar-H), 3.53–3.60 (m, 4H, piperazinyl), 3.45 (q, 1H, J = 6.70 Hz, CH), 2.59–2.68 (m, 4H, piperazinyl), 1.19 (d, 3H, J= 7 Hz, methyl). ^13^C NMR (δ): 172.7 (C=O), 152.0 (Ar-C), 151.9 (Ar-C), 137.5 (Ar-C), 135.8 (Ar-C), 135.5 (Ar-C), 133.3 (Ar-C), 132.5 (Ar-C), 125.5 (Ar-C), 123.7 (Ar-C), 121.9 (Ar-C), 114.0 (Ar-C), 63.5 [N(CH_2_)CO], 48.7 (piperazine, 2 × NCH_2_), 47.6 (piperazine, 2 × NCH_2_), 10.2 (CH_3_). HRMS: m/z [M+H]^+^ calculated: 401.1568; found: 401.1834.

#### 3.4.6 N-(3-Nitrophenyl)-2-(4-(3-nitropyridin-2-yl)piperazin-1-yl)propanamide (**7f**)

Yield 47%; Orange solid; m.p. 120°C–121°C. IR: ν (cm^−1^): 3,221 (NH), 1,692 (C=O), 1,593 (NO_2_), 1,556 (C=C), 1,502 (CH_2_), 1,429 (C=N), 1,232 (C-N). ^1^H NMR (400 MHz) δ (ppm): 10.36 (s, 1H, NH), 8.56–8.57 (m, 1H, Ar-H), 8.25-8.28 (m, 1H, Ar-H), 8.12–8.14 (m, 1H, Ar-H), 7.85–7.87 (m, 1H, Ar-H), 7.74–7.82 (m, 1H, Ar-H), 7.51–7.55 (m, 1H, Ar-H), 6.82–6.84 (m, 1H, Ar-H), 3.34 (Br s, 4H, piperazinyl), 3.29 (q, 1H, J = 6.65 Hz, CH), 2.57-2.61 (m, 4H, piperazinyl), 1.18 (d, 3H, J= 6.9 Hz, methyl). HRMS: m/z [M+H]^+^ calculated: 401.1568; found: 401.1877.

#### 3.4.7 N-(4-Nitrophenyl)-2-(4-(3-nitropyridin-2-yl)piperazin-1-yl)propanamide (**7g**)

Yield 51%; Yellow solid; m.p. 159°C–161°C. IR: ν (cm^−1^): 3,343 (NH), 1,698 (C=O), 1,590 (NO_2_), 1,556 (C=C), 1,498 (CH_2_), 1,440 (C=N), 1,232 (C-N). ^1^H NMR (400 MHz) δ (ppm): 10.47 (s, 1H, NH), 8.38–8.39 (m, 3H, Ar-H), 8.21–8.24 (m, 2H, Ar-H), 6.88–6.90 (m, 1H, Ar-H), 3.48–3.57 (m, 4H, piperazinyl), 3.40 (q, 1H, J = 6.75 Hz, CH), 2.57–2.67 (m, 4H, piperazinyl), 1.21 (d, 3H, J= 6.85 Hz, methyl). ^13^C NMR (δ):172.7 (C=O), 152.5 (Ar-C), 152.4 (Ar-C), 145.3 (Ar-C), 142.8 (Ar-C), 136.3 (Ar-C), 133.0 (Ar-C), 125.3 (2 × Ar-C), 119.7 (2 × Ar-C), 114.3 (Ar-C), 63.8 (Ar-C), 49.2 (piperazine, 2 × NCH_2_), 48.2 (piperazine, 2 × NCH_2_), 12.7 (CH_3_). HRMS: m/z [M+2H]^+2^ calculated: 402.1495; found: 402.1550.

#### 3.4.8 N-(ortho-Tolyl)-2-(4-(3-nitropyridin-2-yl)piperazin-1-yl)propanamide (**7h**)

Yield 33%; Yellow solid; m.p. 90°C–92°C. IR: ν (cm^−1^): 3,301 (NH), 1,687 (C=O), 1,590 (NO_2_), 1,556 (C=C), 1,506 (CH_2_), 1,433 (C=N), 1,220 (C-N). ^1^H NMR (400 MHz) δ (ppm): 9.56 (s, 1H, NH), 8.31–8.32 (m, 1H, Ar-H), 8.15–8.18 (m, 1H, Ar-H), 7.44–7.45 (m, 1H, Ar-H), 7.12–7.19 (m, 2H, Ar-H), 7.04–7.07 (m, 1H, Ar-H), 6.85–6.88 (m, 1H, Ar-H), 3.37 (Br s, 4H, piperazinyl), 3.26 (q, 1H, J = 6.65 Hz, CH), 2.56–2.66 (m, 4H, piperazinyl), 2.15 (s, 3H, methyl), 1.19 (d, 3H, J= 7 Hz, methyl). HRMS: m/z [M+2H]^+2^ calculated: 371.1946; found: 371.2119.

#### 3.4.9 N-(para-Tolyl)-2-(4-(3-nitropyridin-2-yl)piperazin-1-yl)propanamide (**7i**)

Yield 41%; Orange-yellow solid; m.p. 88°C–89°C. IR: ν (cm^-1^): 3,278 (NH), 1,670 (C=O), 1,593 (NO_2_), 1,554 (C=C), 1,506 (CH_2_), 1,429 (C=N), 1,236 (C-N). ^1^H NMR (400 MHz) δ (ppm): 9.7 (s, 1H, NH), 8.38–8.40 (m, 1H, Ar-H), 8.22 (dd, 1H, J= 8 Hz, 1.8 Hz, Ar-H), 7.50–7.56 (m, 2H, Ar-H), 7.09–7.11 (m, 2H, Ar-H), 6.87–6.90 (m, 1H, Ar-H), 3.35 (Br s, 4H, piperazinyl), 3.30 (m, 1H, J = 6.90 Hz, CH), 2.55–2.67 (m, 4H, piperazinyl), 2.24 (s, 3H, methyl), 1.9 (d, 3H, J= 6.85 Hz, methyl). HRMS: m/z [M+H]^+^ calculated: 370.1874; found: 370.2142.

#### 3.4.10 N-(2-Methoxyphenyl)-2-(4-(3-nitropyridin-2-yl)piperazin-1-yl)propanamide (**7j**)

Yield 30%; Yellow solid; m.p. 108°C–110°C. IR: ν (cm^−1^): 3,304 (NH), 1,683 (C=O), 1,593 (NO_2_), 1,556 (C=C), 1,515 (CH_2_), 1,433 (C=N), 1,236 (C-N). ^1^H NMR (400 MHz) δ (ppm): 9.92 (s, 1H, NH), 8.41–8.43 (m, 1H, Ar-H), 8.2–8.29 (m, 1H, Ar-H), 8.15–8.17 (m, 1H, Ar-H), 7.03–7.07 (m, 2H, Ar-H), 6.91–6.95 (m, 2H, Ar-H), 3.88 (s, 3H, methoxy), 3.50–3.56 (m, 4H, piperazinyl), 3.37–3.42 (m, 1H, CH), 2.58–2.67 (m, 4H, piperazinyl), 1.8 (d, 3H, J= 7 Hz, methyl).

#### 3.4.11 N-(4-Methoxyphenyl)-2-(4-(3-nitropyridin-2-yl)piperazin-1-yl)propanamide (**7k**)

Yield 32%; Red solid; m.p. 62°C-64°C. IR: ν (cm^−1^): 3,301 (NH), 1,675 (C=O), 1,593 (NO_2_), 1,556 (C=C), 1,507 (CH_2_), 1,435 (C=N), 1,232 (C-N). ^1^H NMR (400 MHz) δ (ppm): 9.72 (s, 1H, NH), 8.37–8.39 (m, 1H, Ar-H), 8.21–8.23 (m, 1H, Ar-H), 7.52–7.55 (m, 2H, Ar-H), 6.86–6.89 (m, 3H, Ar-H), 3.71 (s, 3H, methoxy), 3.43 (t, 4H, J= 4.95 Hz, piperazinyl), 3.27–3.31 (m, 1H, CH), 2.56 (t, 4H, J= 5 Hz, piperazinyl), 1.9 (d, 3H, J= 6.9 Hz, methyl) ^13^C NMR (δ): 171.0 (C=O), 155.8 (Ar-C), 152.5 (Ar-C), 152.4 (Ar-C), 136.3 (Ar-C), 132.9 (Ar-C), 132.3 (Ar-C), 121.6 (2 × Ar-C), 114.8 (Ar-C), 114.2 (2× Ar-C), 63.6 [N(CH)CO], 55.6 (OCH_3_), 52.9 (piperazine, 2 × NCH_2_), 49.4 (piperazine, 2 × NCH_2_), 13.3 (CH_3_). HRMS: m/z [M+H]^+^ calculated: 386.1750; found: 386.3397.

### 3.5 *In vitro* inhibition assay of urease

The inhibitory activity of 1-(3-nitropyridin-2-yl)piperazine derivatives (**5a-o; 7a-k**) was assessed *in vitro* against urease with minor modifications using the indophenol method (Al Azzam et al., 2022; Da Costa et al., 2022). At first, 1 mM solution of a test compound was prepared in 10% DMSO followed by the preparation of the reaction mixture. The reaction mixture was prepared by adding 30 μL of reaction buffer (100 mmol/L of urea, 1 mmol/L of EDTA, 10 mmol/L of LiCl_2_, 10 mmol/L of KH_2_PO_4_; pH: 8.2), 50 μL of R1 (120 mmol/L phosphate buffer, 60 mmol/L sodium salicylate, 5 mmol/L sodium nitroprusside, 1 mmol/L EDTA and 5 KU/L urease), 10 μL of substrate (1 mM urea), and 10 μL of test compound in triplicate in a 96-well plate. After 5 min of incubation at ambient temperature, 70 μL of R2 (120 mmol/L phosphate buffer, 400 mmol/L sodium hydroxide, and 10 mmol/L sodium hypochlorite) was added to the reaction mixture (Rauf et al., 2013). After another incubation period of 10 min at room temperature, the absorbance was measured using a microplate reader (Bio-Tek, United States) at a particular wavelength of 630 nm. In this assay, thiourea was used as a positive control (standard). The following equation was used to determine the percentage of inhibition for each test compound.

$$\text{Percentage inhibition}=100–\left[\text{Absorbance of test compound}/\text{ Absorbance of negative control}\right]\times 100$$

The IC_50_ values of test compounds (**5a-o; 7a-k**) at various concentrations were determined by analyzing the data, obtained from *in vitro* urease inhibitory assay, by GraphPad Prism version 8.0.2 (GraphPad, California, United States).

### 3.6 *In vitro* hemocompatibility screening

Hemolysis is the rupturing of the cell membrane of red blood cells that causes the expulsion of hemoglobin into the blood plasma. To analyze the effects of 1-(3-nitropyridin-2-yl)piperazine **3** and its derivatives (**5b, 5c, 5i; 7e, 7h**) on human blood, a hemolysis analysis was carried out (Supplementary Figure S69). About 5 mL fresh sample of human blood was collected from a 24-year-old volunteer and transferred to a vacutainer tube containing EDTA to prevent coagulation. The ultracentrifugation of the blood sample was carried out at 3,000 rpm for 20 min and the separated plasma was discarded. The blood cells were washed about three times by using a double volume of phosphate buffer solution (PBS) (pH = 7.4). Stock solutions of 1-(3-nitropyridin-2-yl)piperazine **3** and its derivatives (**5b**, **5c**, **5i**; **7e**, **7h**) were prepared (5 mg/mL) and test samples were diluted as follows: 0.5 mg/mL, 1 mg/mL, 2.5 mg/mL, 5 mg/mL, and 10 mg/mL. For negative control reference, 1 mL PBS was added to the blood test sample while 100 µL (4%), Triton X-100 was added as a positive control reference. After that, 400 µL of blood test cells and 1 mL of PBS were taken in each Eppendorf tube and a 100 µL of 1-(3-nitropyridin-2-yl)piperazine **3** and its derivatives (**5b**, **5c**, **5i**; **7e**, **7h**) were added separately to make the final volume. All test samples were incubated at 37°C for 1 h and then kept in an ice bath for 1 min. Finally, test samples were centrifuged at 3,000 rpm for 5 min. To quantify hemoglobin in test samples, the absorbance of the supernatant was measured at 540 nm (Munawwar et al., 2023). From the absorbance value, the hemolysis percentage was determined by the following formula:

$$Hemolysis\;\%=\frac{\left(Absorbance\;of\;test\;sample-Absorbance\;of\;negative\;control\right)}{Absorbance\;of\;test\;sample-Absorbance\;of\;negative\;control}\times 100$$

### 3.7 *In silico* investigation

#### 3.7.1 Pre-clinical investigation

The toxicological and pharmacological properties of the precursor compound and potent inhibitors were determined by freely accessible software, namely, admetSAR (http://lmmd.ecust.edu.cn/admetsar1/home/, accessed on 10 September 2023) and eMolTox (http://xundrug.cn/moltox, accessed on 8 July 2023). Furthermore, GUSAR (http://www.way2drug.com/gusar/acutoxpredict.html, accessed on 11 September 2023) was used to evaluate the optimal route of administration of potent inhibitors to rats for *in vivo* studies in the future based on lethal dose 50 (LD_50_) values (Al Azzam et al., 2022). Similarly, eMolTox functions as an online platform designed to forecast the possible toxicological effects linked to the molecular configuration of a compound using an aggregate predictor method (Da Costa et al., 2022).

#### 3.7.2 Docking studies

**FIGURE 9:**
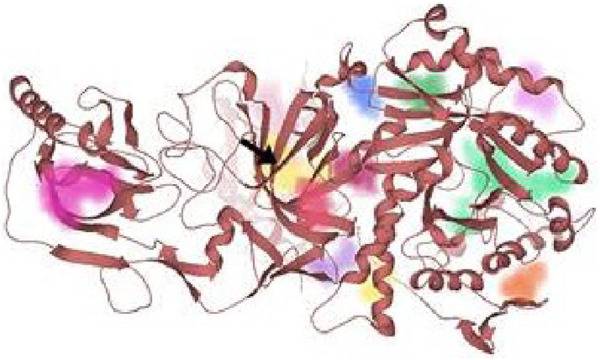
All the probable binding sites in urease. Among these binding pockets, the yellow one is the most druggable allosteric site.

AutoDockVina was employed to perform the molecular docking of the potent inhibitor with the most druggable active site of urease (Kumar et al., 2022). The most druggable active site was identified based on DoGSiteScore obtained from the analysis of target protein in the binding site mode of SeeSAR version 12.1.0, as shown in Figure 9 (Volkamer et al., 2012; BioSolveIT GmbH, 2022). Initially, the pdb structure of jack bean urease (PDB id: 4H9M), obtained from the RCSB repository, was prepared by eliminating water molecules and ligands in Discovery Studio molecular visualizer 2021. Similarly, Discovery Studio was used to convert the format of compound **3** and potent inhibitors from sdf to pdb format after minimizing their energy via Chem3D Pro 12.0.2.1076. Chem3D Pro 12.0.2.1076 utilizes Merck Molecular Force Field 2 (MM2) algorithm that iteratively adjusts the coordinates of atoms in the molecule to minimize its energy (Al Azzam et al., 2022; Moulishankar and Thirugnanasambandam, 2023). Afterward, AutoDockTools 1.5.7 was utilized to construct pdbqt files of the receptor (4H9M) and potent inhibitors followed by the formation of a grid box encompassing a druggable binding site (Fatima et al., 2022). After giving specific commands, nine different poses as output files were generated, and the best pose was selected based on the lowest binding energy. The grid dimensions were adjusted as indicated in Table 5. Later, the intermolecular interactions between the best-docked pose and the active site of the receptor were determined by BIOVIA Discovery Studio 2021. This software determines the favorable interactions of hydrogen bonds, salt bridges, and hydrophobic interactions (Baskaran et al., 2020).

**TABLE 5:** Dimensions of grid adjusted for molecular docking.

Grid dimensions					
size_x	size_y	size_z	center_x	center_y	center_z
38	42	44	−7.570	−61.549	−11.201

## 4 Conclusion

Urease plays a pivotal role in urea degradation and generates ammonia and carbamate. This enzymatic process significantly raises the pH, thereby facilitating the survival of pathogenic microorganisms. Consequently, targeting urease activity is as a crucial strategy in the treatment of such pathogen-mediated diseases. So, precursor compound **3** and derivatives of 1-(3-nitropyridin-2-yl)piperazine were evaluated by *in silico* and *in vitro* analysis. **5b** and **7e** exhibit the most inhibitory activity against, with impressive IC_50_ values of 2.0 ± 0.73 and 2.24 ± 1.63 µM, respectively. Their IC_50_ values were lower than the precursor compound **3,** whose IC_50_ value was 3.90 ± 1.91 µM. In silico analyses illustrated that both of these potent inhibitors establish favorable interactions within the active site of urease; residues Arg132, Asp295, Thr830, Arg835, and Asn836 emerge as pivotal constituents of the active site. However, none of the binding site residues interacting with precursor compound **3** resemble the interacting residues of **5b** and **7e**. The binding energies of **5b** and **7e** were −8.0 kcal/mol (**5b**) and −8.1 kcal/mol (**7e**), accordingly. Pre-clinical investigation predicted that both **5b** and **7e** may exhibit gastrointestinal permeability and cross the blood-brain barrier. In addition, both inhibitors may produce toxic substructures and have LD_50_ values of 1,360 (**5b**) and 911.1 mg/kg (**7e**). The hemolysis evaluation predicted that **7e** and **7h** are more biocompatible in the human blood stream. Therefore, **5b** and **7e** can be pivotal therapeutic interventions for the management of infections caused by microbes whose survival is dependent on urease activity. Further, *in vivo* studies are crucial to validate their clinical efficacy.

## Data Availability

The original contributions presented in the study are included in the article/Supplementary Material, further inquiries can be directed to the corresponding authors.
