# Supplementary material for: Synthesis and biological evaluation of pyridylpiperazine hybrid derivatives as urease inhibitors
Source: Front Chem. 2024 Mar 13;12:1371377. doi: 10.3389/fchem.2024.1371377 (PMC10965793; doi:10.3389/fchem.2024.1371377)
Supplement: Supplementary file 1 [file DataSheet1.PDF]

Synthesis and Biological Evaluation of Pyridylpiperazine hybrid Derivatives as Urease Inhibitors

Muhammad Akash[1], Sumera Zaib[2], Matloob Ahmad[1,*], Sadia Sultan[1], Sami A. Al-Hussain[3,*]

[1] Department of Chemistry, Government College University Faisalabad, 38000, Pakistan, chem.fsd@gmail.com

[2] Department of Basic and Applied Chemistry, Faculty of Science and Technology, University of Central Punjab, Lahore 54590, Pakistan (sumera.zaib@ucp.edu.pk)

[3] Department of Chemistry, Faculty of Science, Imam Mohammad Ibn Saud Islamic University (IMSIU), Riyadh 11623, Saudi Arabia

*** Correspondence:**
Matloob Ahmad,

Matloob.Ahmad@gcuf.edu.pk

Sami A. Al-Hussain,

Sahussain@imamu.edu.sa

Supplementary Material

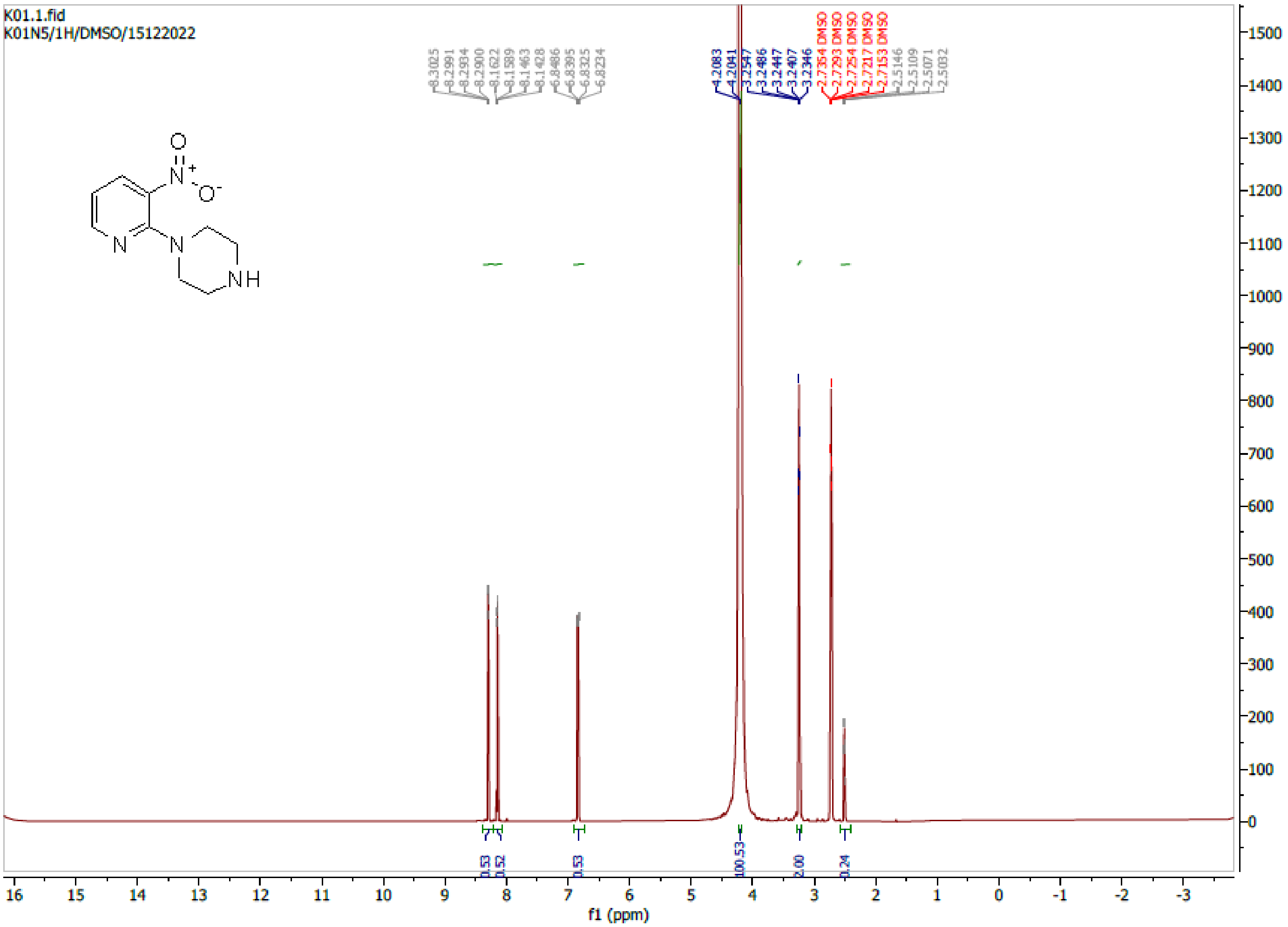

Figure S1. ^{1}HNMR of *1-(3-Nitropyridin-2-yl)piperazine* (**3**)

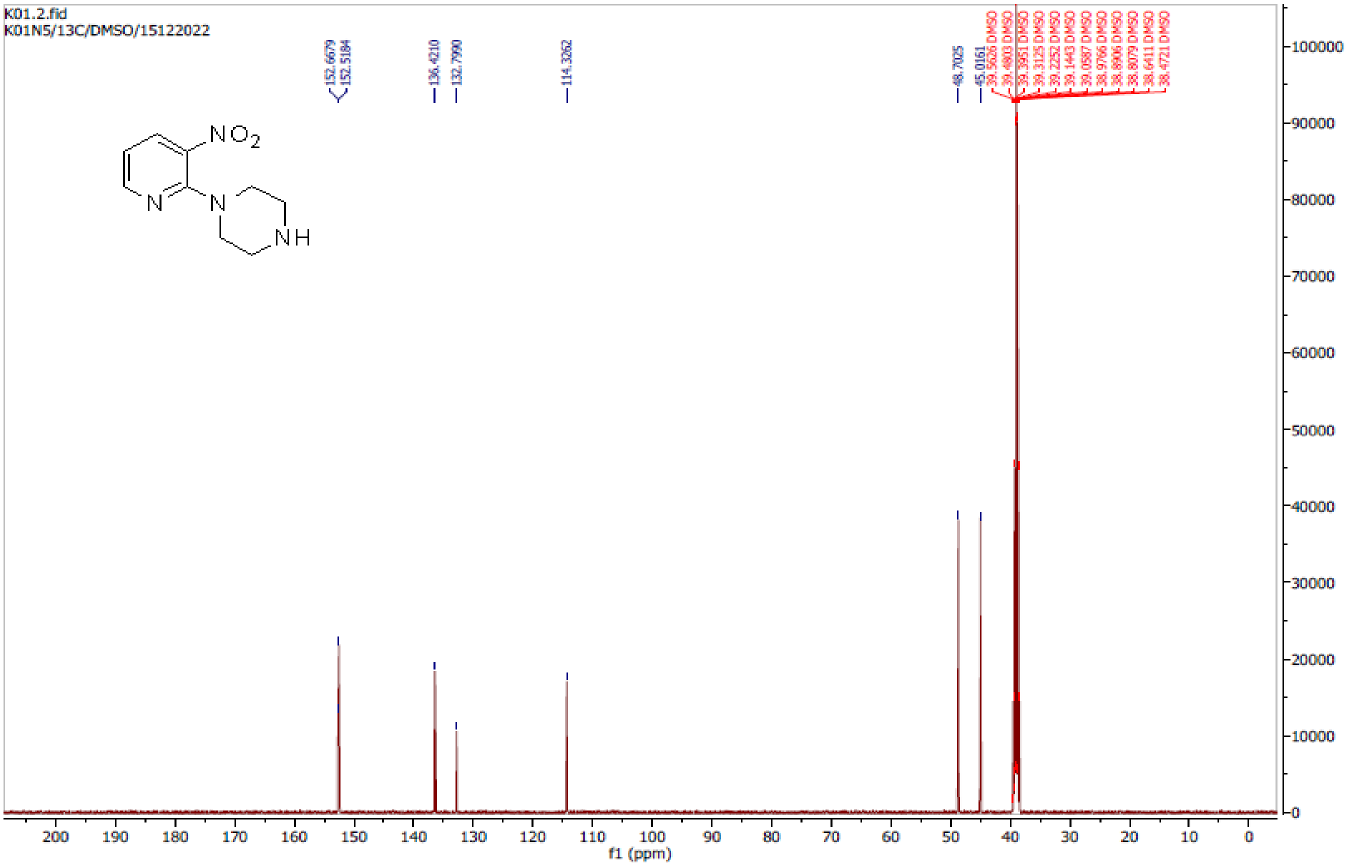

Figure S2. ^{13}CNMR of *1-(3-Nitropyridin-2-yl)piperazine* (***3***)

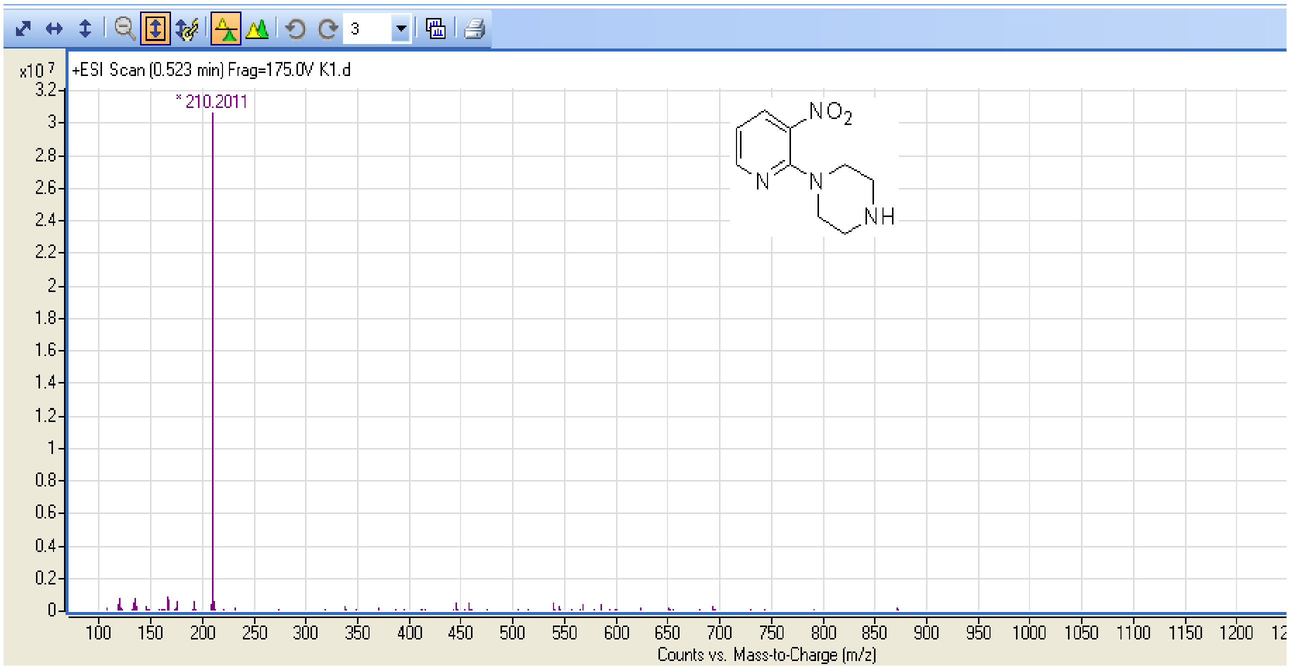

Figure S3. MS of *1-(3-Nitropyridin-2-yl)piperazine* (***3***)

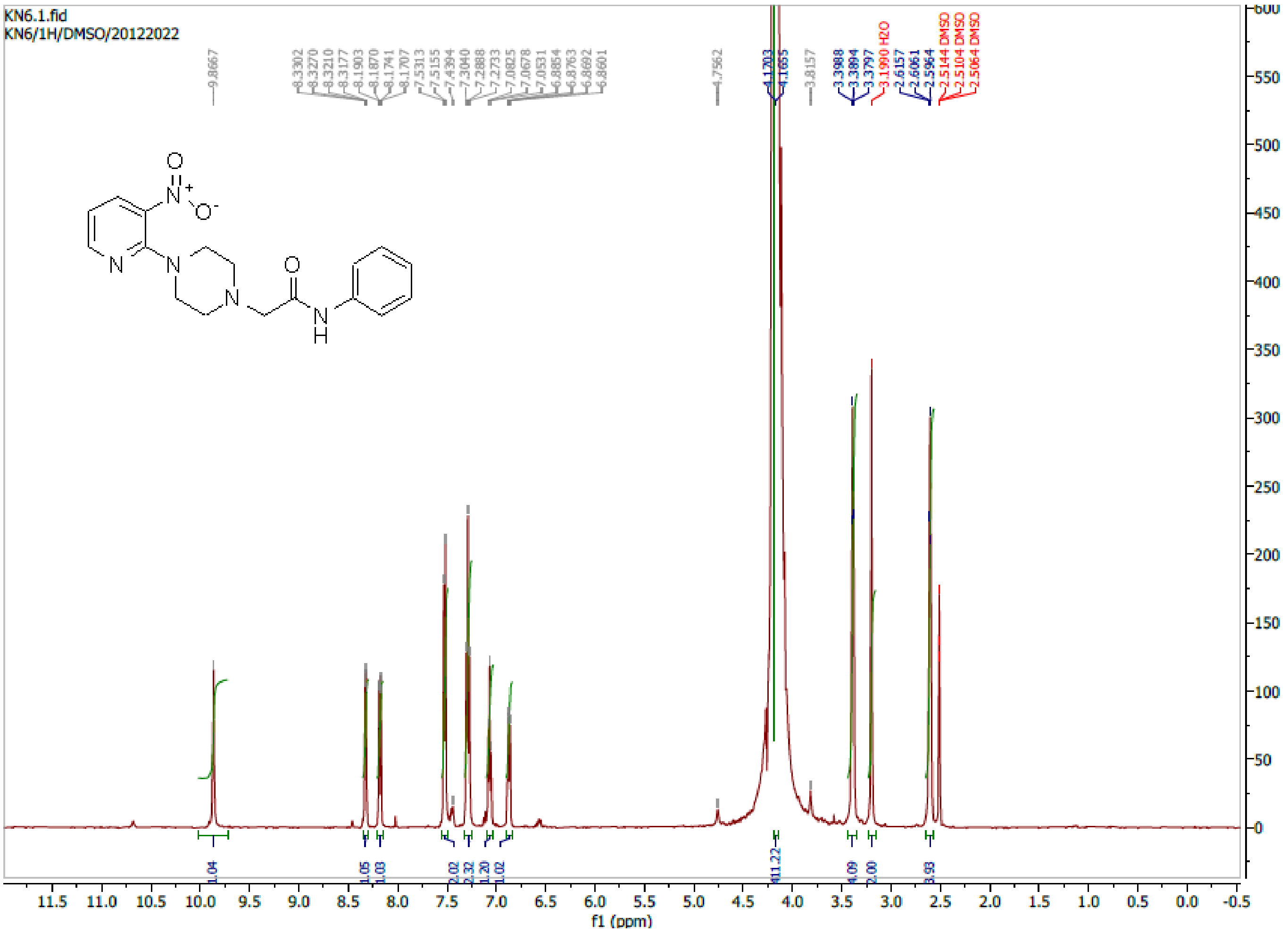

Figure S4. ^{1}HNMR of *2-(4-(3-Nitropyridin-2-yl)piperazin-1-yl)-N-phenylacetamide (**5a**)*

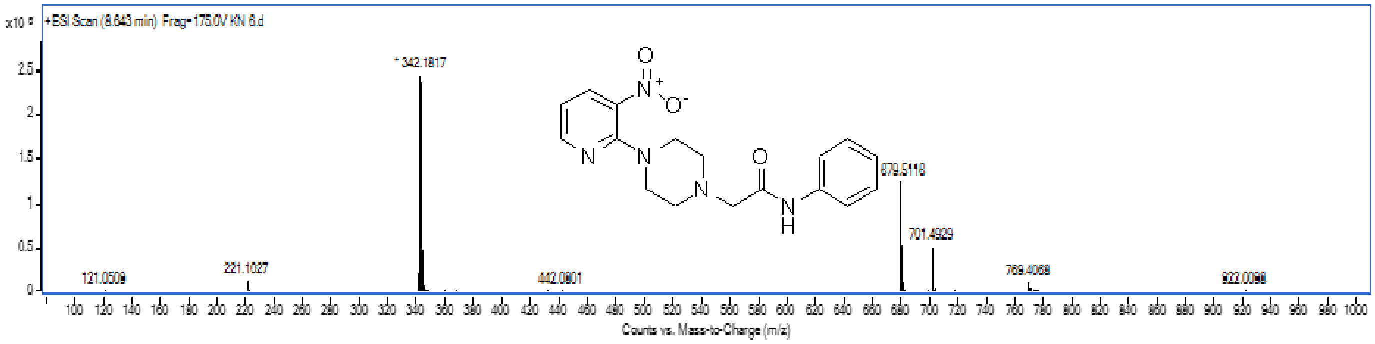

Figure S5. MS of *2-(4-(3-Nitropyridin-2-yl)piperazin-1-yl)-N-phenylacetamide* (***5a***)

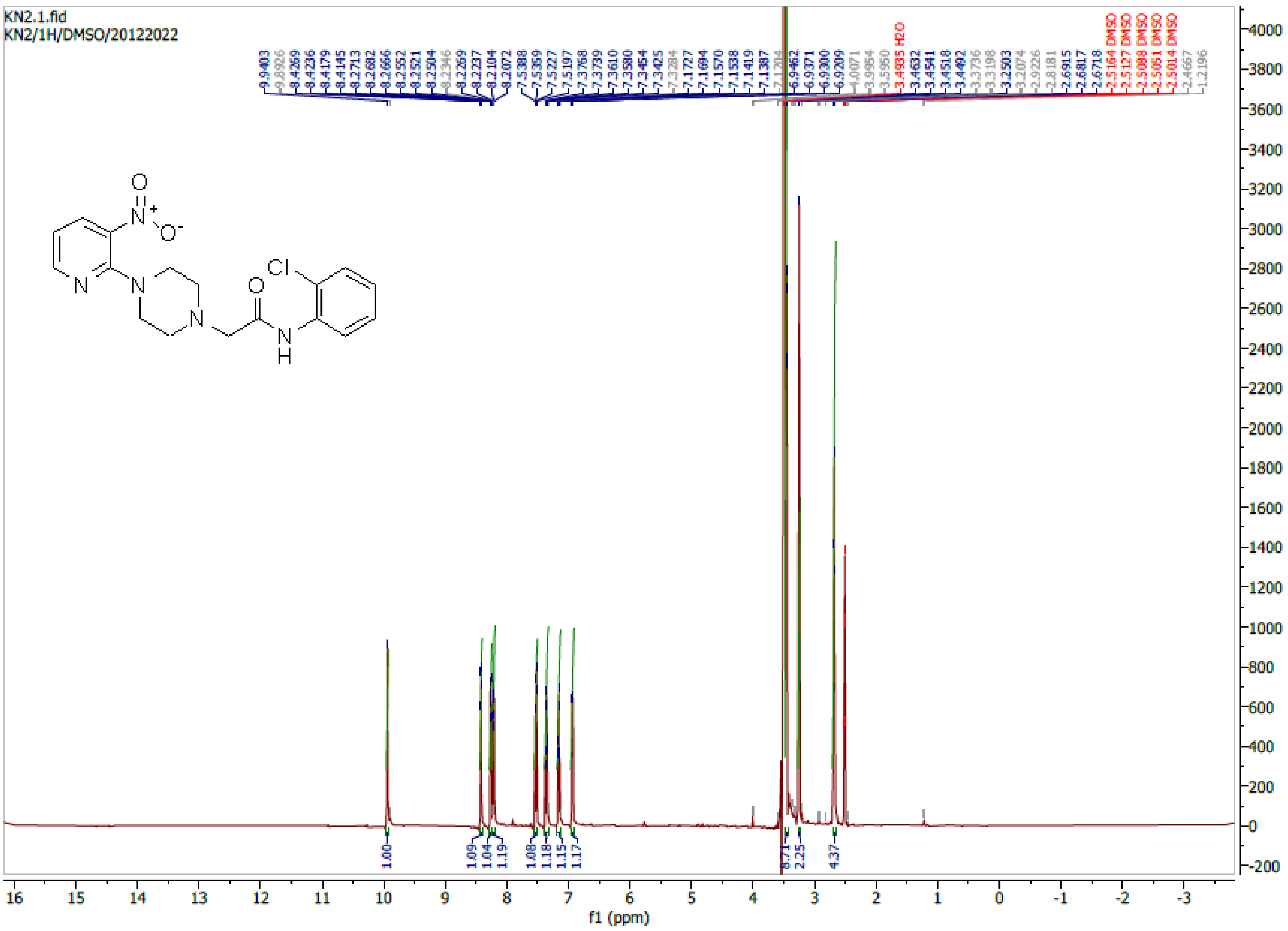

Figure S6. ^{1}HNMR of *N-(2-Chlorophenyl)-2-(4-(3-nitropyridin-2-yl)piperazin-1-yl)acetamide* (***5b***)

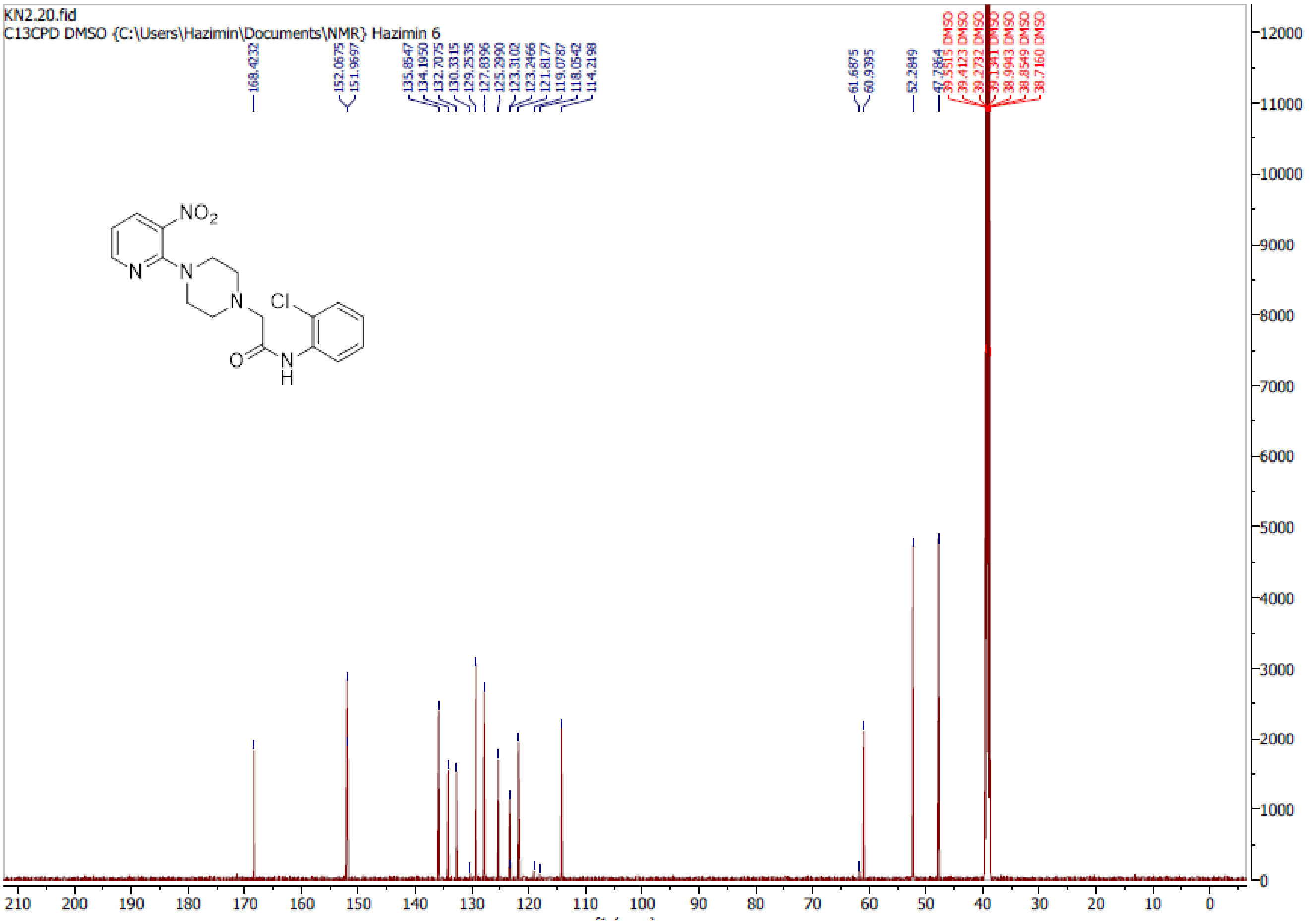

Figure S7. ^{13}CNMR of *N-(2-Chlorophenyl)-2-(4-(3-nitropyridin-2-yl)piperazin-1-yl)acetamide (**5b**)*

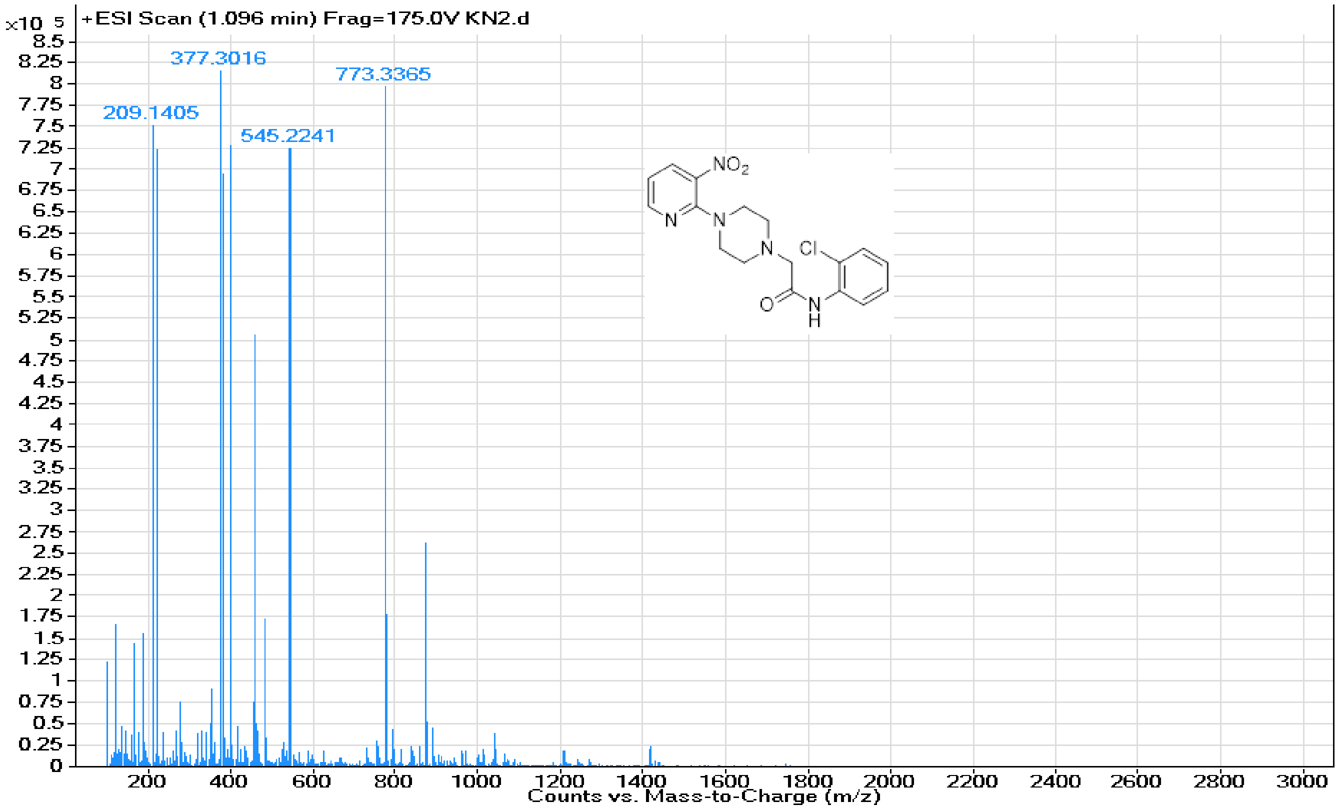

Figure S8. MS of *N-(2-Chlorophenyl)-2-(4-(3-nitropyridin-2-yl)piperazin-1-yl)acetamide (**5b**)*

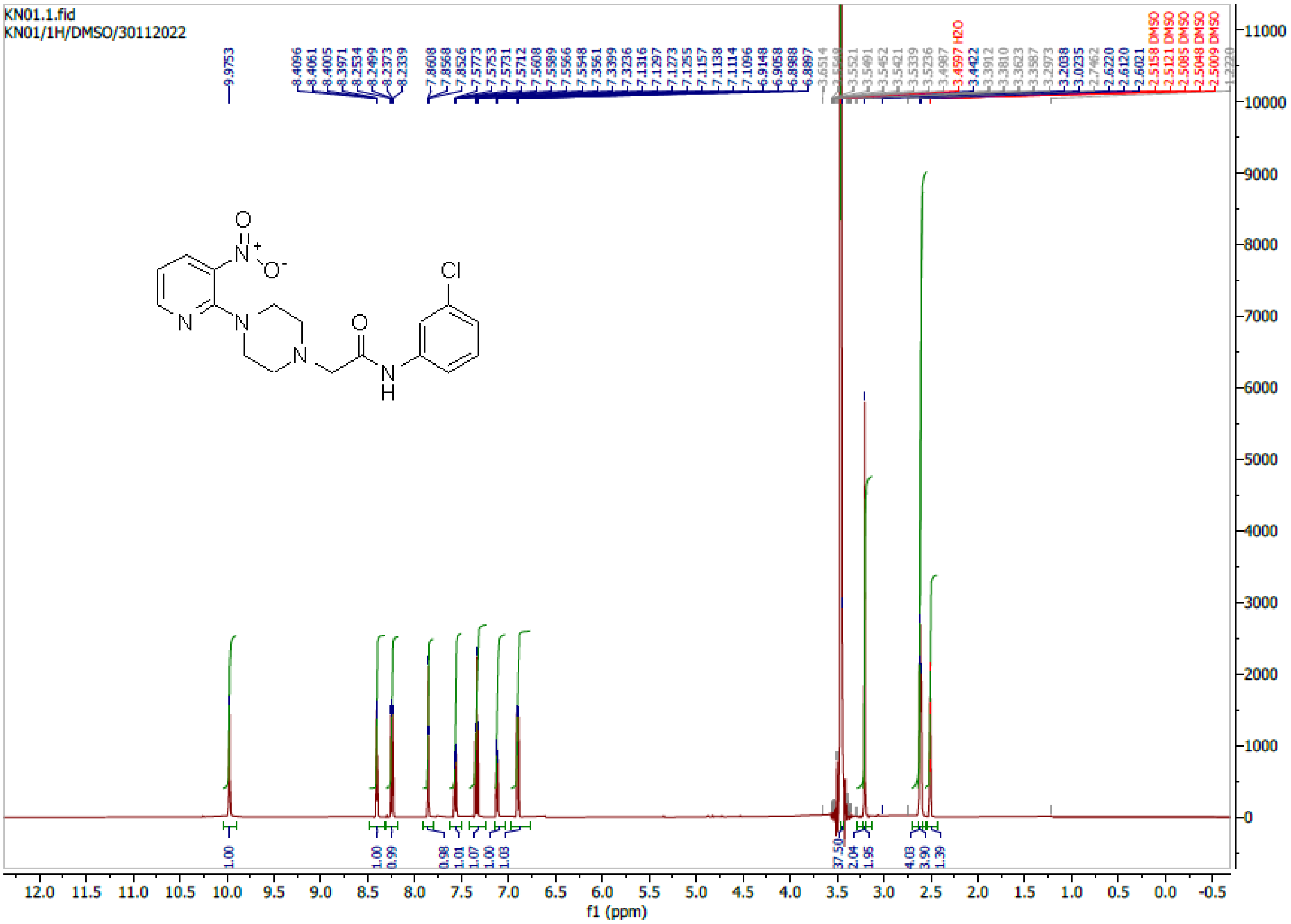

Figure S9. ¹HNMR of *N-(3-Chlorophenyl)-2-(4-(3-nitropyridin-2-yl)piperazin-1-yl)acetamide* (**5c**)

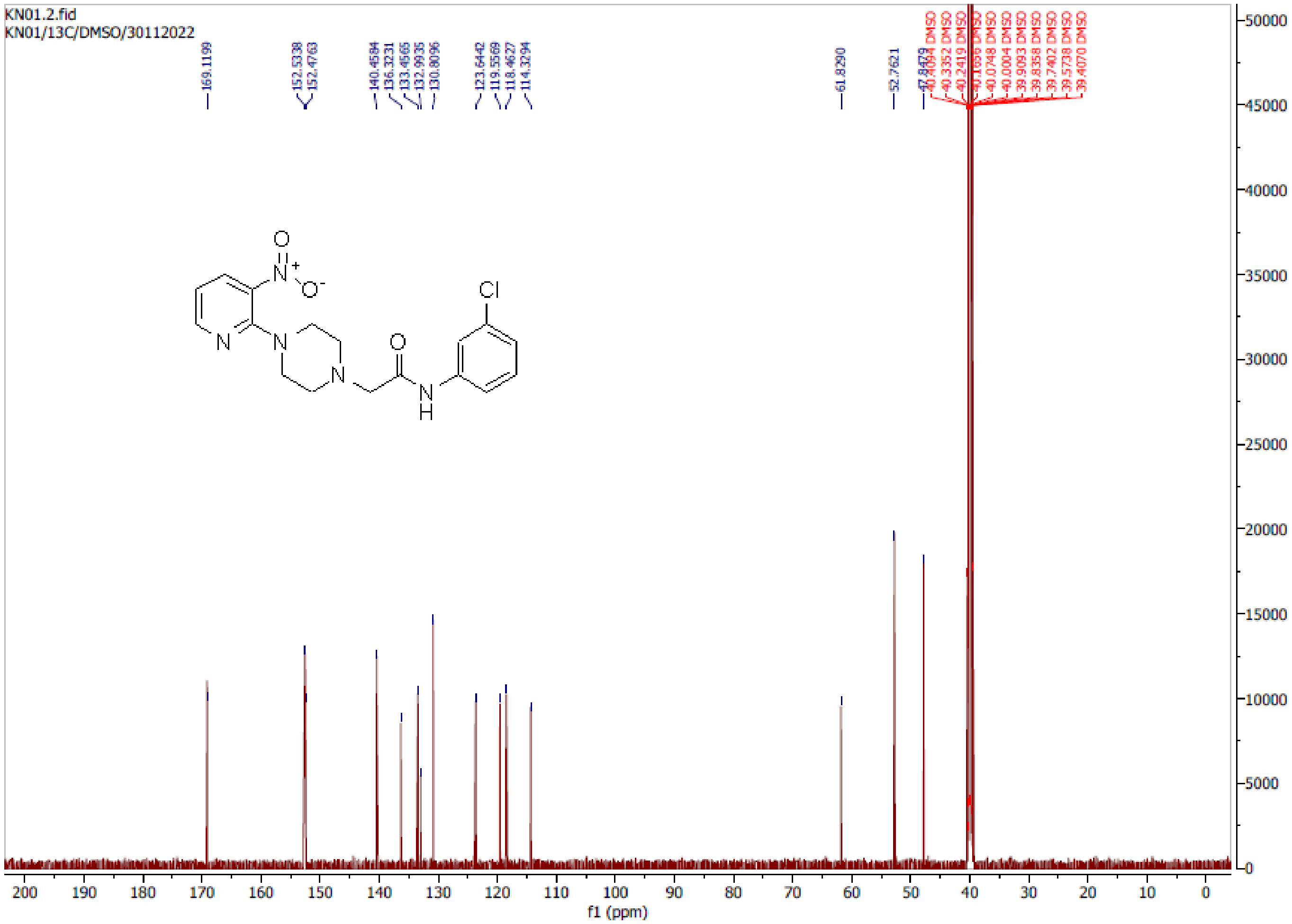

Figure S10. ^{13}CNMR of *N-(3-Chlorophenyl)-2-(4-(3-nitropyridin-2-yl)piperazin-1-yl)acetamide* (**5c**)

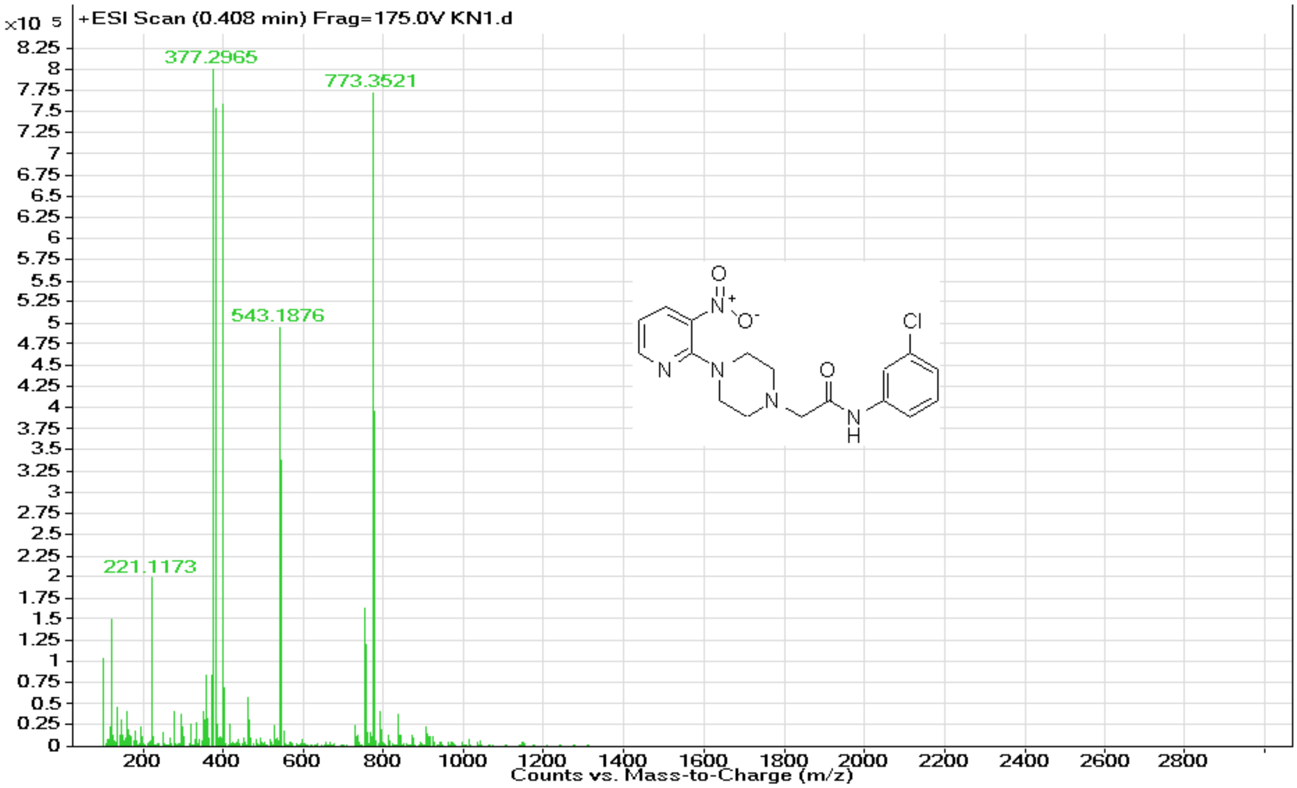

Figure S11. MS of *N-(3-Chlorophenyl)-2-(4-(3-nitropyridin-2-yl)piperazin-1-yl)acetamide (**5c**)*

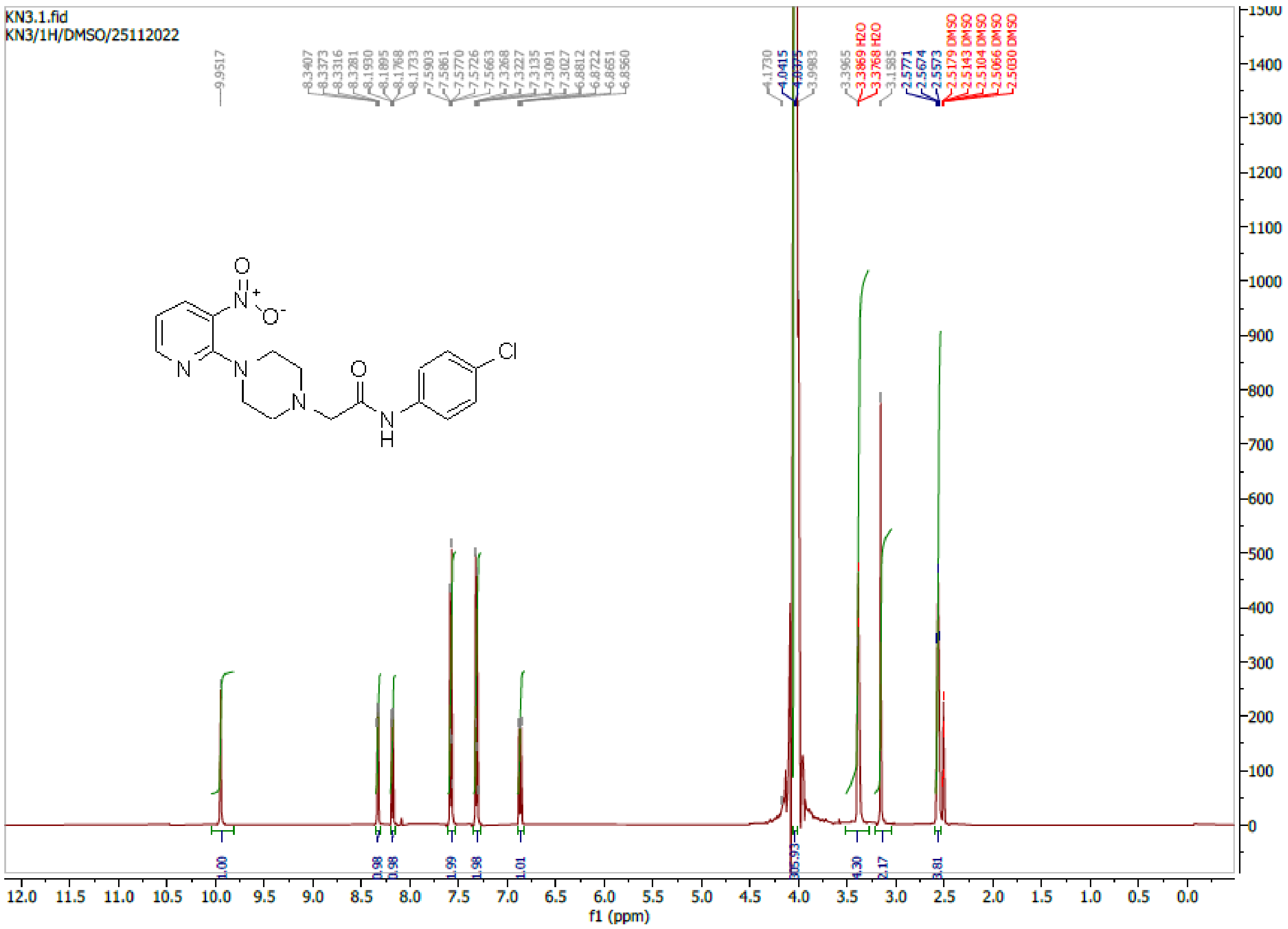

Figure S12. ^{1}HNMR of *N-(4-Chlorophenyl)-2-(4-(3-nitropyridin-2-yl)piperazin-1-yl)acetamide (**5d**)*

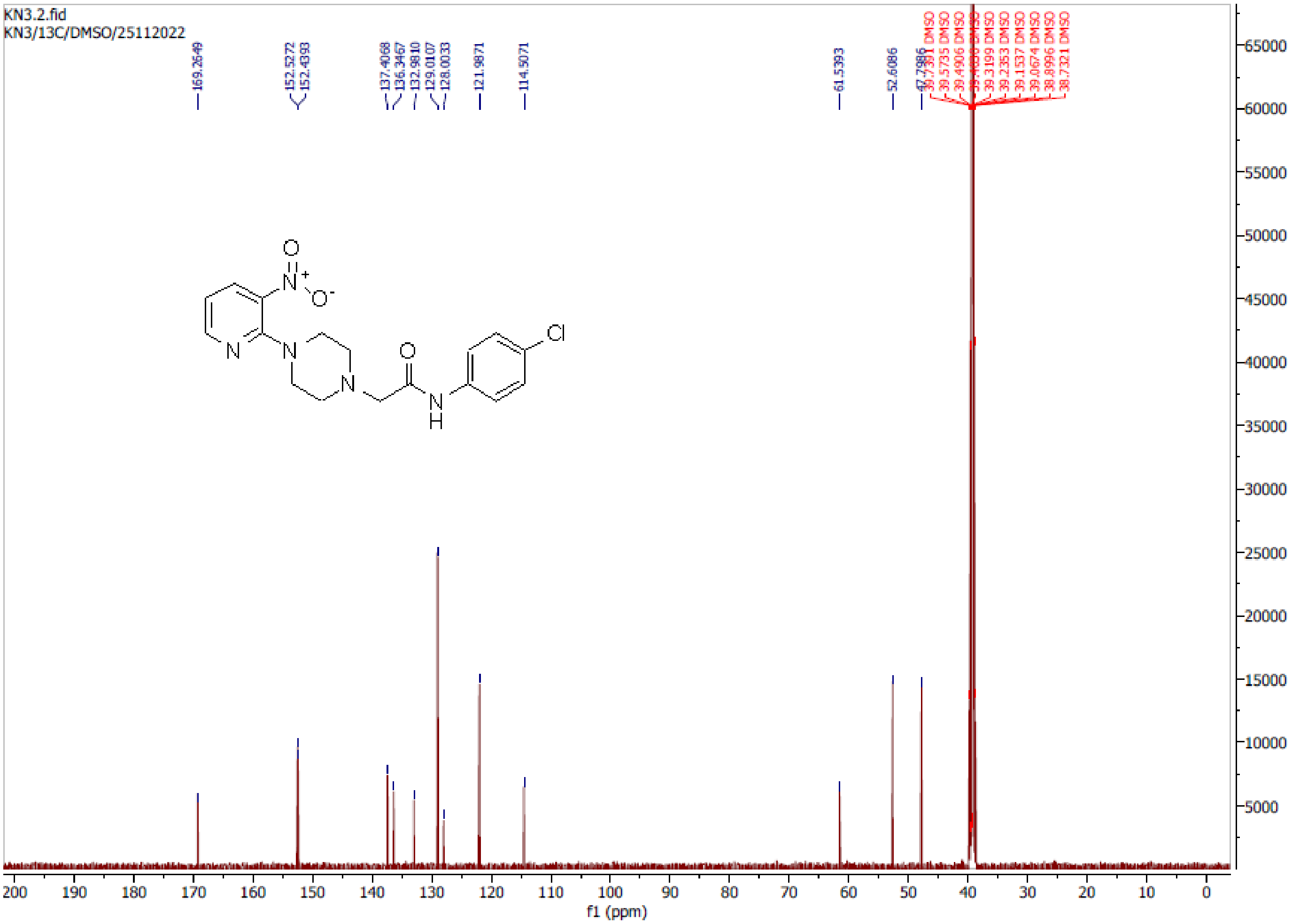

Figure S13. ^{13}CNMR of *N-(4-Chlorophenyl)-2-(4-(3-nitropyridin-2-yl)piperazin-1-yl)acetamide* (***5d***)

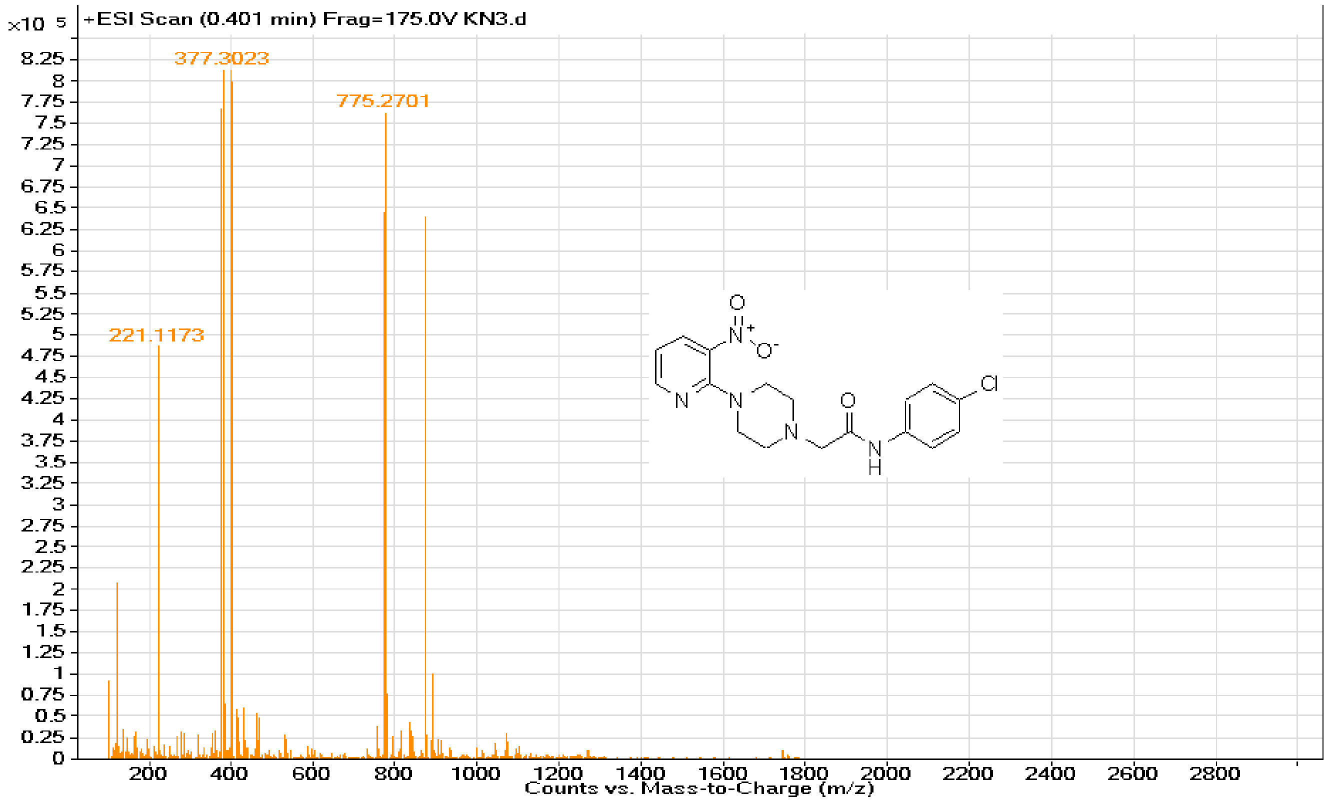

Figure S14. MS of *N-(4-Chlorophenyl)-2-(4-(3-nitropyridin-2-yl)piperazin-1-yl)acetamide* (**5d**)

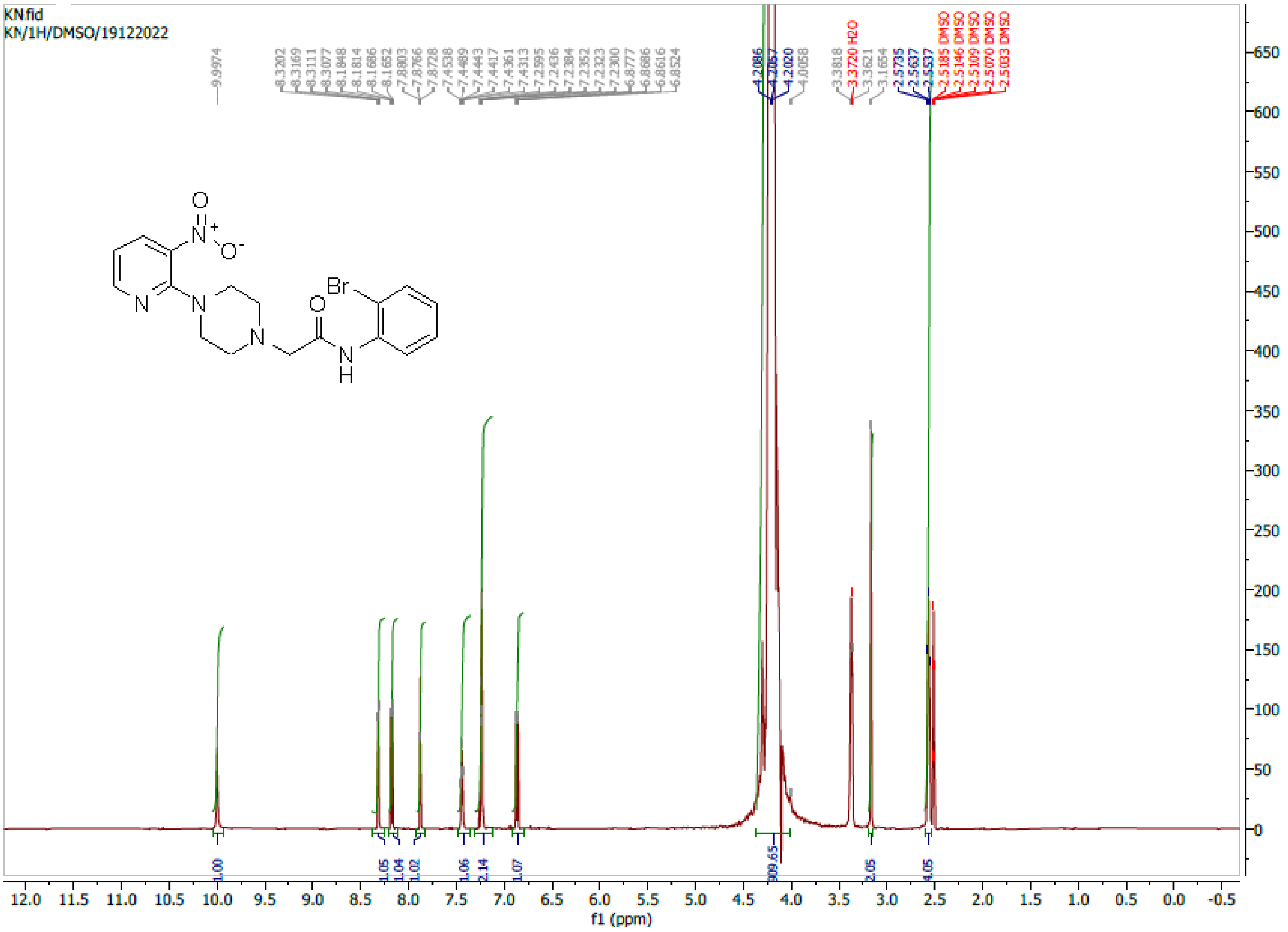

Figure S15. ^{1}HNMR of *N-(2-Bromophenyl)-2-(4-(3-nitropyridin-2-yl)piperazin-1-yl)acetamide* (***5e***)

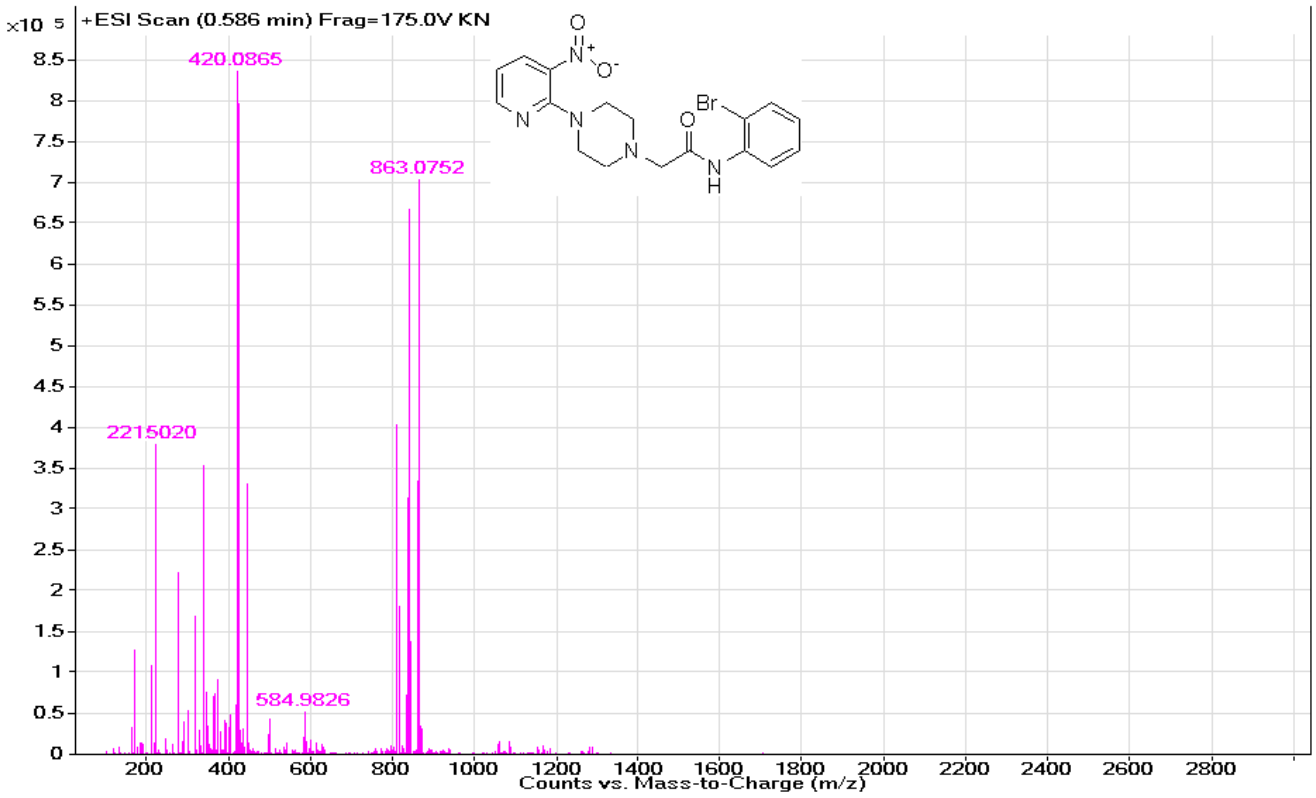

Figure S16. MS of *N-(2-Bromophenyl)-2-(4-(3-nitropyridin-2-yl)piperazin-1-yl)acetamide* (***5e***)

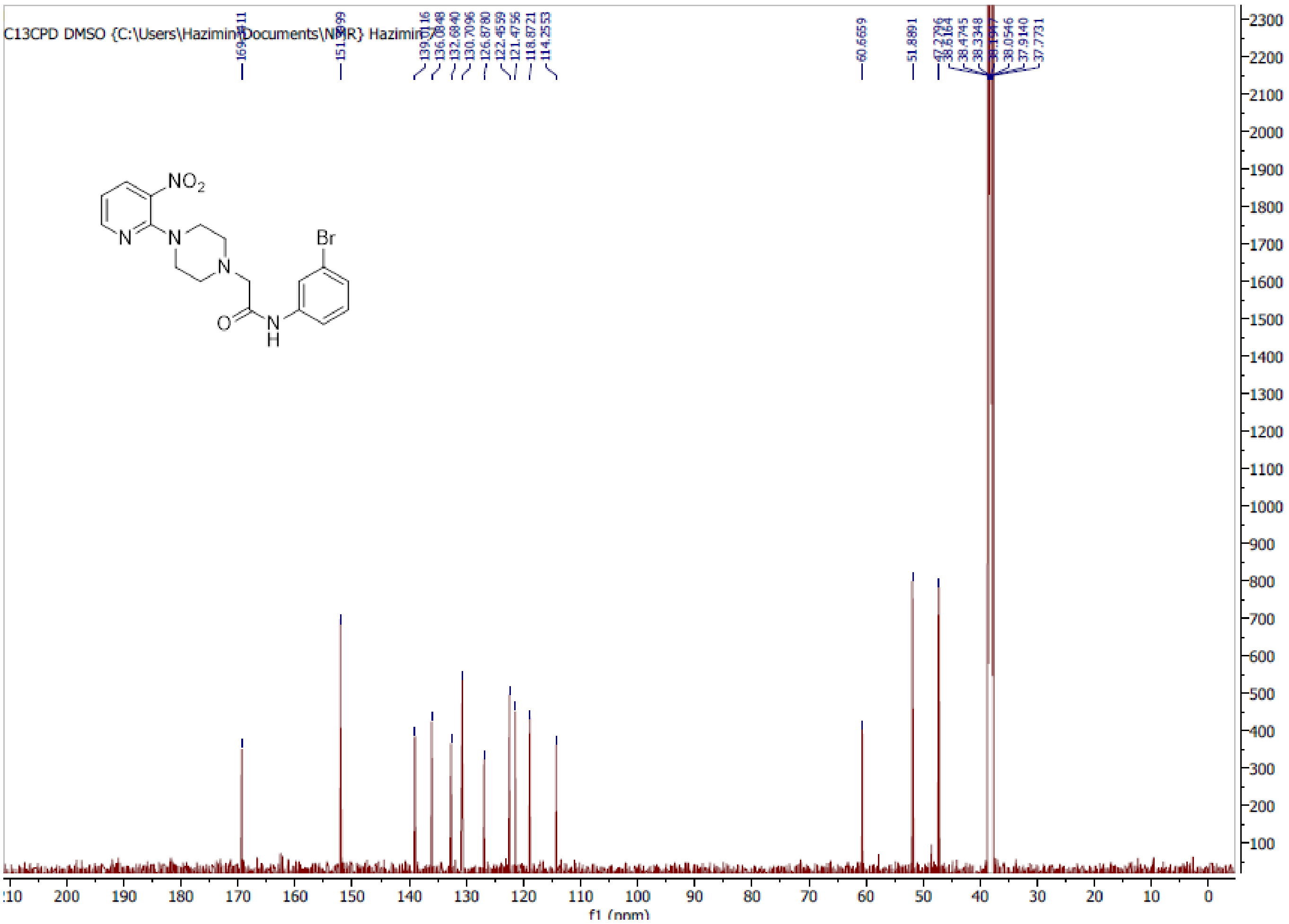

Figure S17. ^{13}CNMR of *N-(3-Bromophenyl)-2-(4-(3-nitropyridin-2-yl)piperazin-1-yl)acetamide* (***5f***)

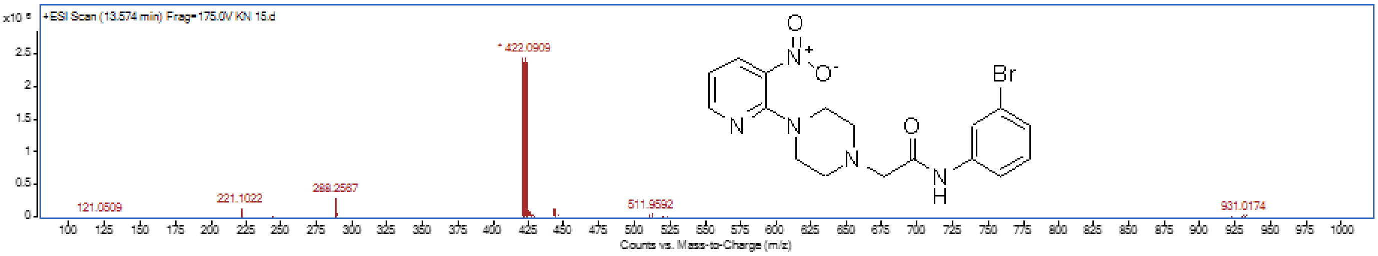

Figure S18. MS of *N-(3-Bromophenyl)-2-(4-(3-nitropyridin-2-yl)piperazin-1-yl)acetamide* (***5f***)

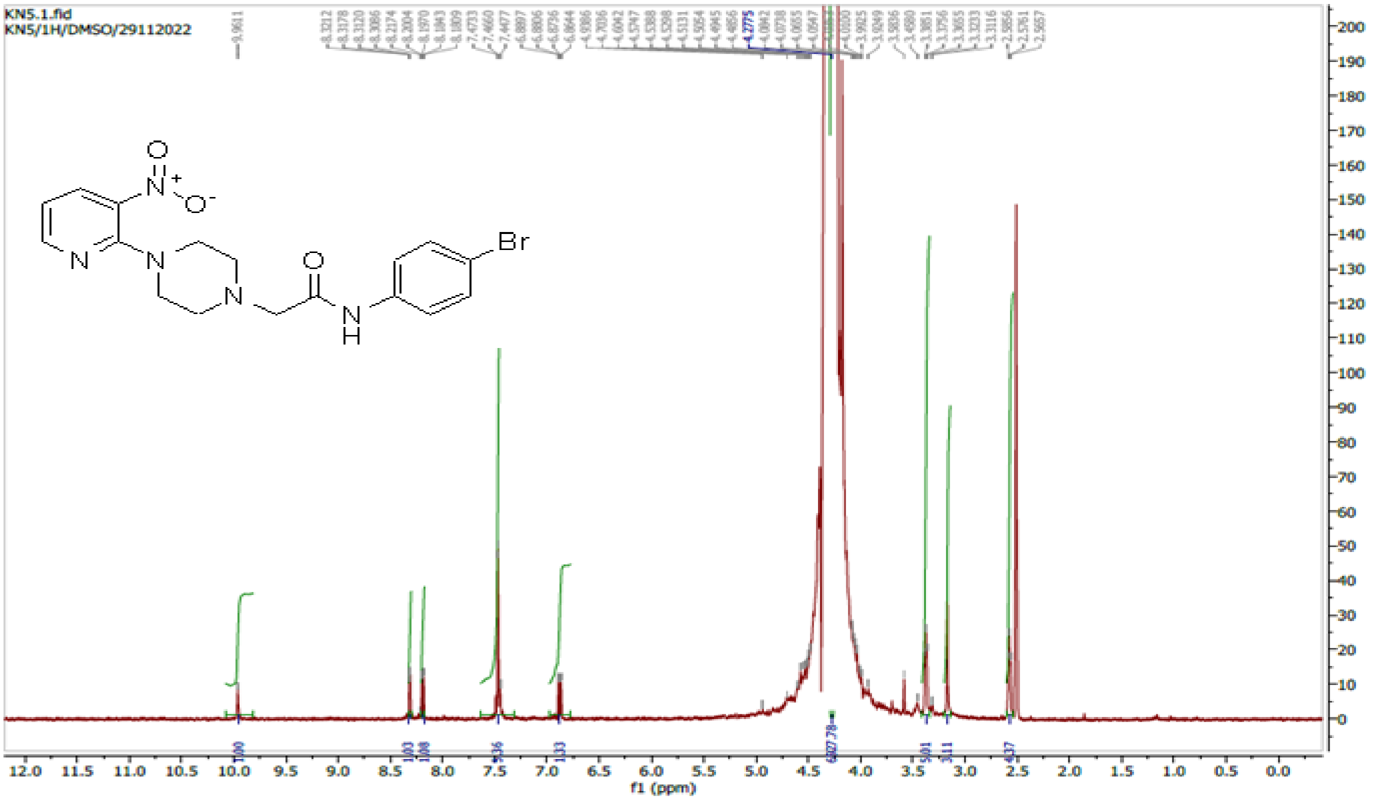

Figure S19. ^{1}HNMR of *N-(4-Bromophenyl)-2-(4-(3-nitropyridin-2-yl)piperazin-1-yl)acetamide* (**5g**)

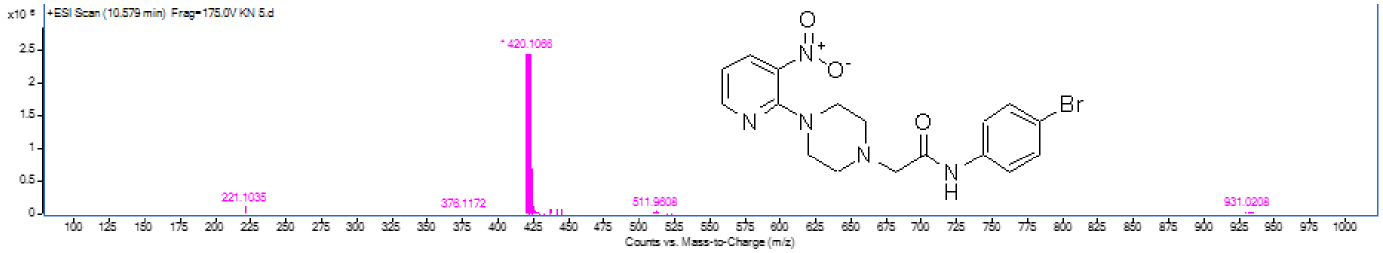

Figure S20. MS of *N-(4-Bromophenyl)-2-(4-(3-nitropyridin-2-yl)piperazin-1-yl)acetamide* (**5g**)

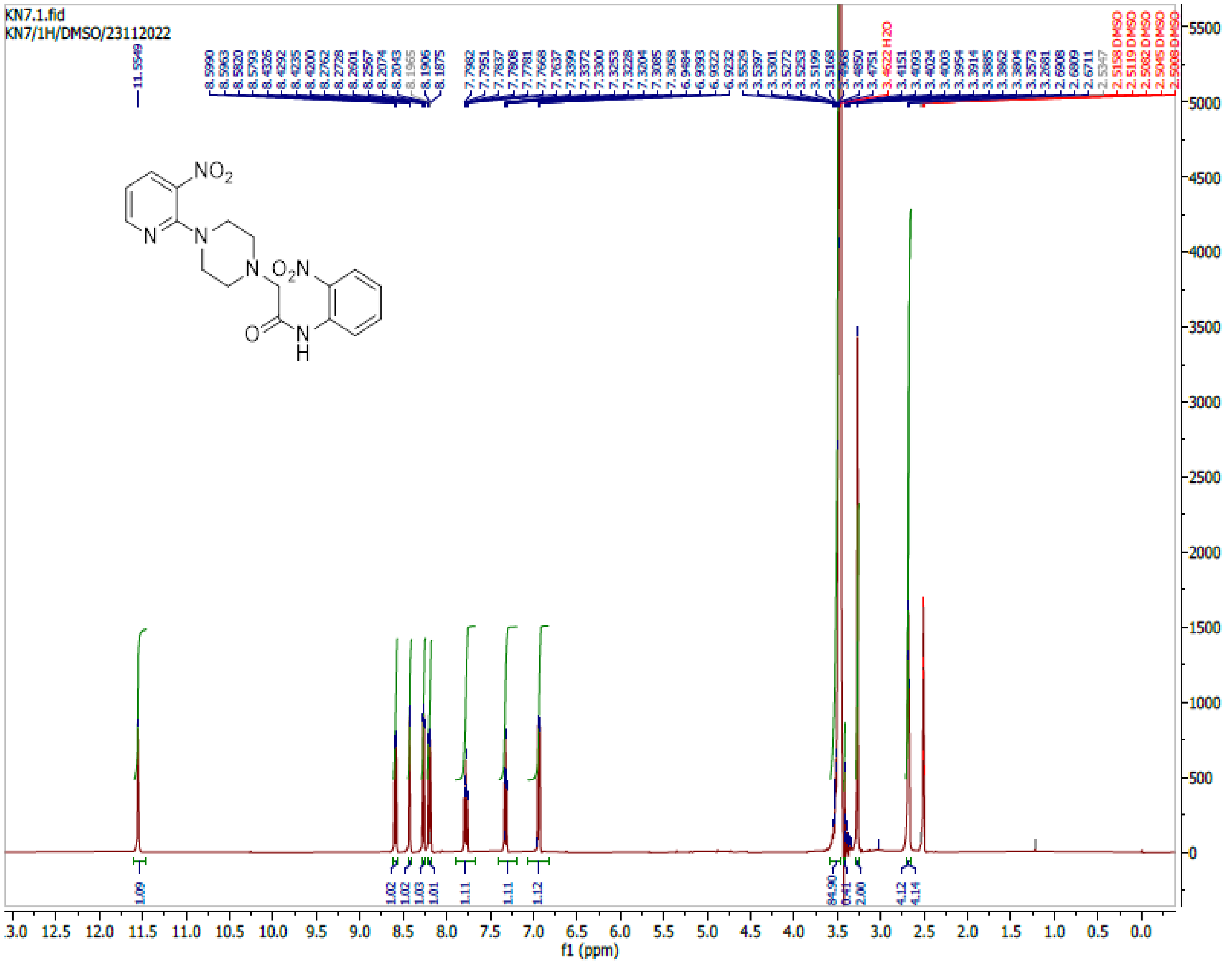

Figure S21. ^{1}HNMR of *N-(2-Nitrophenyl)-2-(4-(3-nitropyridin-2-yl)piperazin-1-yl)acetamide (**5h**)*

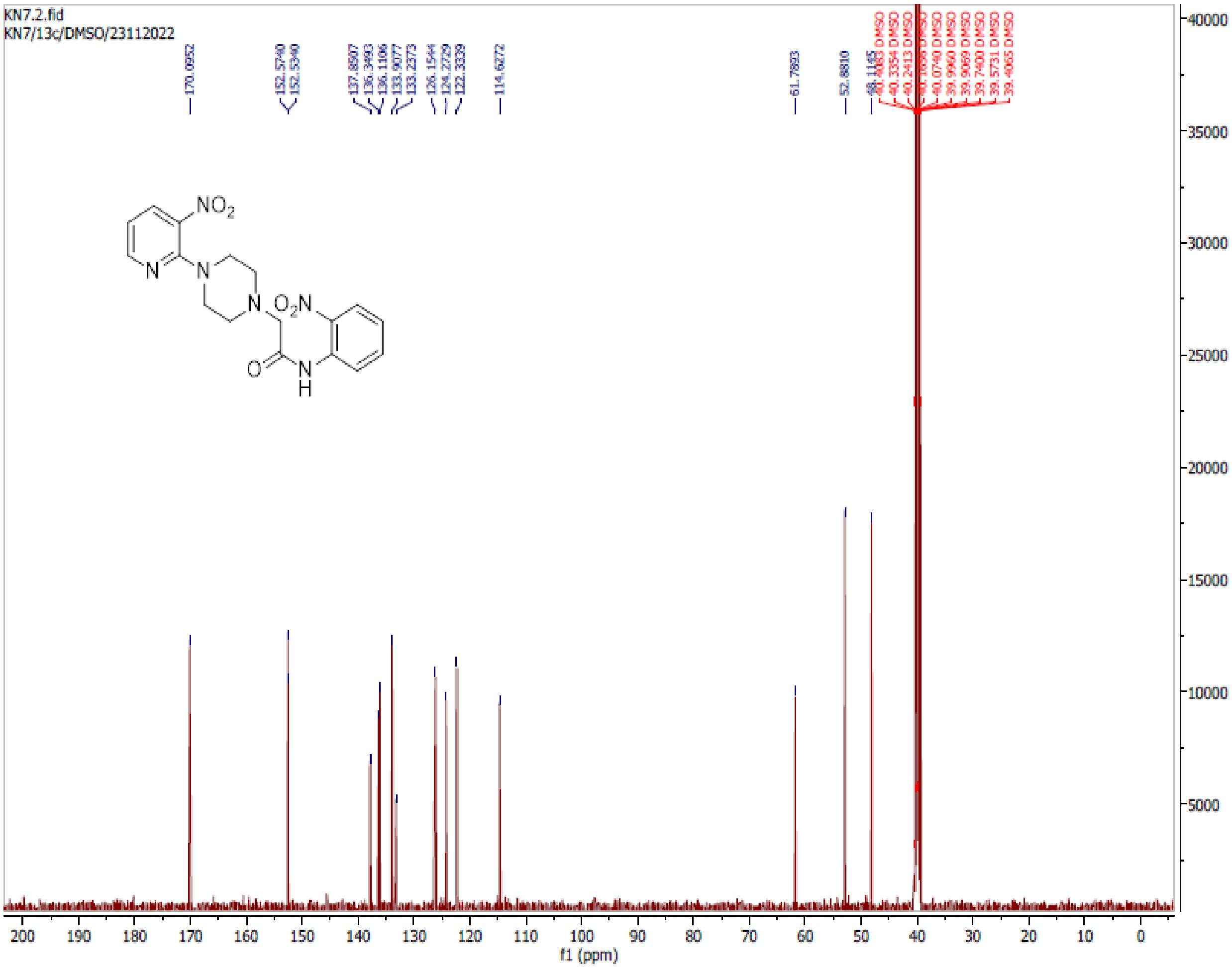

Figure S22. ^{13}CNMR of *N-(2-Nitrophenyl)-2-(4-(3-nitropyridin-2-yl)piperazin-1-yl)acetamide* (***5h***)

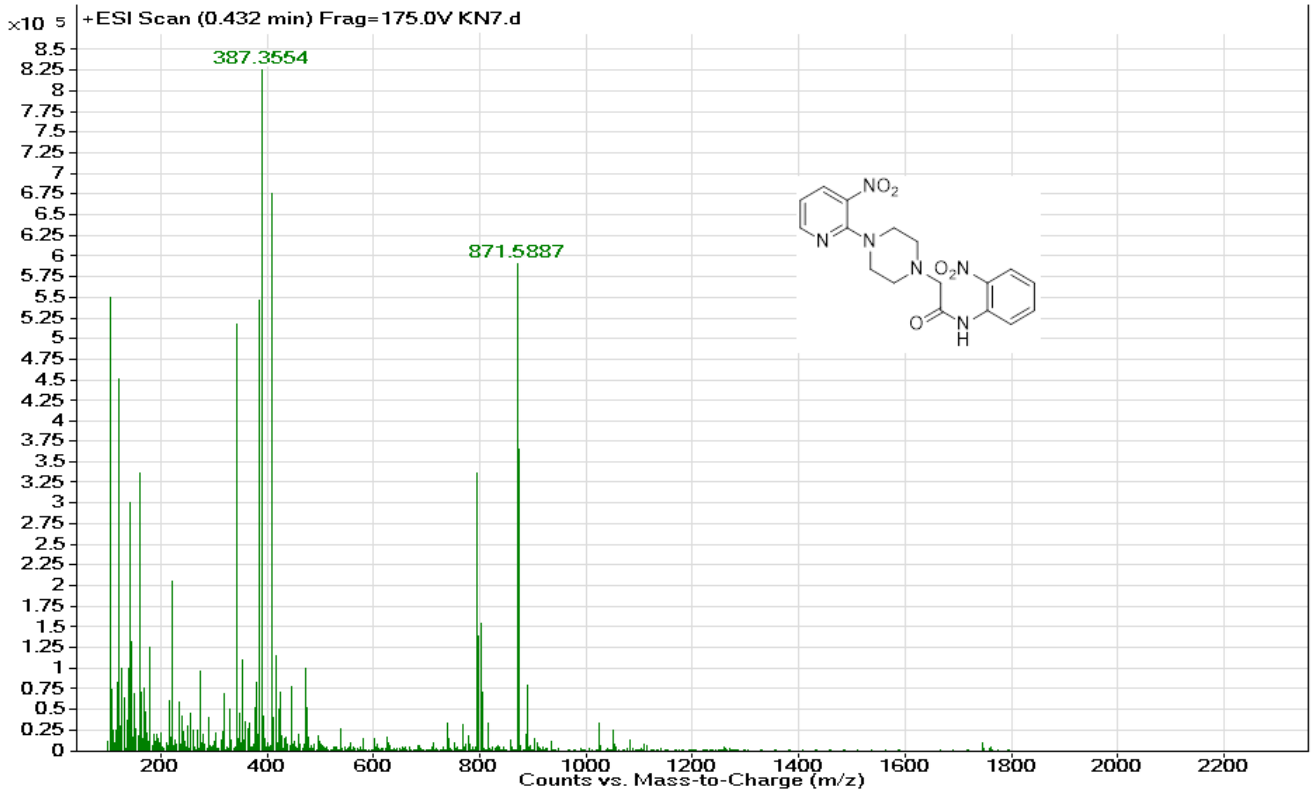

Figure S23. MS of *N-(2-Nitrophenyl)-2-(4-(3-nitropyridin-2-yl)piperazin-1-yl)acetamide (**5h**)*

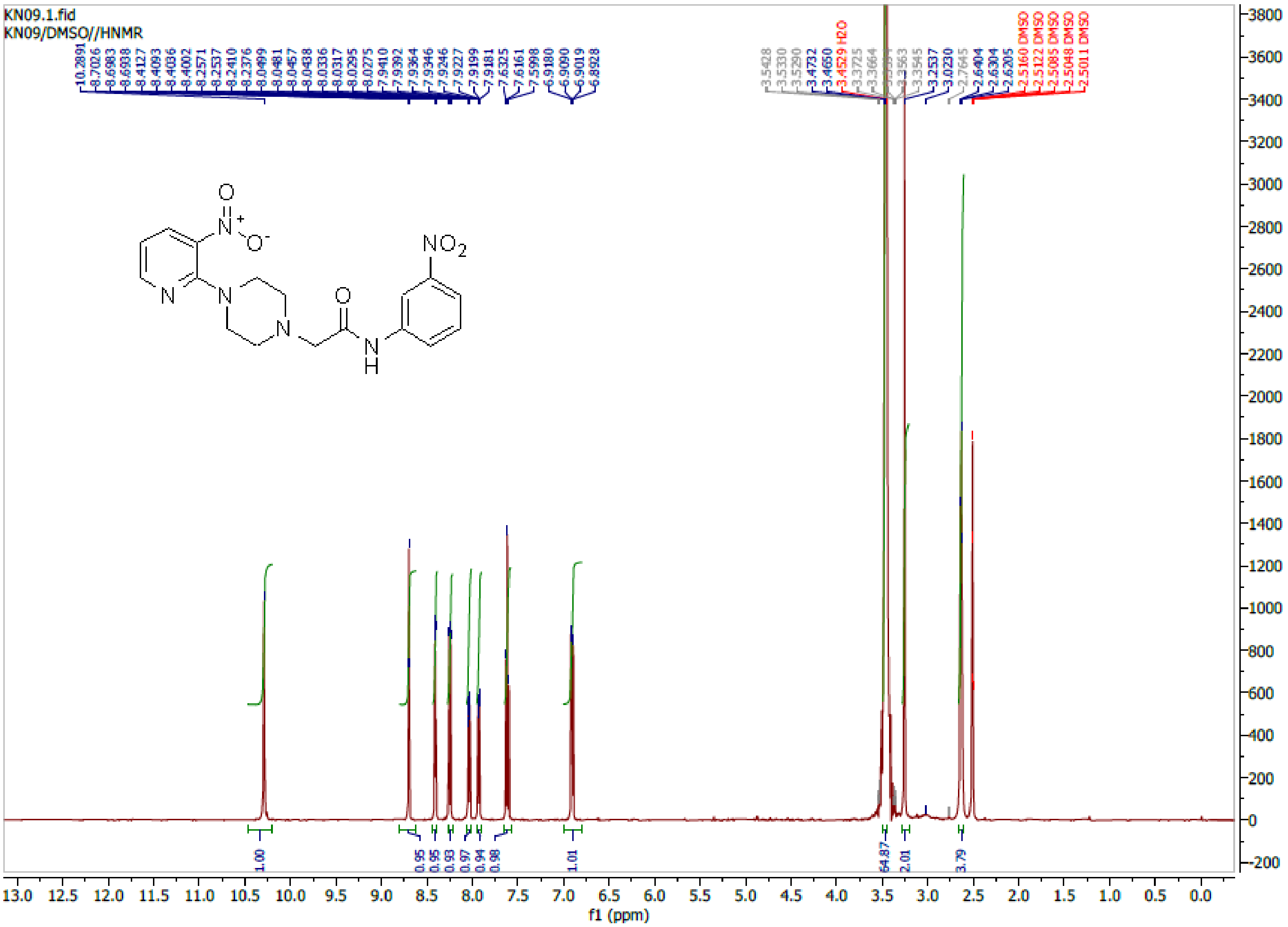

Figure S24. ¹HNMR of *N-(3-Nitrophenyl)-2-(4-(3-nitropyridin-2-yl)piperazin-1-yl)acetamide (**5i**)*

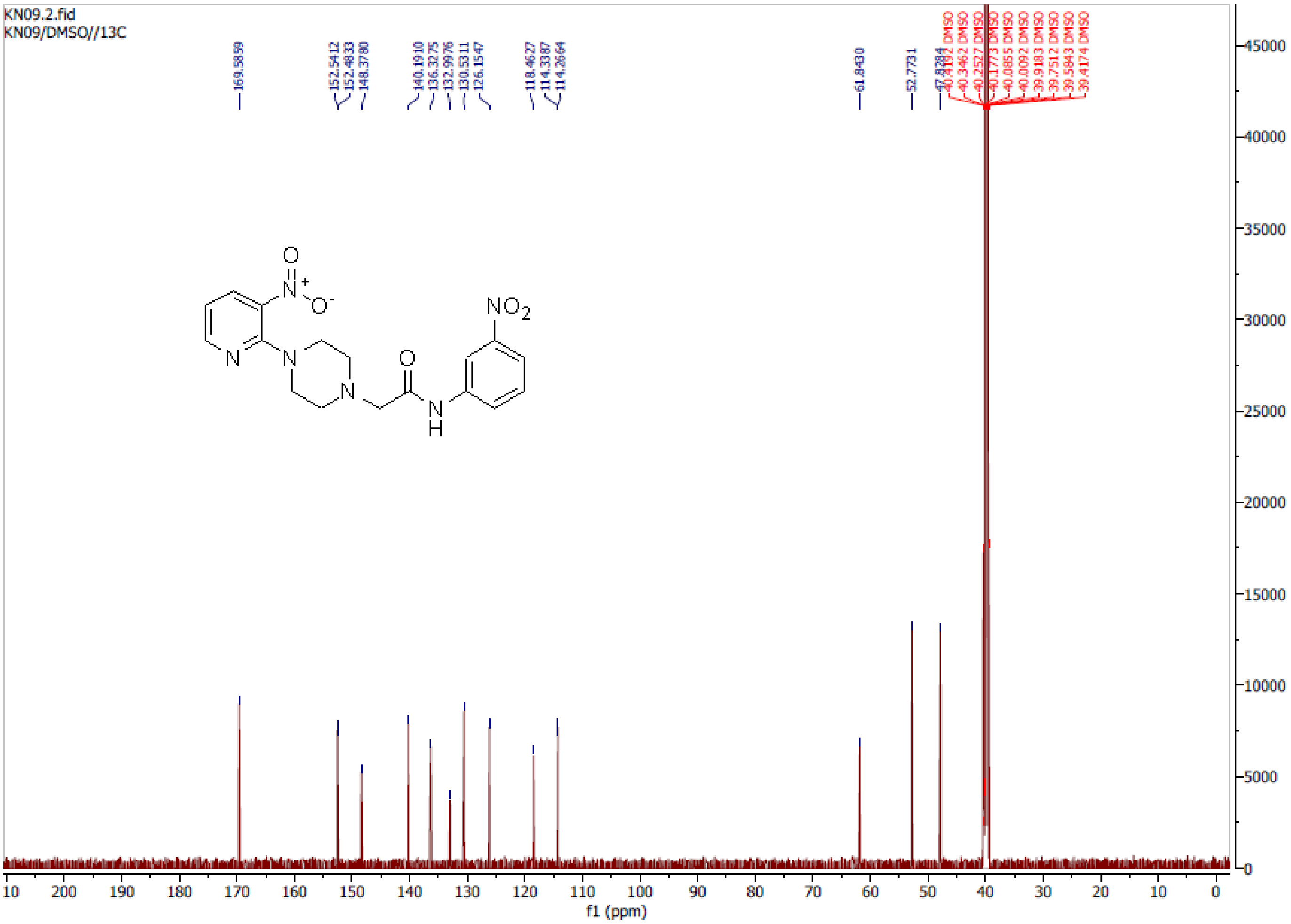

Figure S25. ^{13}CNMR of *N-(3-Nitrophenyl)-2-(4-(3-nitropyridin-2-yl)piperazin-1-yl)acetamide* (***5i***)

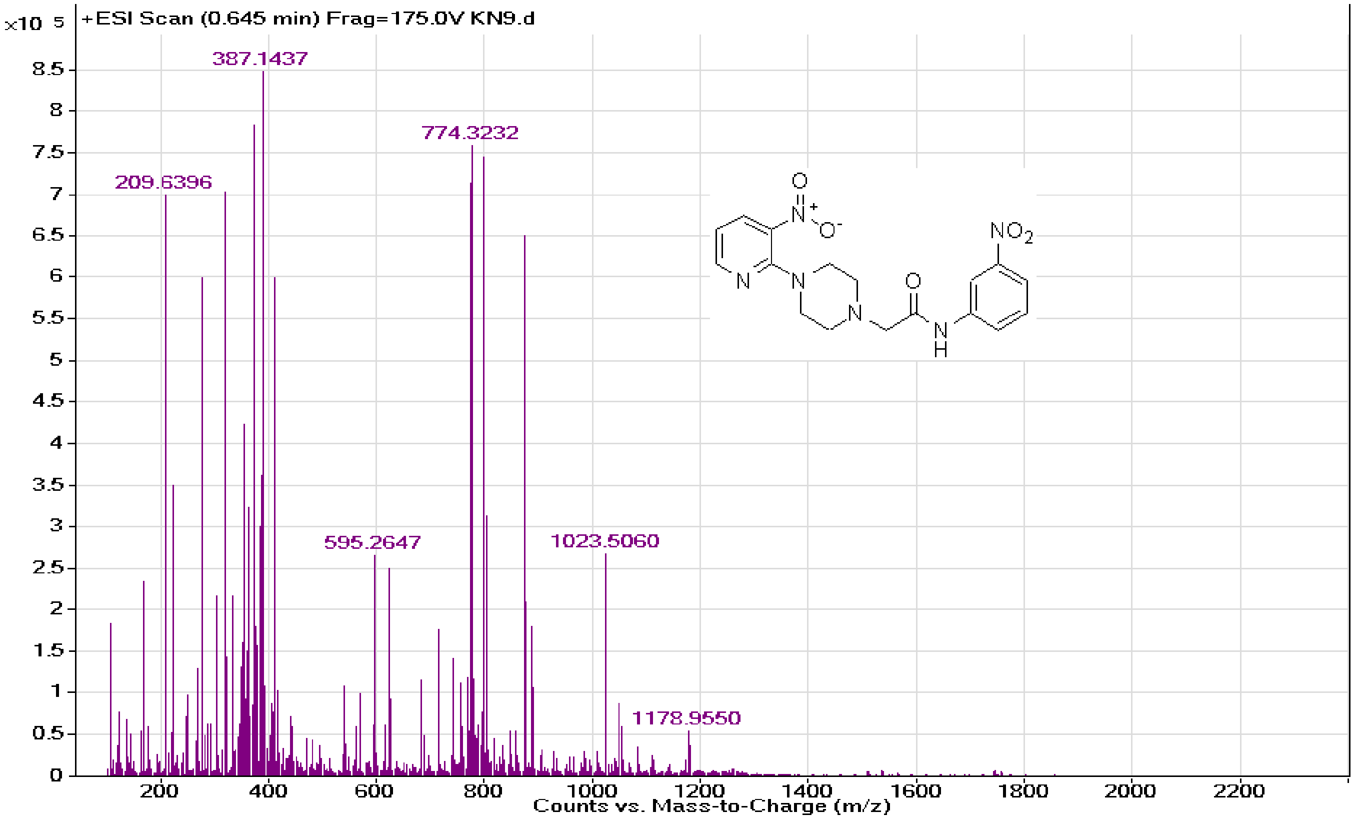

Figure S26. MS of *N-(3-Nitrophenyl)-2-(4-(3-nitropyridin-2-yl)piperazin-1-yl)acetamide (**5i**)*

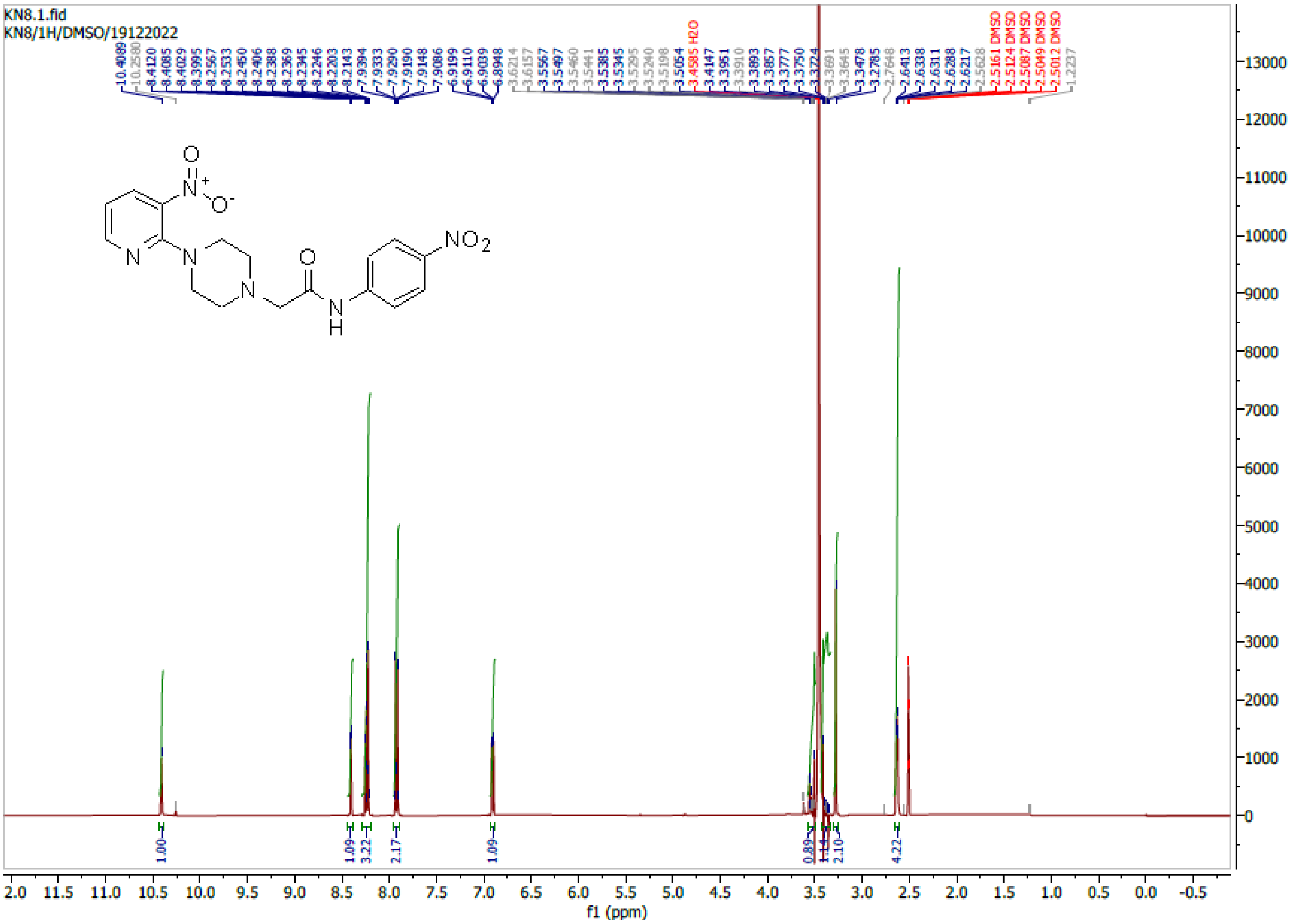

Figure S27. ^{1}HNMR of *N-(4-Nitrophenyl)-2-(4-(3-nitropyridin-2-yl)piperazin-1-yl)acetamide* (**5j**)

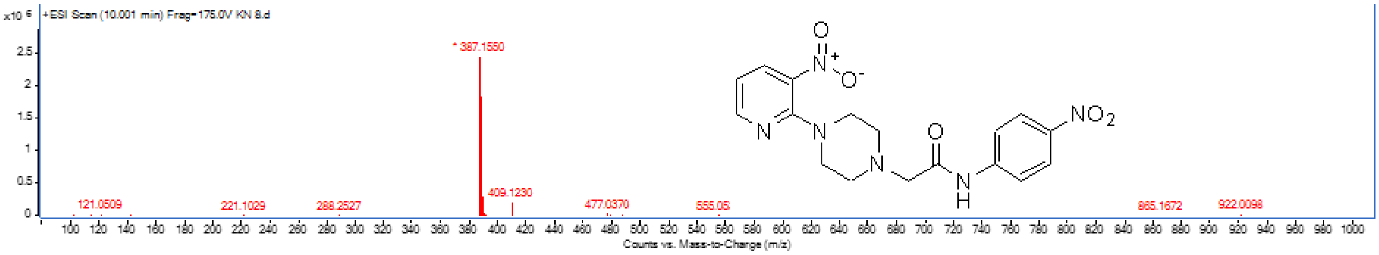

Figure S28. MS of *N-(4-Nitrophenyl)-2-(4-(3-nitropyridin-2-yl)piperazin-1-yl)acetamide* (**5j**)

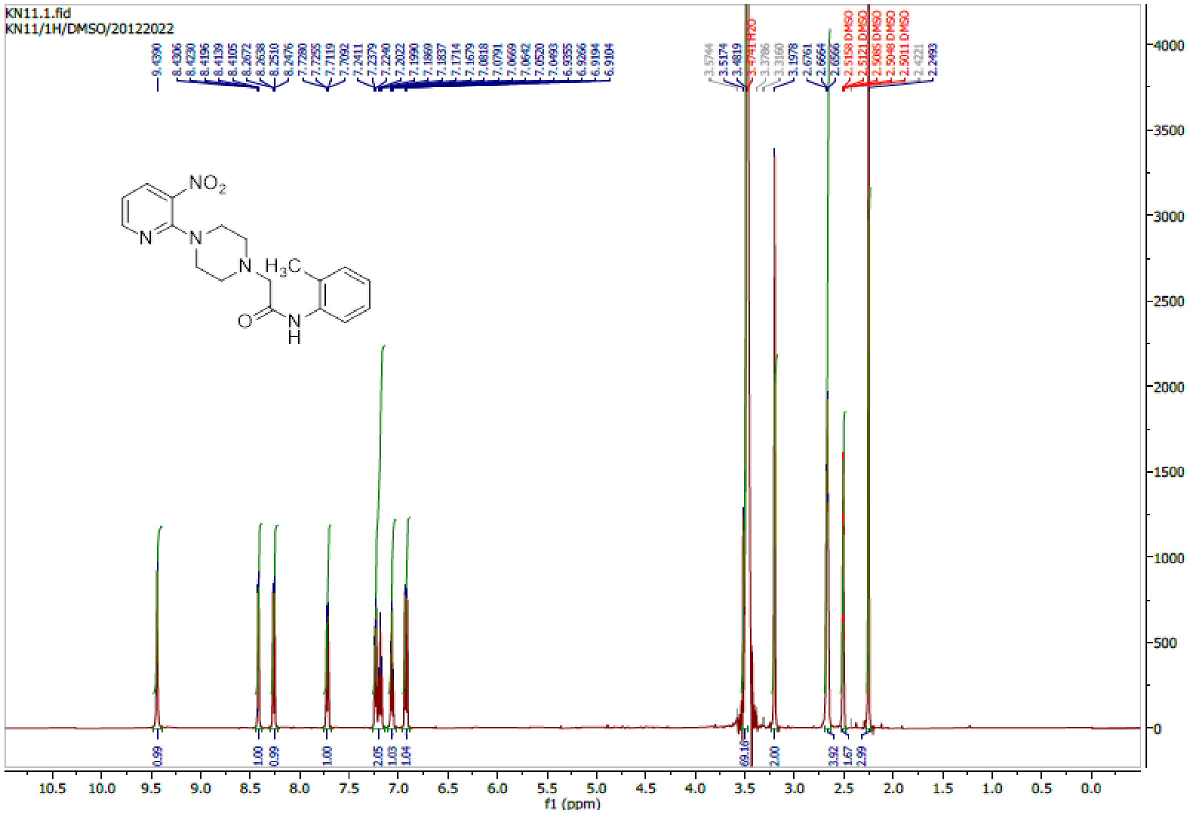

Figure S29. ^{1}HNMR of *2-(4-(3-Nitropyridin-2-yl)piperazin-1-yl)-N-(o-tolyl)acetamide (**5k**)*

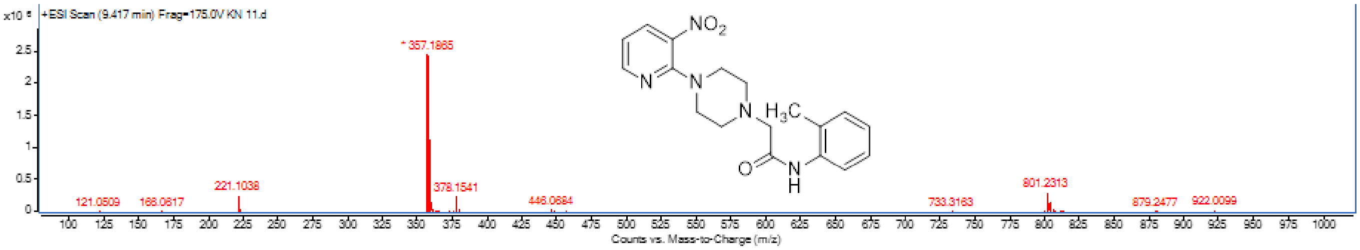

Figure S30. MS of *2-(4-(3-Nitropyridin-2-yl)piperazin-1-yl)-N-(o-tolyl)acetamide* (***5k***)

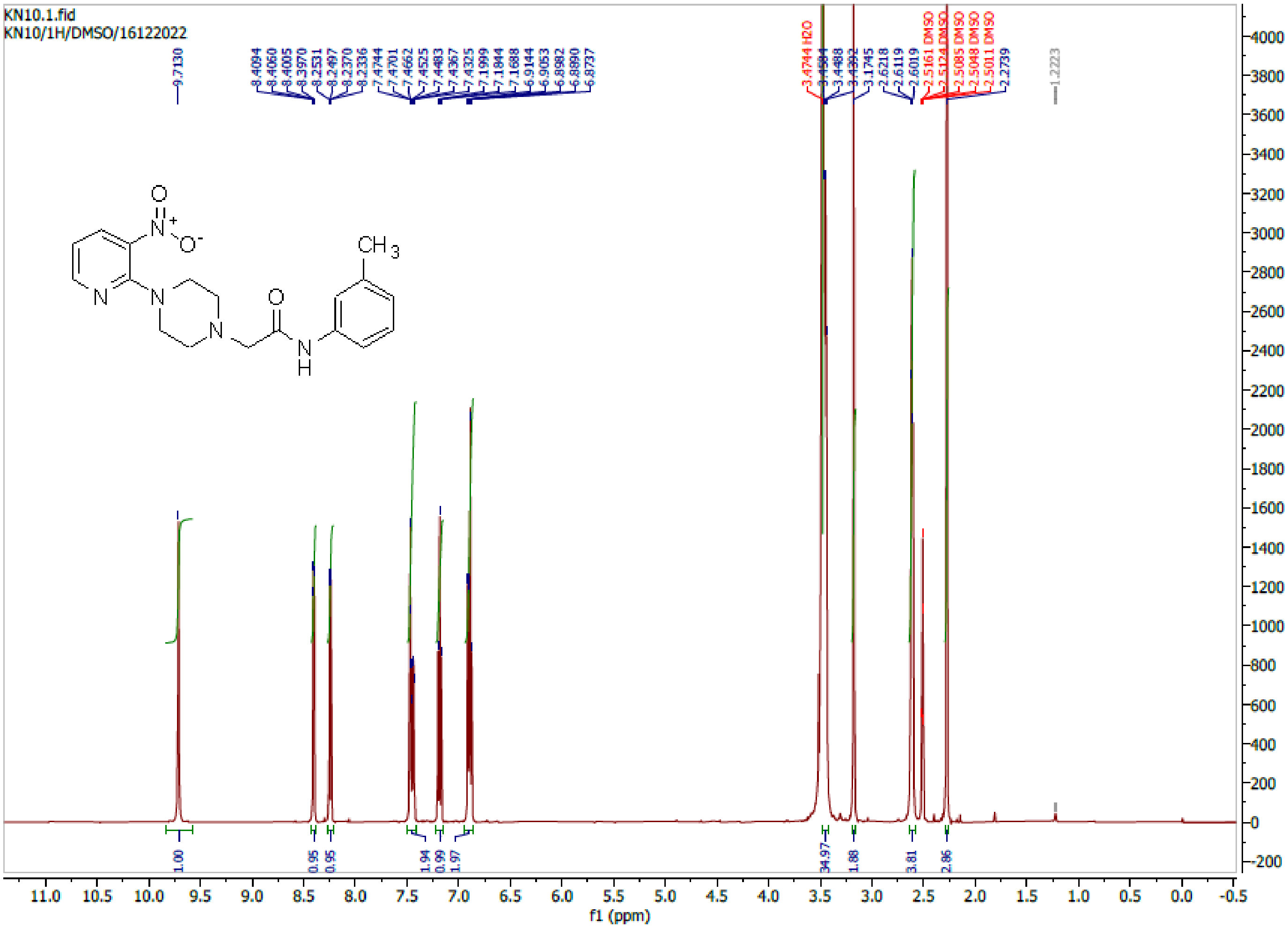

Figure S31. ^{1}HNMR of *2-(4-(3-Nitropyridin-2-yl)piperazin-1-yl)-N-(m-tolyl)acetamide (**5l**)*

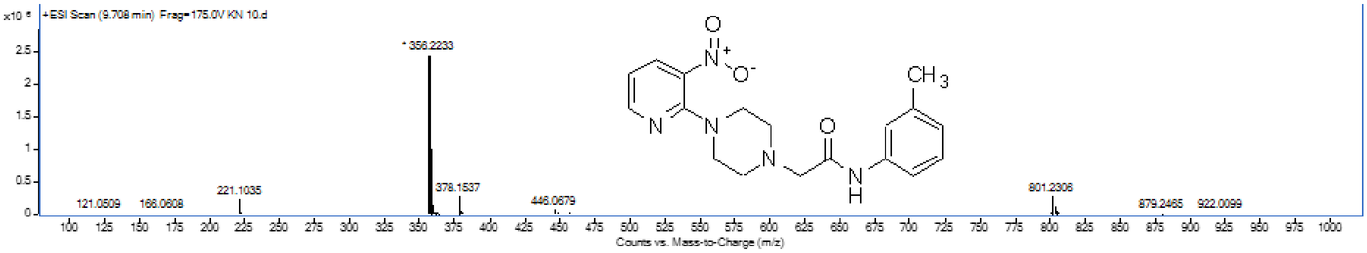

Figure S32. MS of *2-(4-(3-Nitropyridin-2-yl)piperazin-1-yl)-N-(m-tolyl)acetamide (**5l**)*

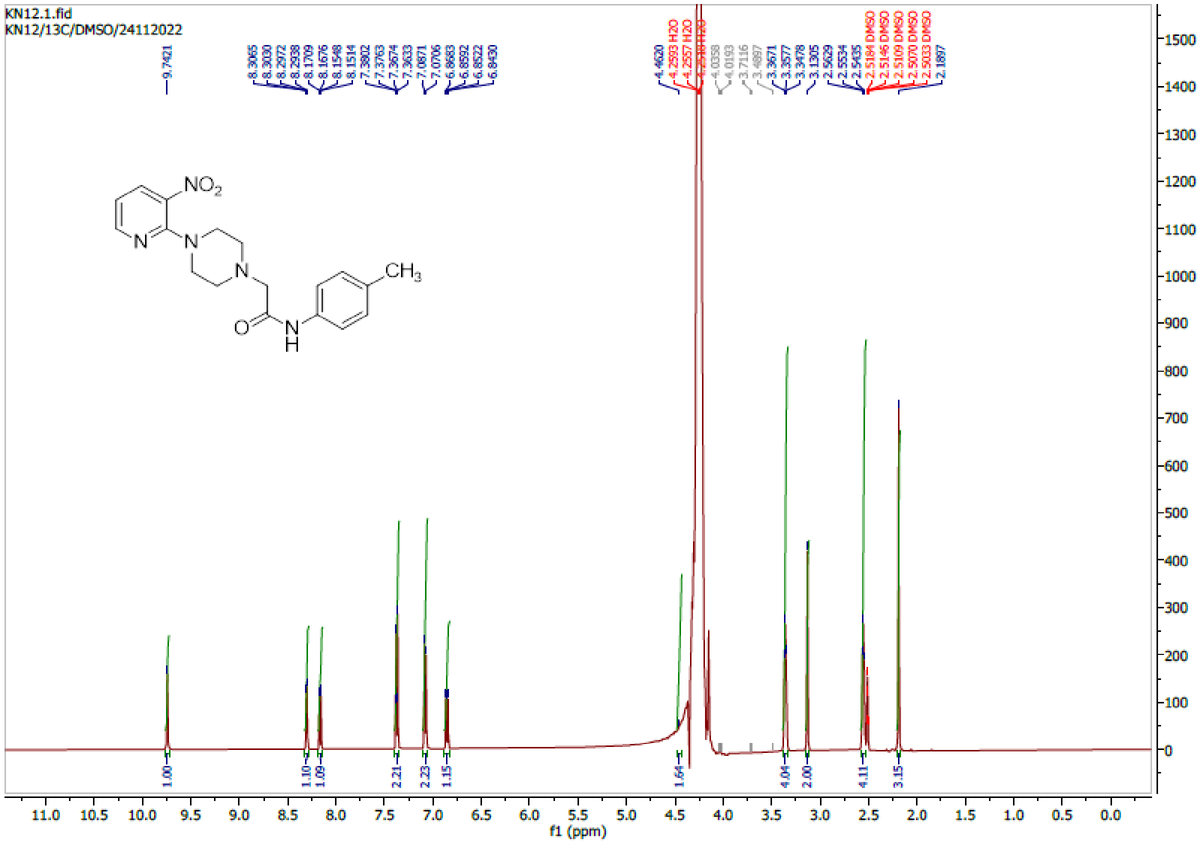

Figure S33. ^{1}HNMR of *2-(4-(3-Nitropyridin-2-yl)piperazin-1-yl)-N-(p-tolyl)acetamide* ***(5m)***

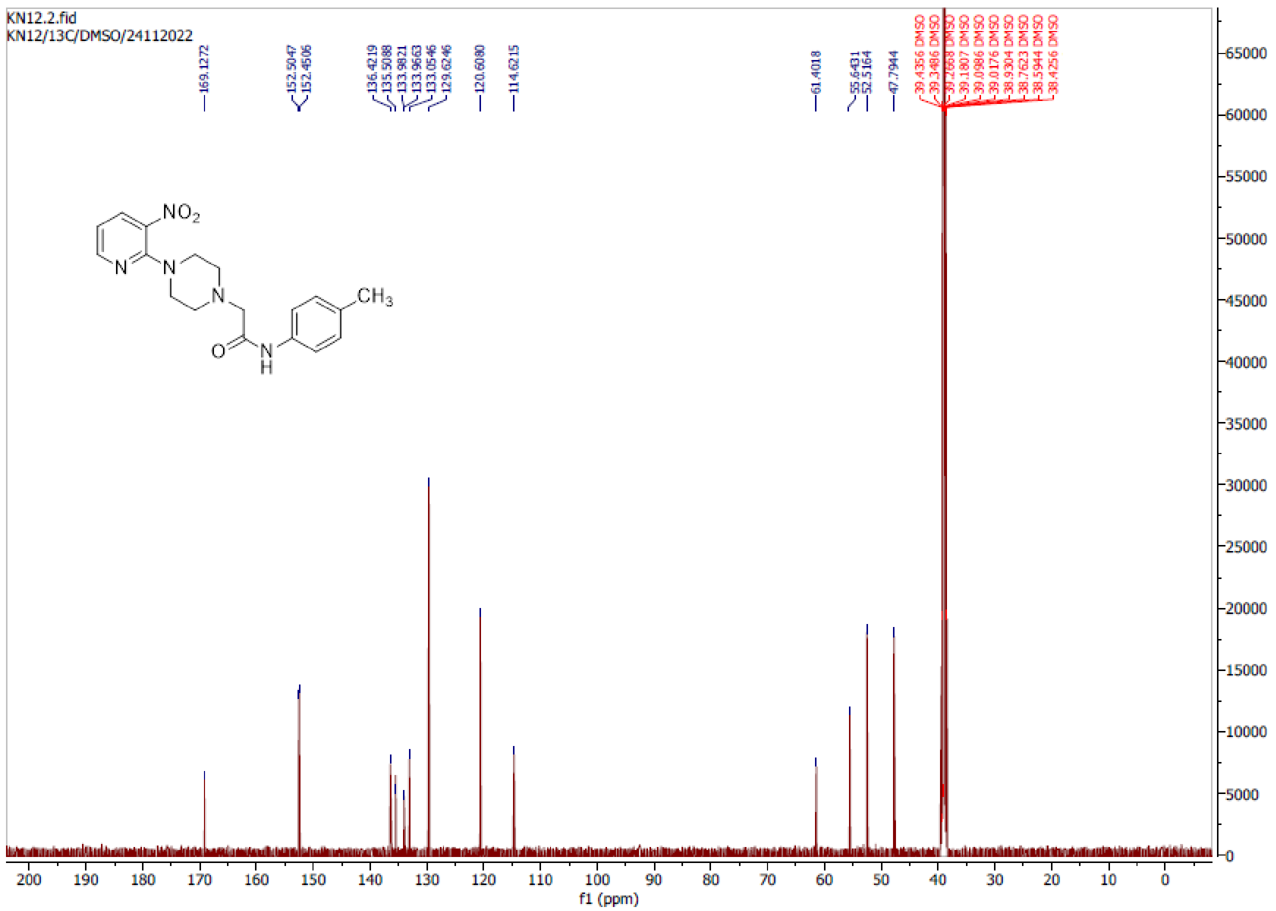

Figure S34. 13CNMR of *2-(4-(3-Nitropyridin-2-yl)piperazin-1-yl)-N-(p-tolyl)acetamide (**5m**)*

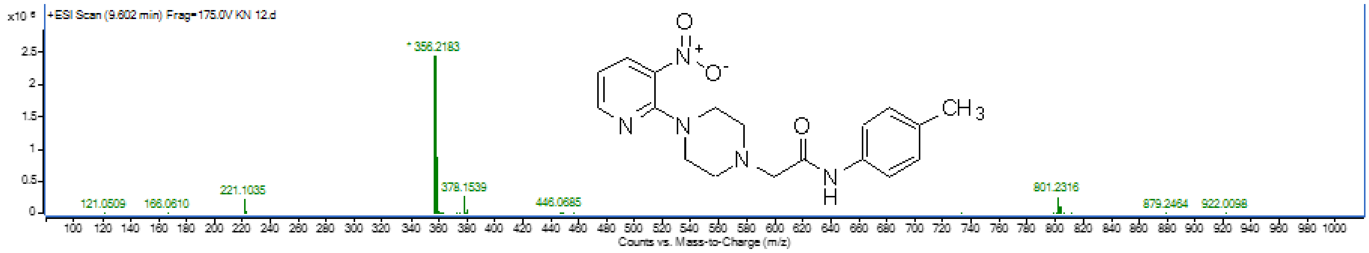

Figure S35. MS of *2-(4-(3-Nitropyridin-2-yl)piperazin-1-yl)-N-(p-tolyl)acetamide* (***5m***)

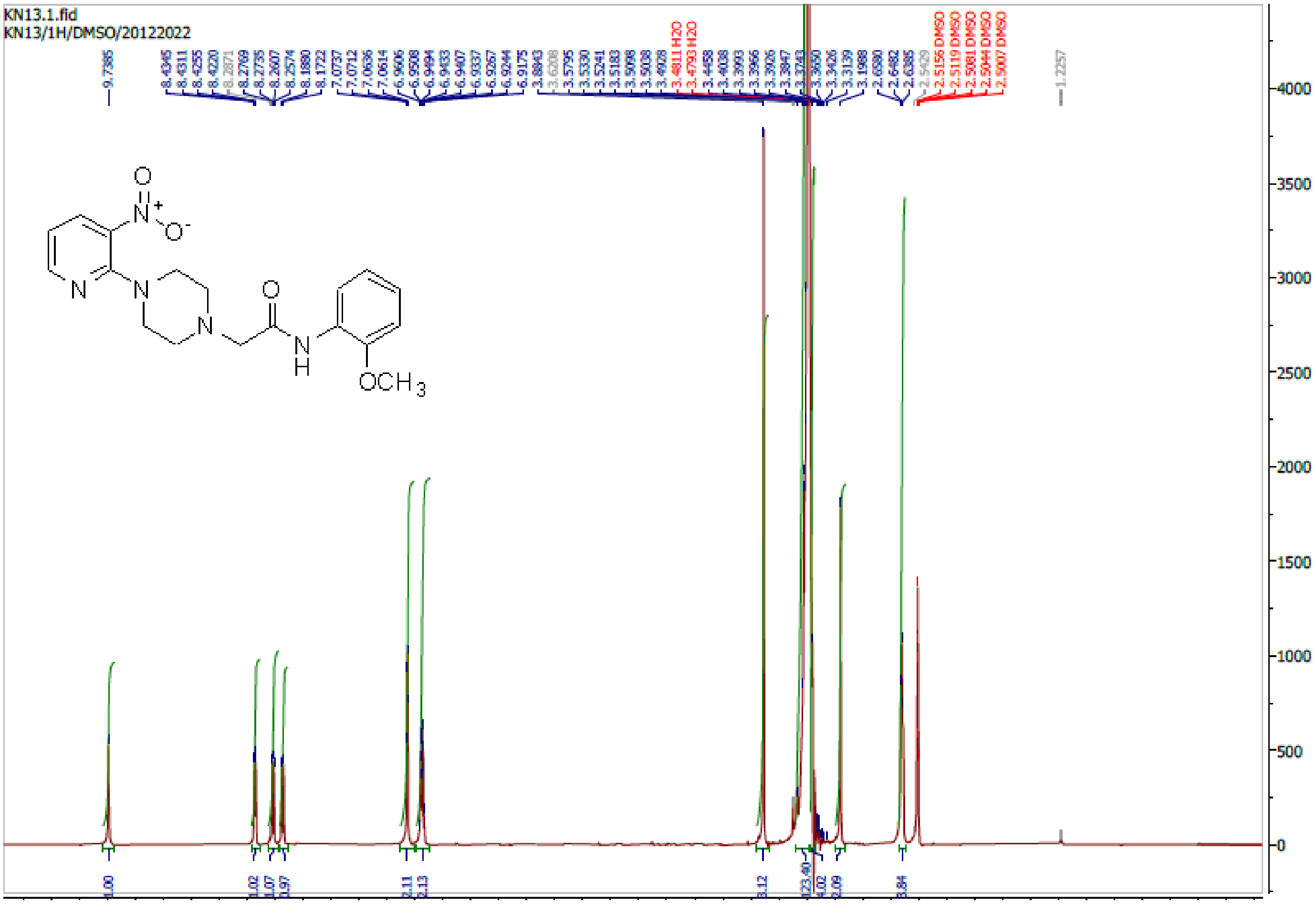

Figure S36. ¹HNMR of *N-(2-Methoxyphenyl)-2-(4-(3-nitropyridin-2-yl)piperazin-1-yl)acetamide* (***5n***)

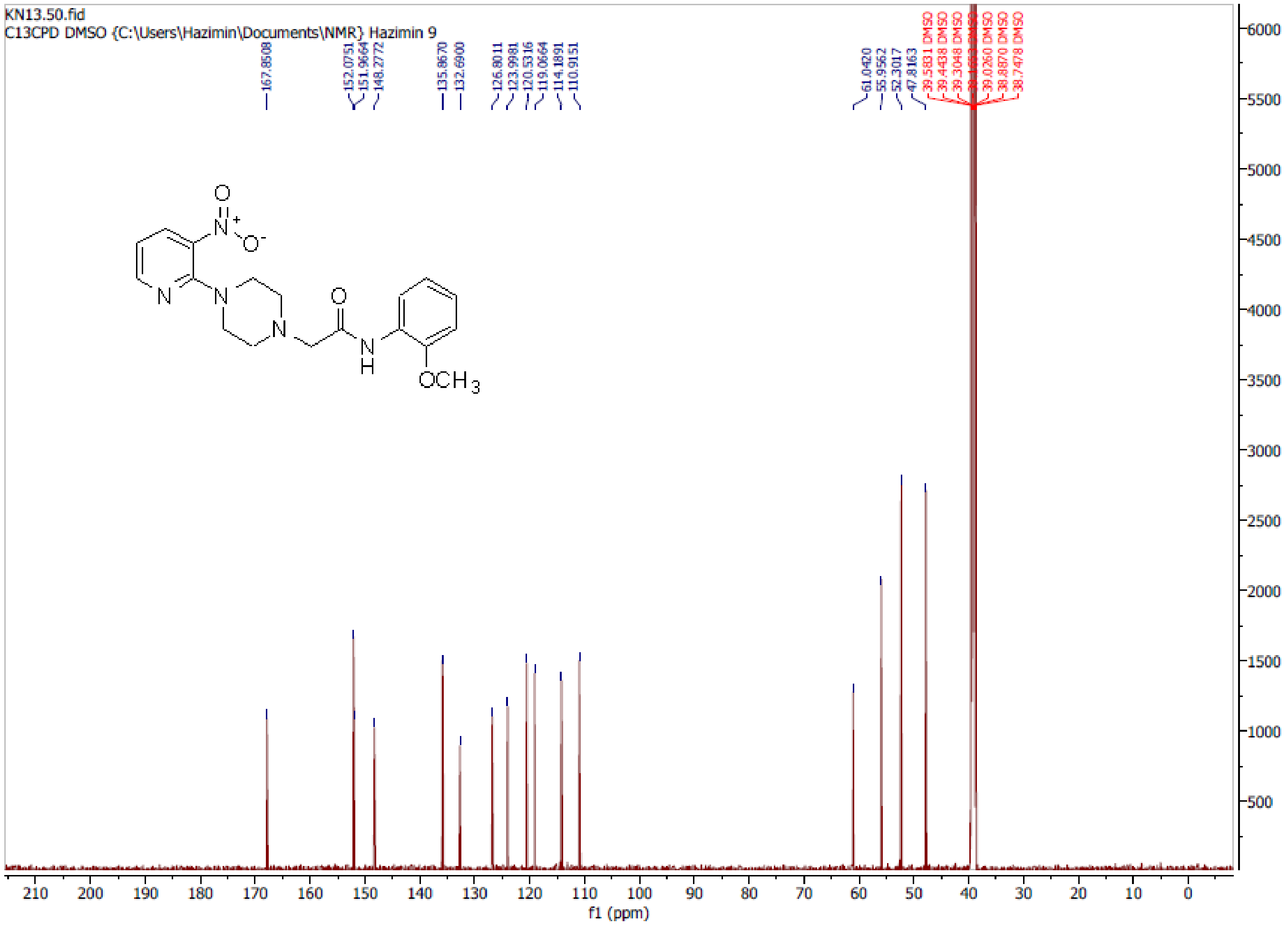

Figure S37. ^{13}CNMR of *N-(2-Methoxyphenyl)-2-(4-(3-nitropyridin-2-yl)piperazin-1-yl)acetamide* (**5n**)

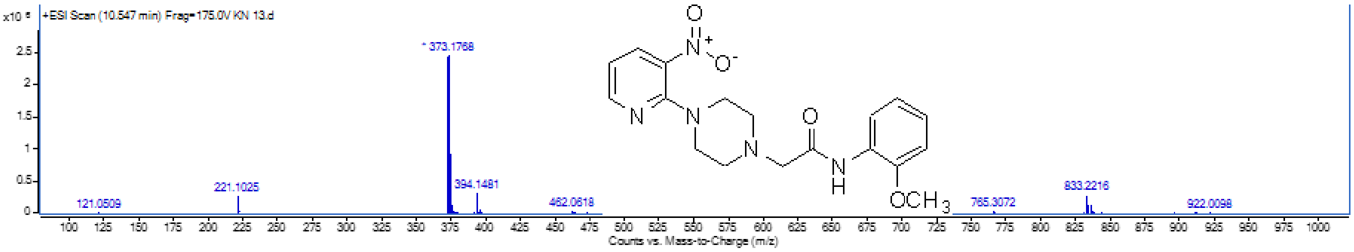

Figure S38. MS of *N-(2-Methoxyphenyl)-2-(4-(3-nitropyridin-2-yl)piperazin-1-yl)acetamide* (**5n**)

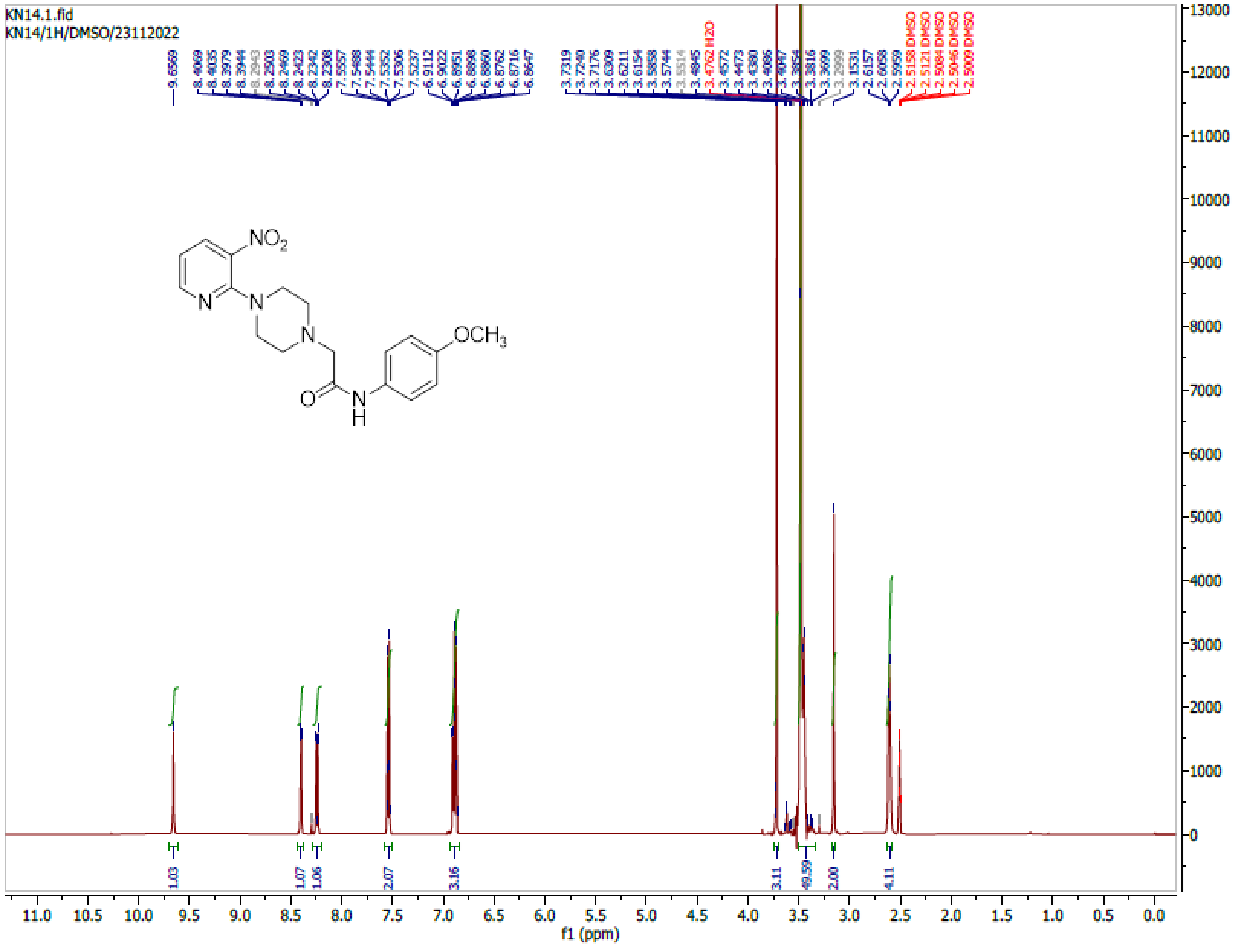

Figure S39. ^{1}HNMR of *N-(4-Methoxyphenyl)-2-(4-(3-nitropyridin-2-yl)piperazin-1-yl)acetamide* (**5o**)

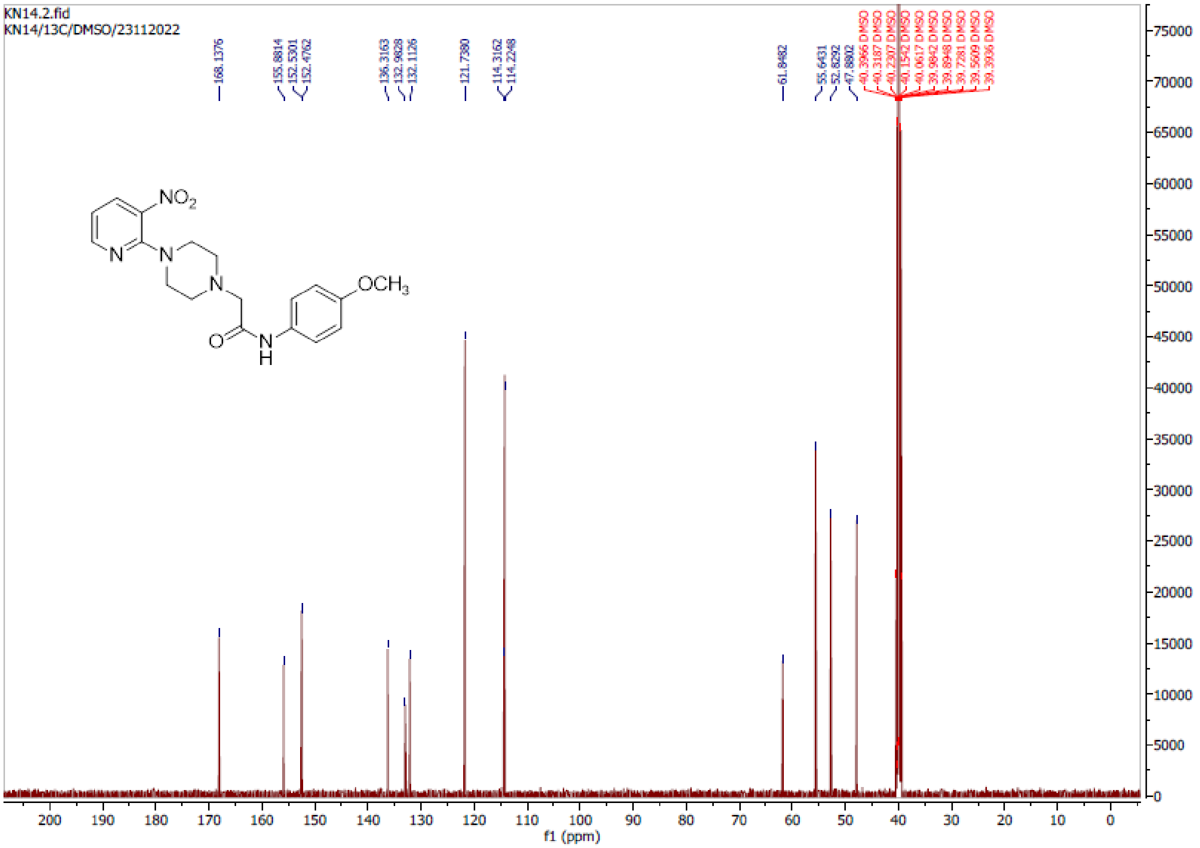

Figure S40. ^{13}CNMR of *N-(4-Methoxyphenyl)-2-(4-(3-nitropyridin-2-yl)piperazin-1-yl)acetamide (**5o**)*

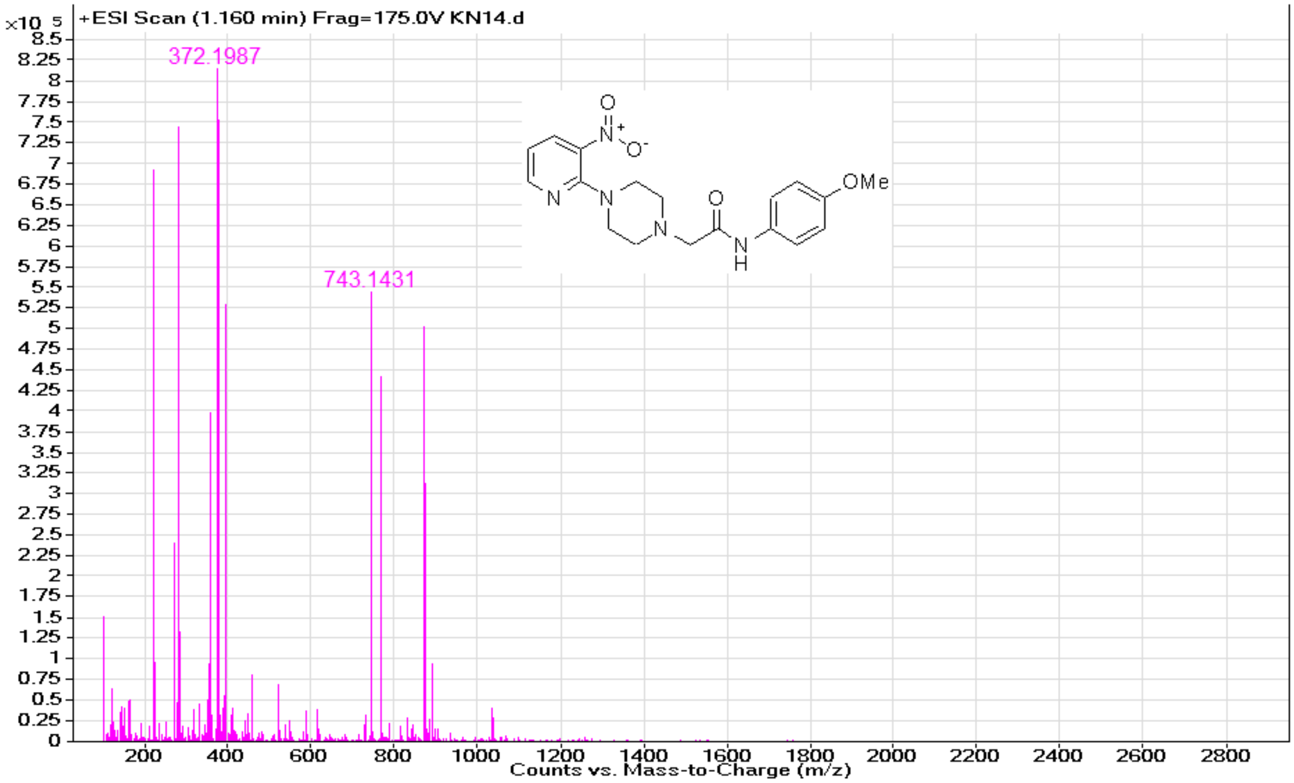

Figure S41. MS of *N-(4-Methoxyphenyl)-2-(4-(3-nitropyridin-2-yl)piperazin-1-yl)acetamide (**5o**)*

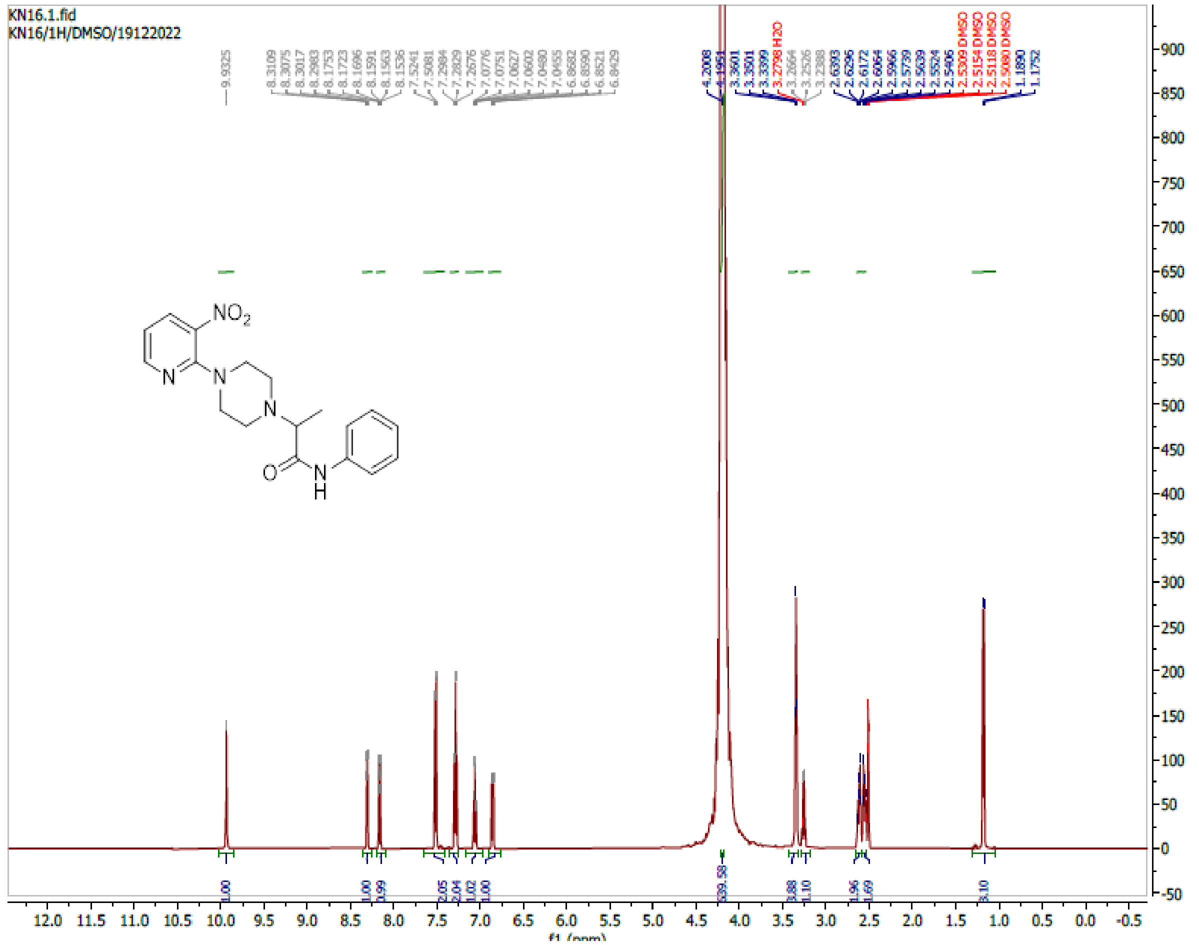

Figure S42. ^{1}HNMR of *2-(4-(3-Nitropyridin-2-yl)piperazin-1-yl)-N-phenylpropanamide* (***7a***)

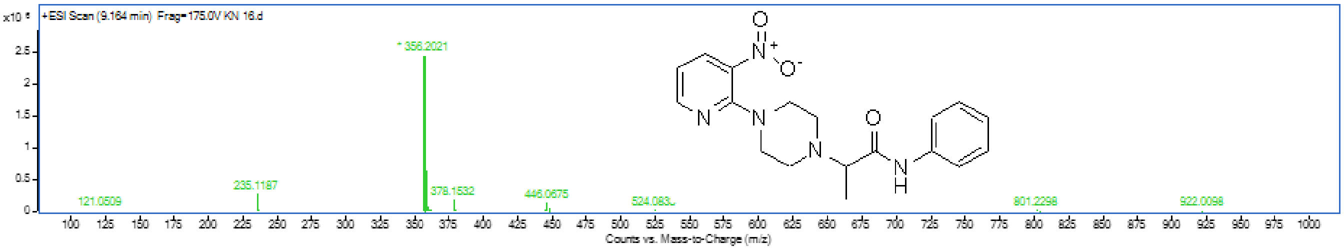

Figure S43. MS of *2-(4-(3-Nitropyridin-2-yl)piperazin-1-yl)-N-phenylpropanamide* (***7a***)

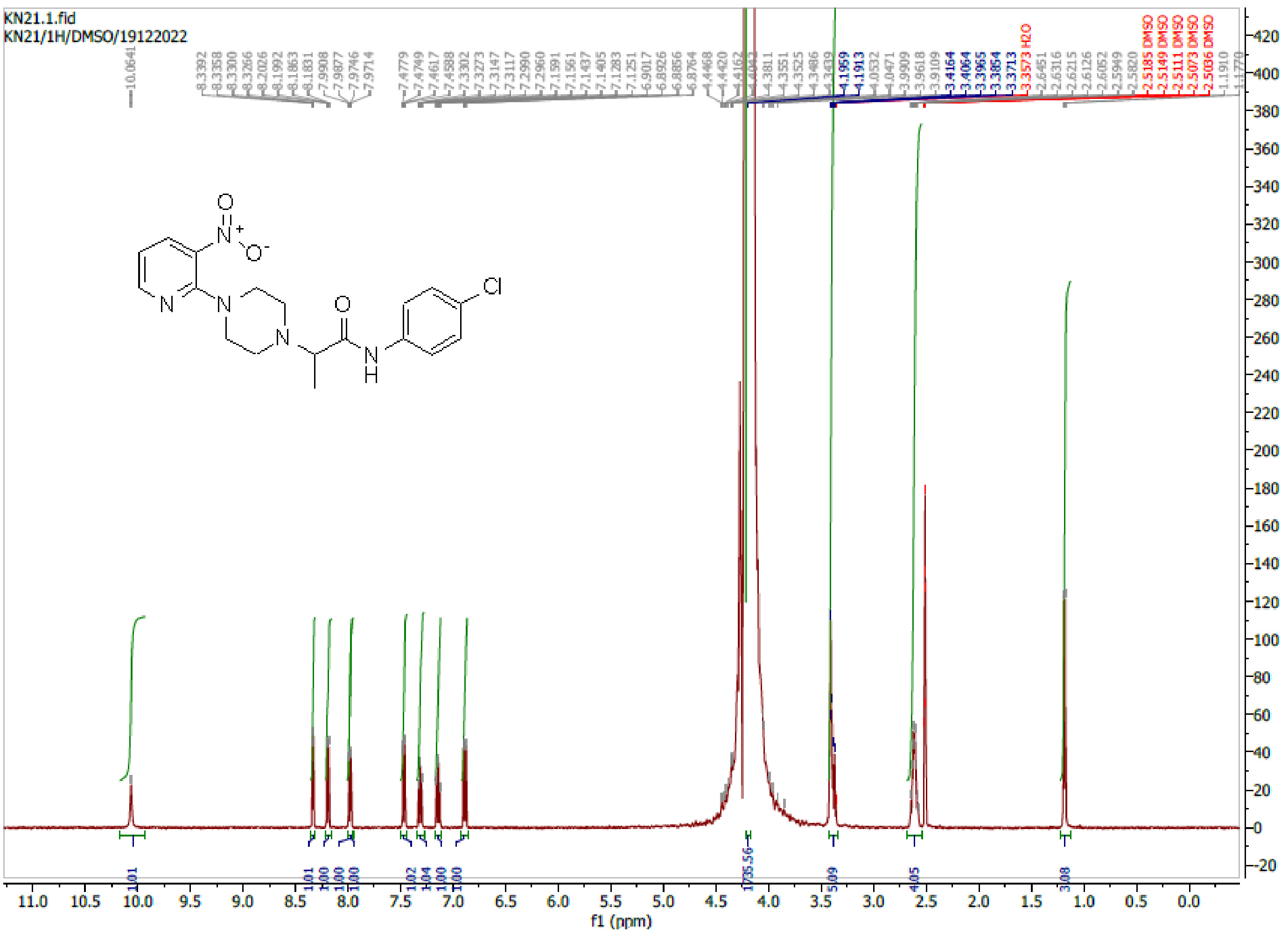

Figure S44. ^{1}HNMR of *N-(3-Chlorophenyl)-2-(4-(3-nitropyridin-2-yl)piperazin-1-yl)propanamide (**7b**)*

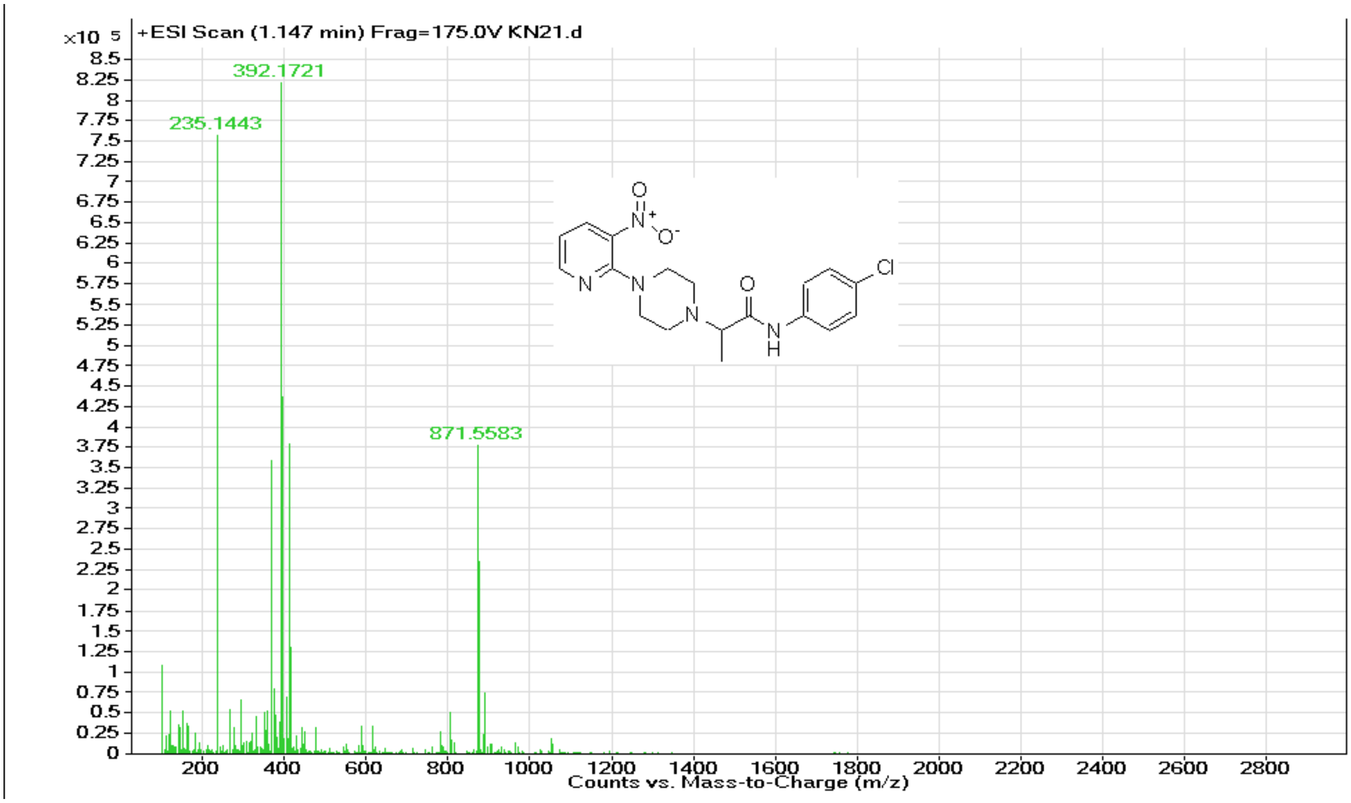

Figure S45. MS of *N-(3-Chlorophenyl)-2-(4-(3-nitropyridin-2-yl)piperazin-1-yl)propanamide* (***7b***)

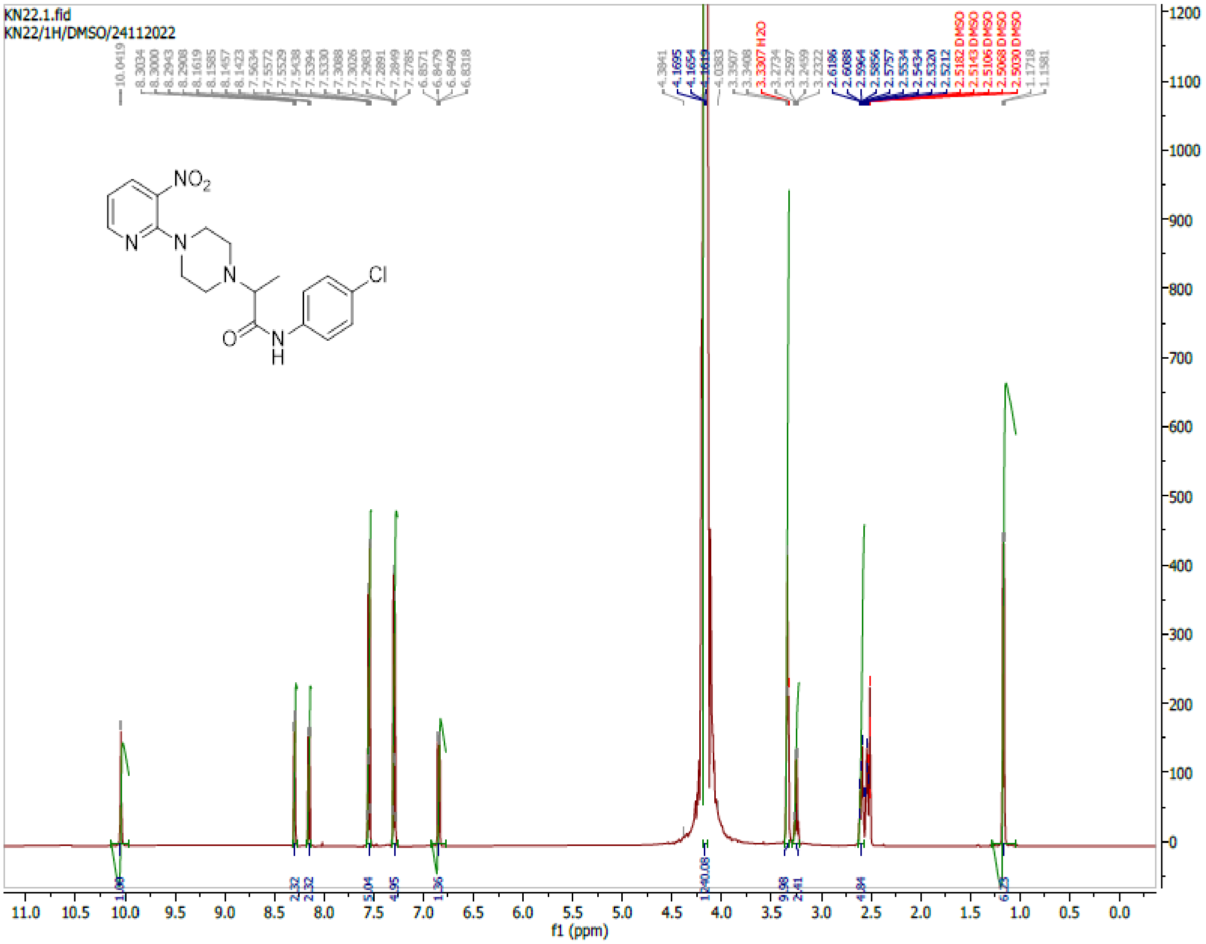

Figure S46. ^{1}HNMR of *N-(4-Chlorophenyl)-2-(4-(3-nitropyridin-2-yl)piperazin-1-yl)propanamide* (***7c***)

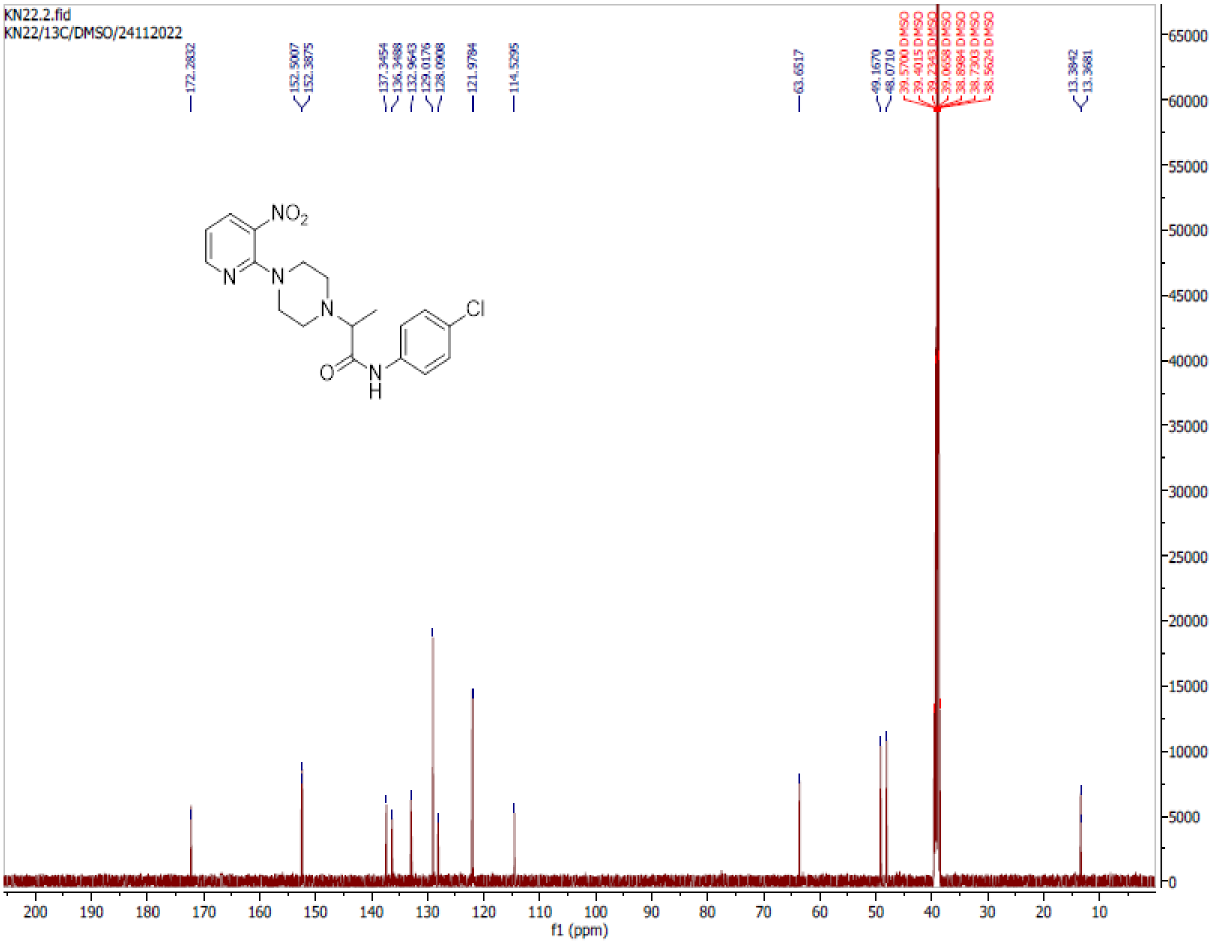

Figure S47. ^{13}CNMR of *N-(4-Chlorophenyl)-2-(4-(3-nitropyridin-2-yl)piperazin-1-yl)propanamide (**7c**)*

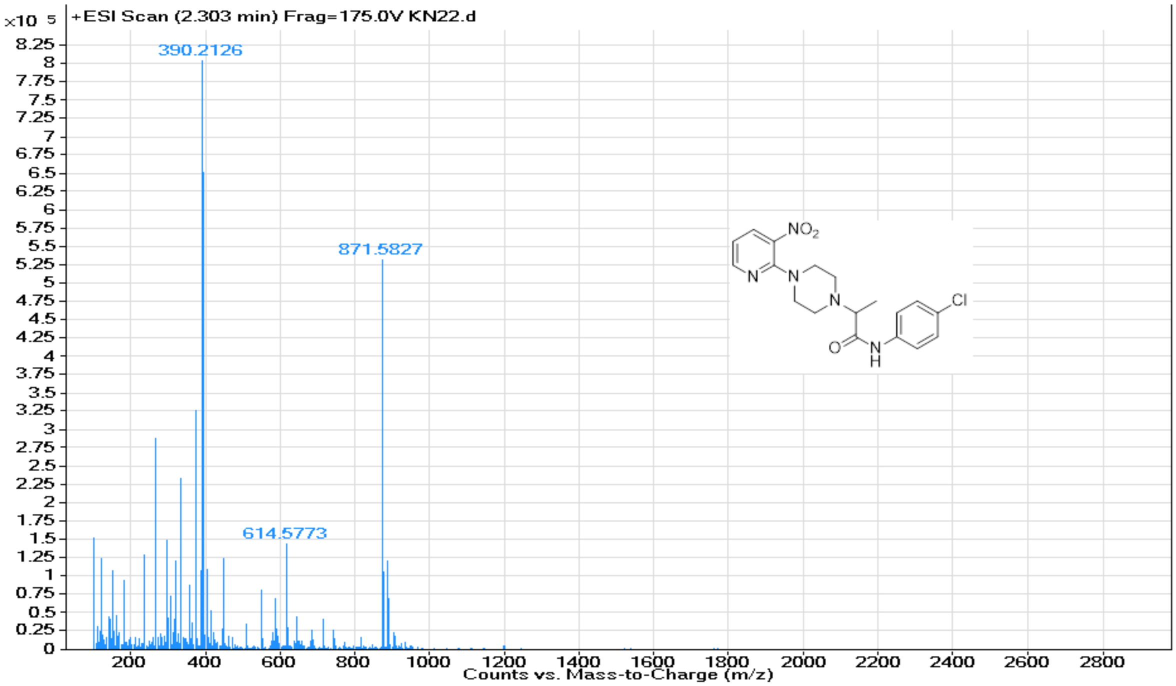

Figure S48. MS of *N-(4-Chlorophenyl)-2-(4-(3-nitropyridin-2-yl)piperazin-1-yl)propanamide* (***7c***)

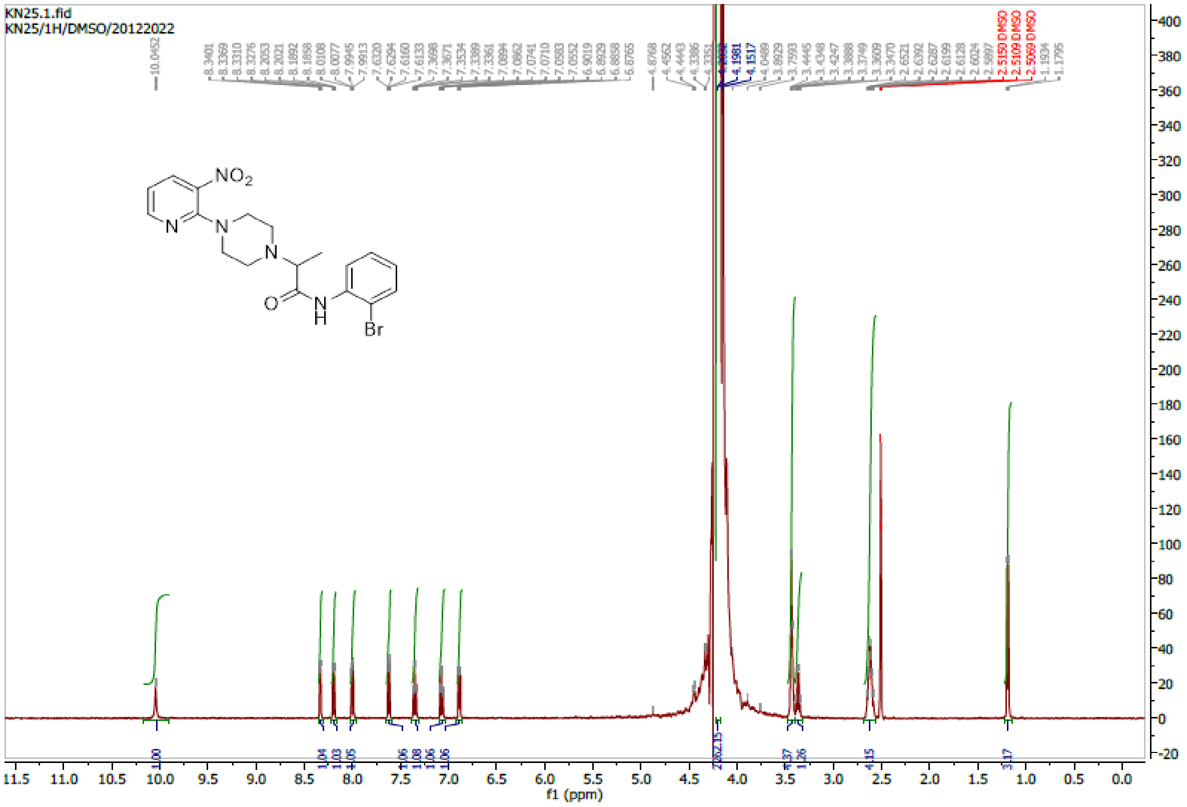

Figure S49. ^{1}HNMR of *N-(2-Bromophenyl)-2-(4-(3-nitropyridin-2-yl)piperazin-1-yl)propanamide* (***7d***)

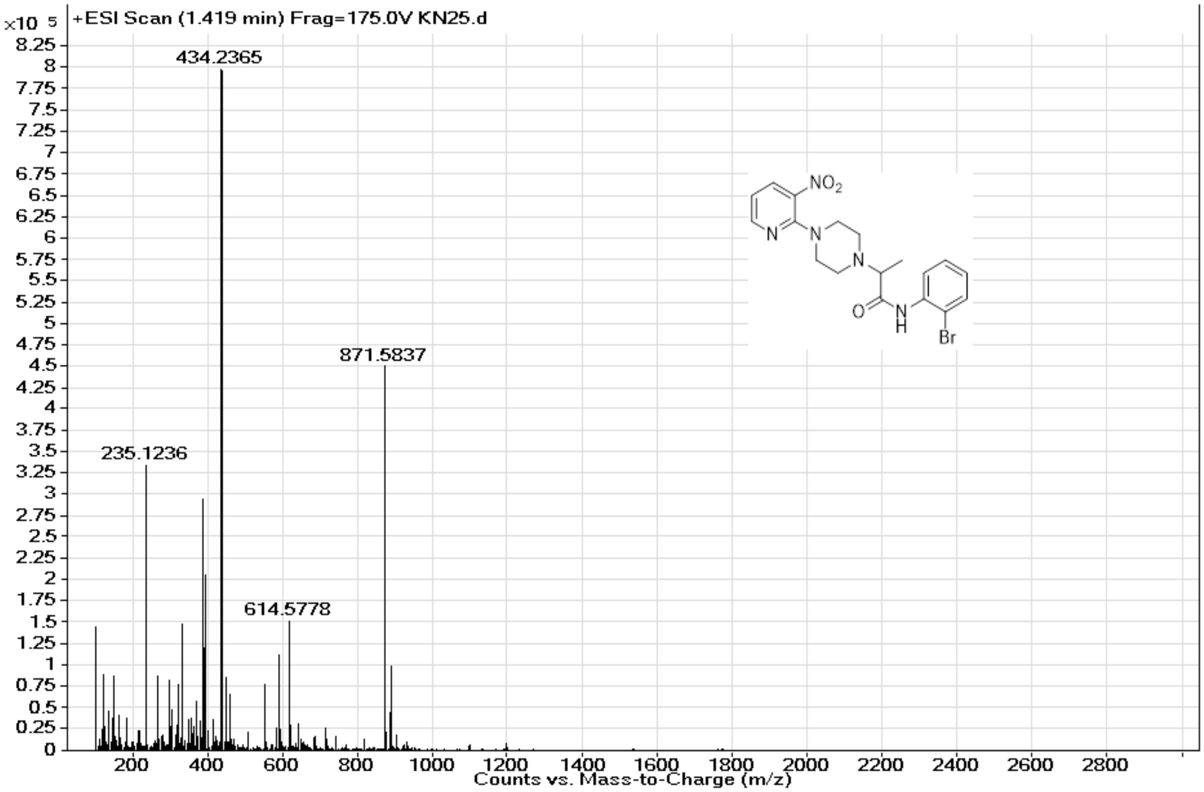

Figure S50. MS of *N-(2-Bromophenyl)-2-(4-(3-nitropyridin-2-yl)piperazin-1-yl)propanamide (**7d**)*

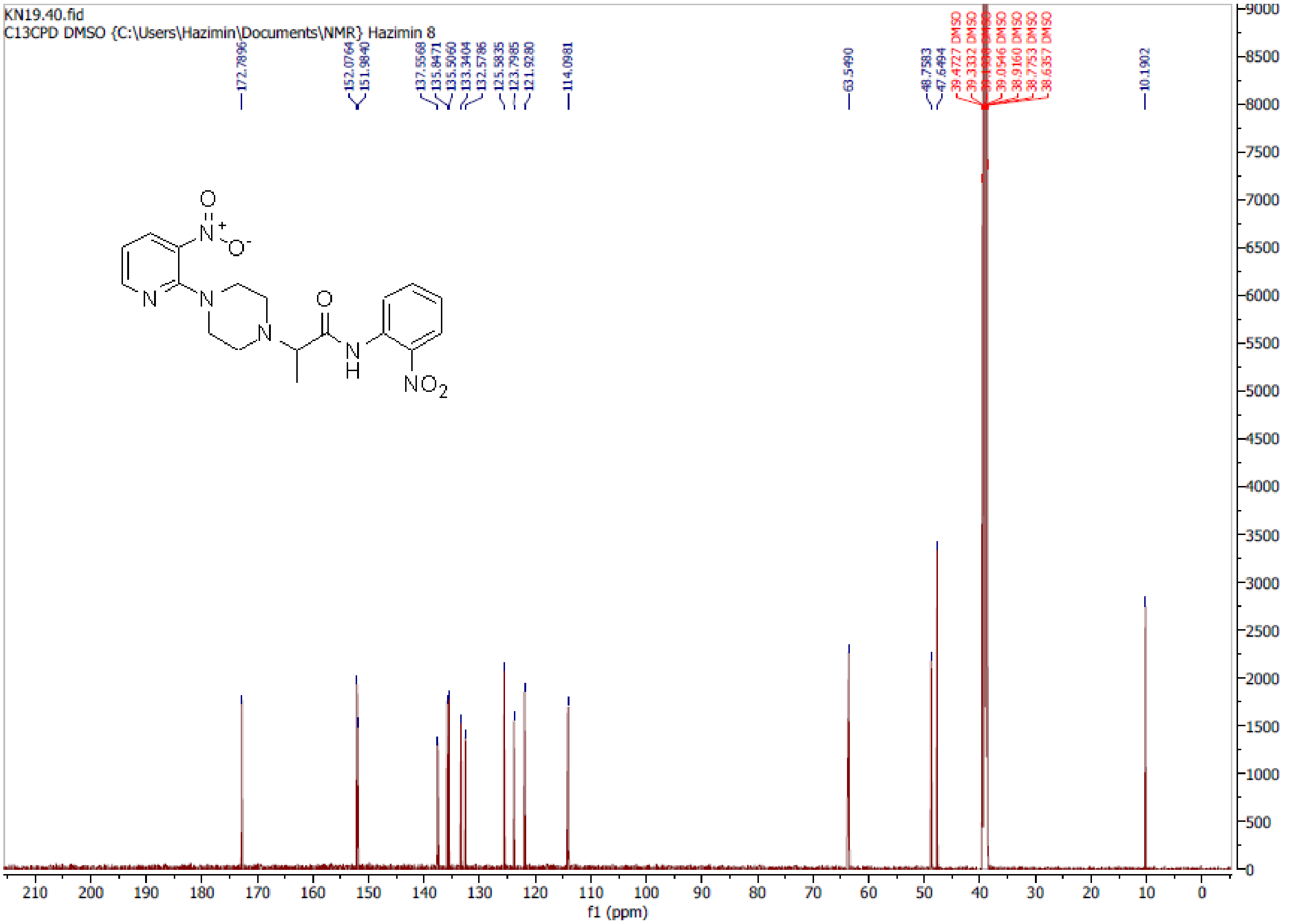

Figure S51. ¹HNMR of *N-(2-Nitrophenyl)-2-(4-(3-nitropyridin-2-yl)piperazin-1-yl)propanamide* (***7e***)

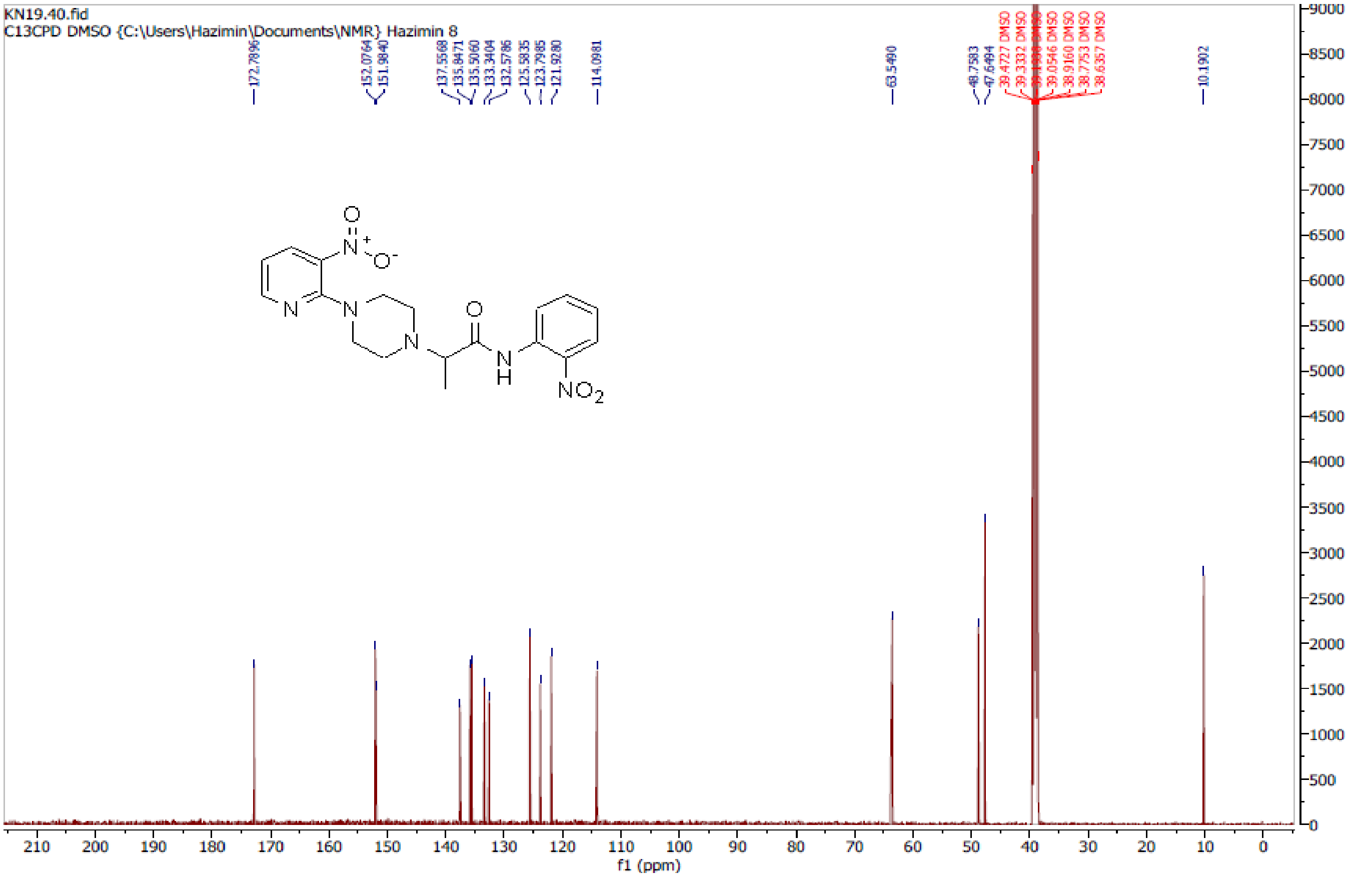

Figure S52. ^{13}CNMR of *N-(2-Nitrophenyl)-2-(4-(3-nitropyridin-2-yl)piperazin-1-yl)propanamide* (***7e***)

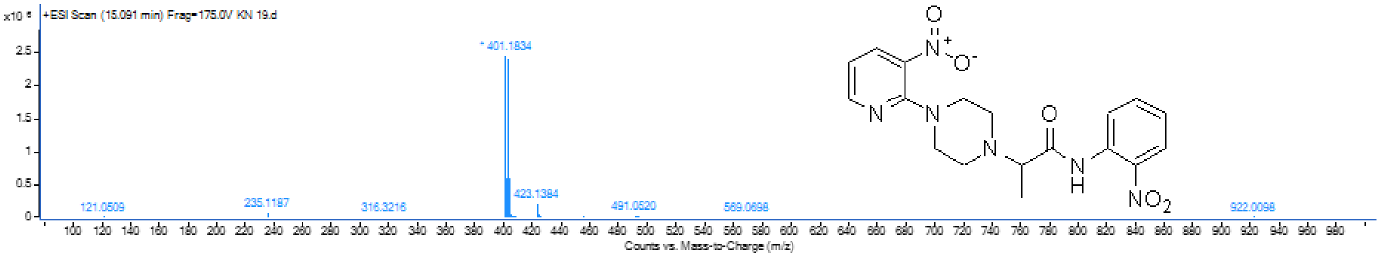

Figure S53. MS of *N-(2-Nitrophenyl)-2-(4-(3-nitropyridin-2-yl)piperazin-1-yl)propanamide* (***7e***)

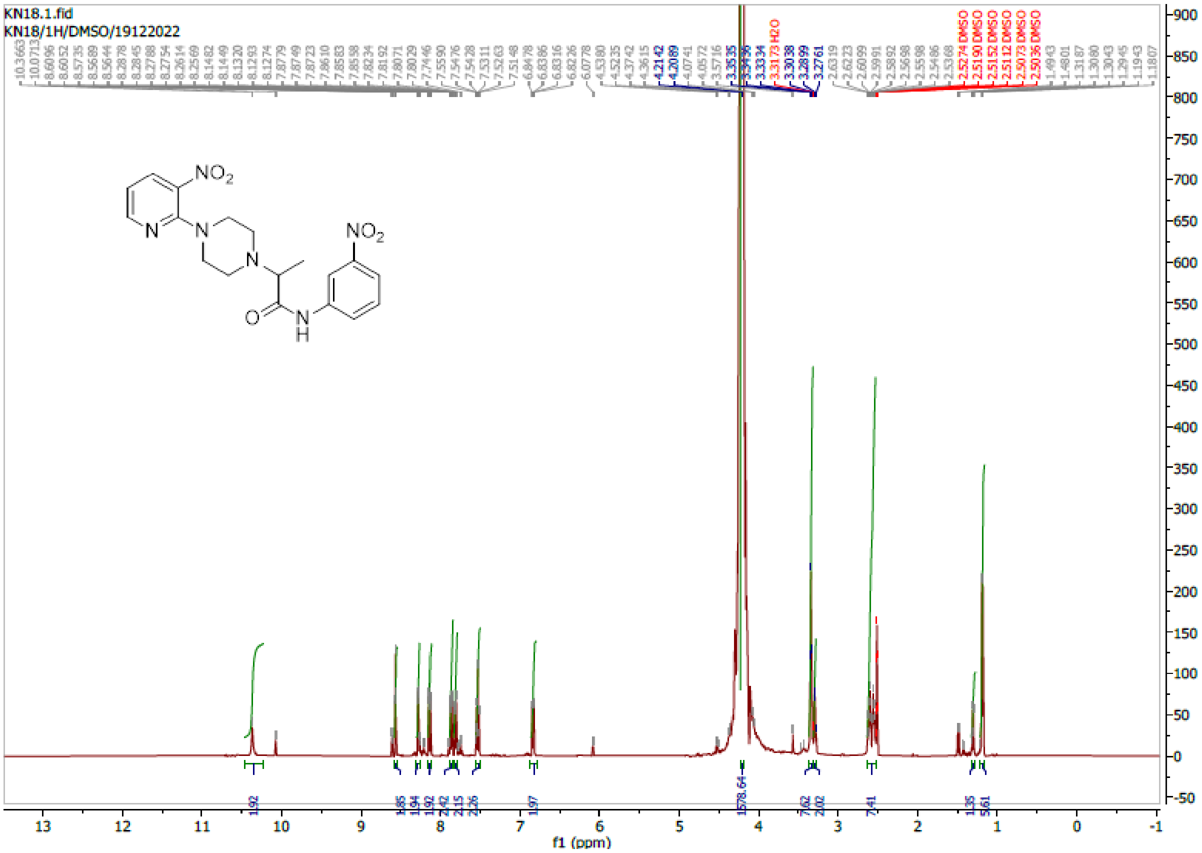

Figure S54. ^{1}HNMR of *N-(3-Nitrophenyl)-2-(4-(3-nitropyridin-2-yl)piperazin-1-yl)propanamide* (**7f**)

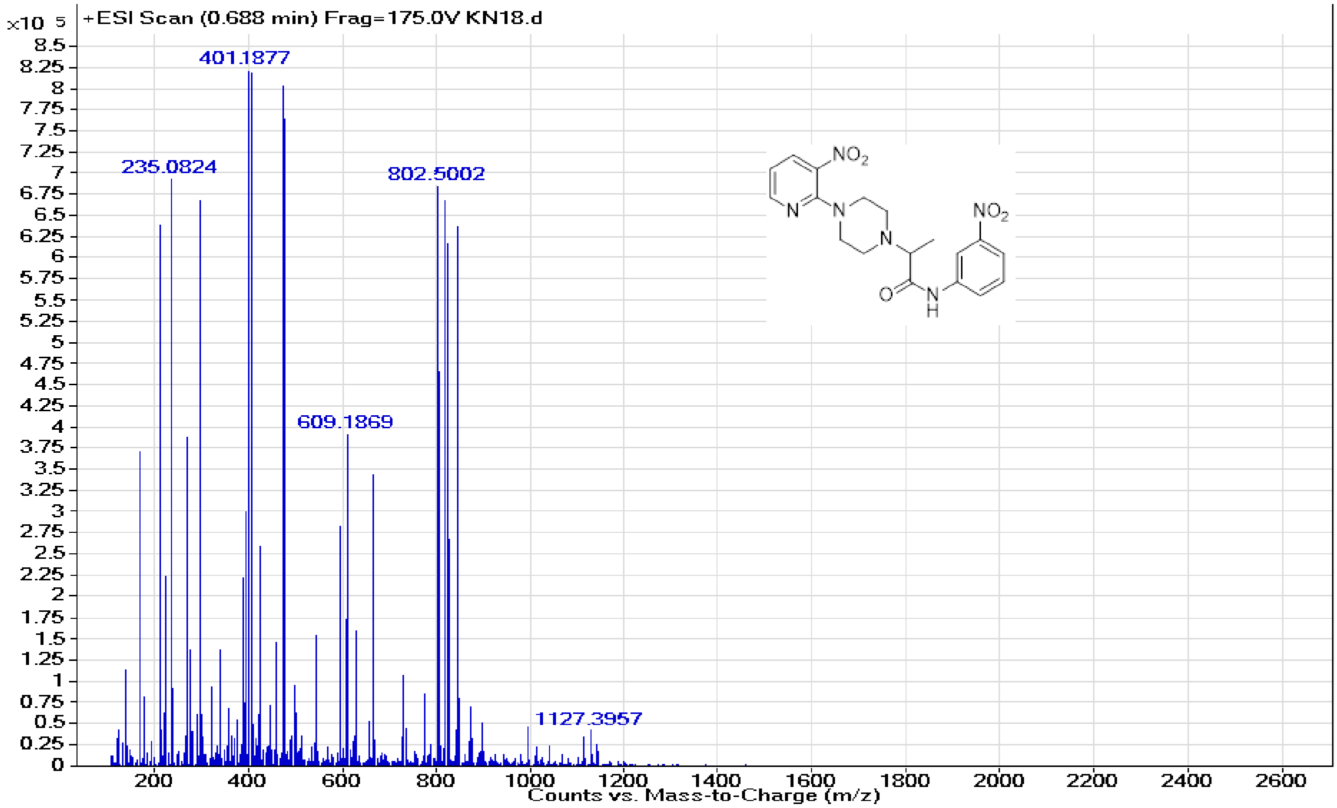

Figure S55. MS of *N-(3-Nitrophenyl)-2-(4-(3-nitropyridin-2-yl)piperazin-1-yl)propanamide* (**7f**)

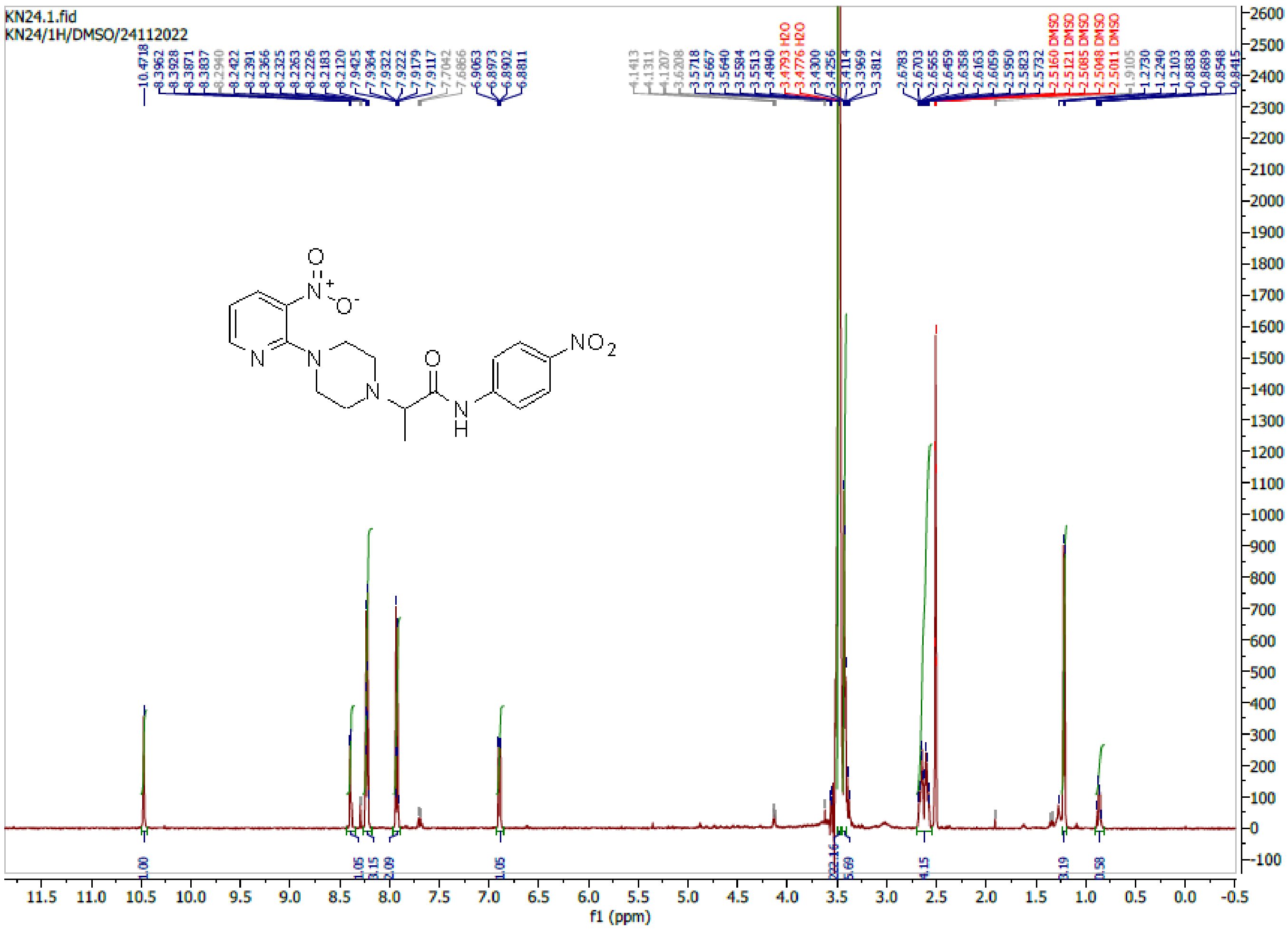

Figure S56. ^{1}HNMR of *N-(4-Nitrophenyl)-2-(4-(3-nitropyridin-2-yl)piperazin-1-yl)propanamide (**7g**)*

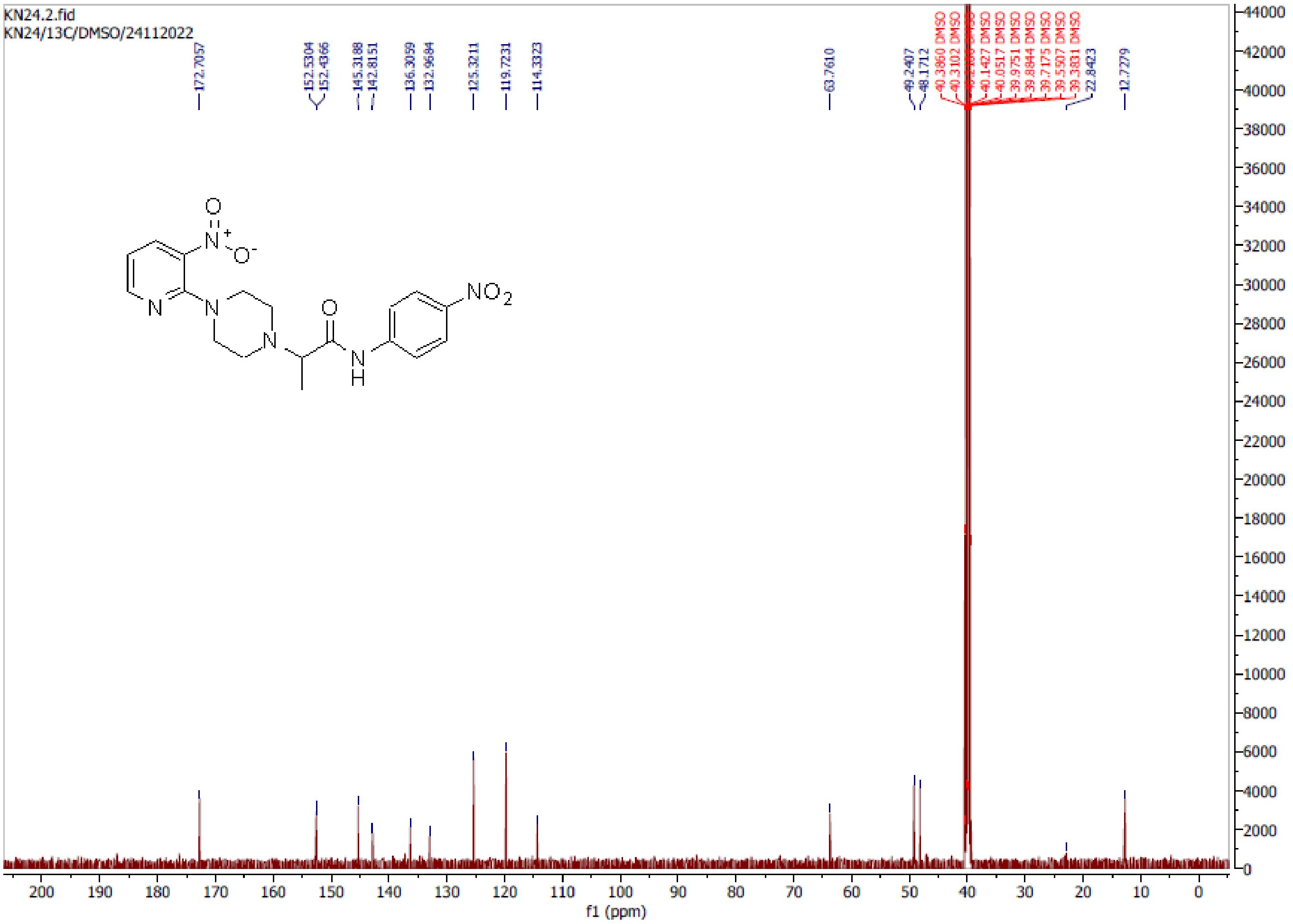

Figure S57. 13CNMR of *N-(4-Nitrophenyl)-2-(4-(3-nitropyridin-2-yl)piperazin-1-yl)propanamide (**7g**)*

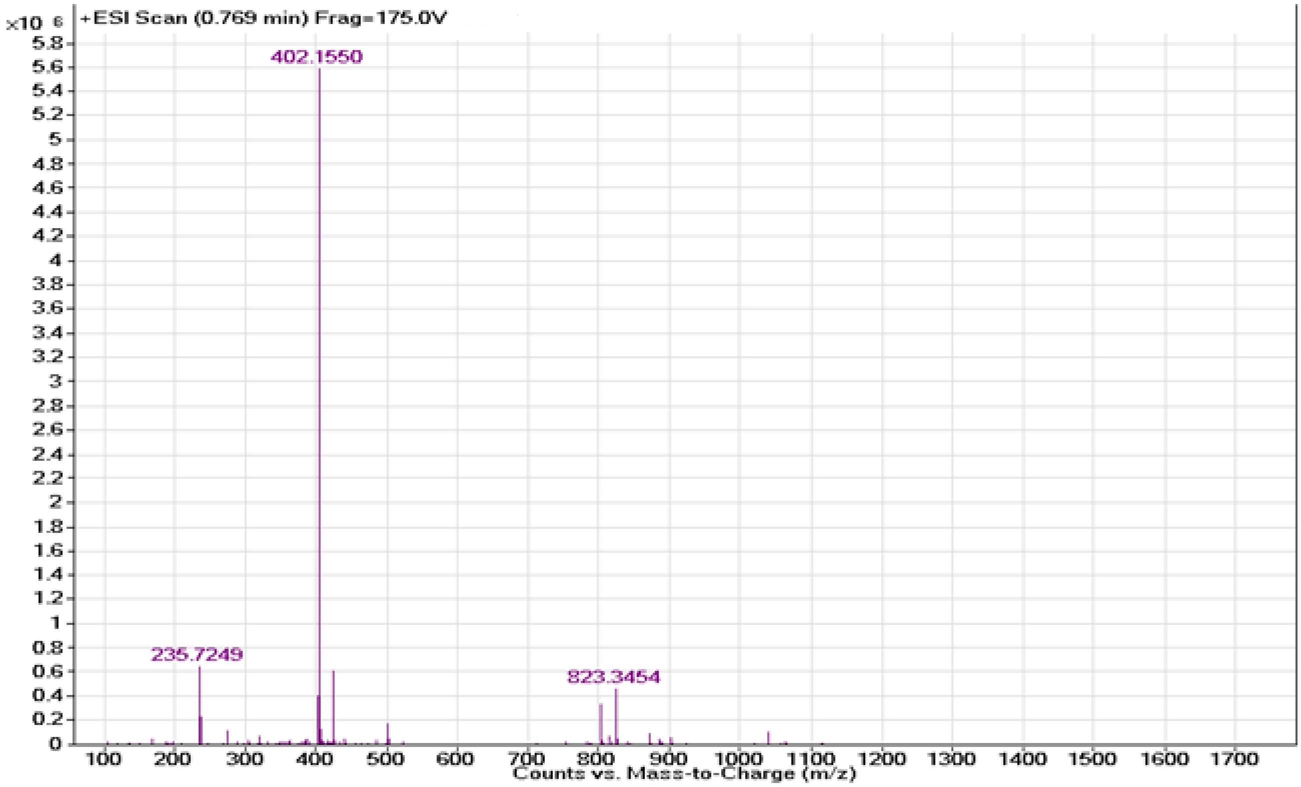

Figure S58. MS of *N-(4-Nitrophenyl)-2-(4-(3-nitropyridin-2-yl)piperazin-1-yl)propanamide (**7g**)*

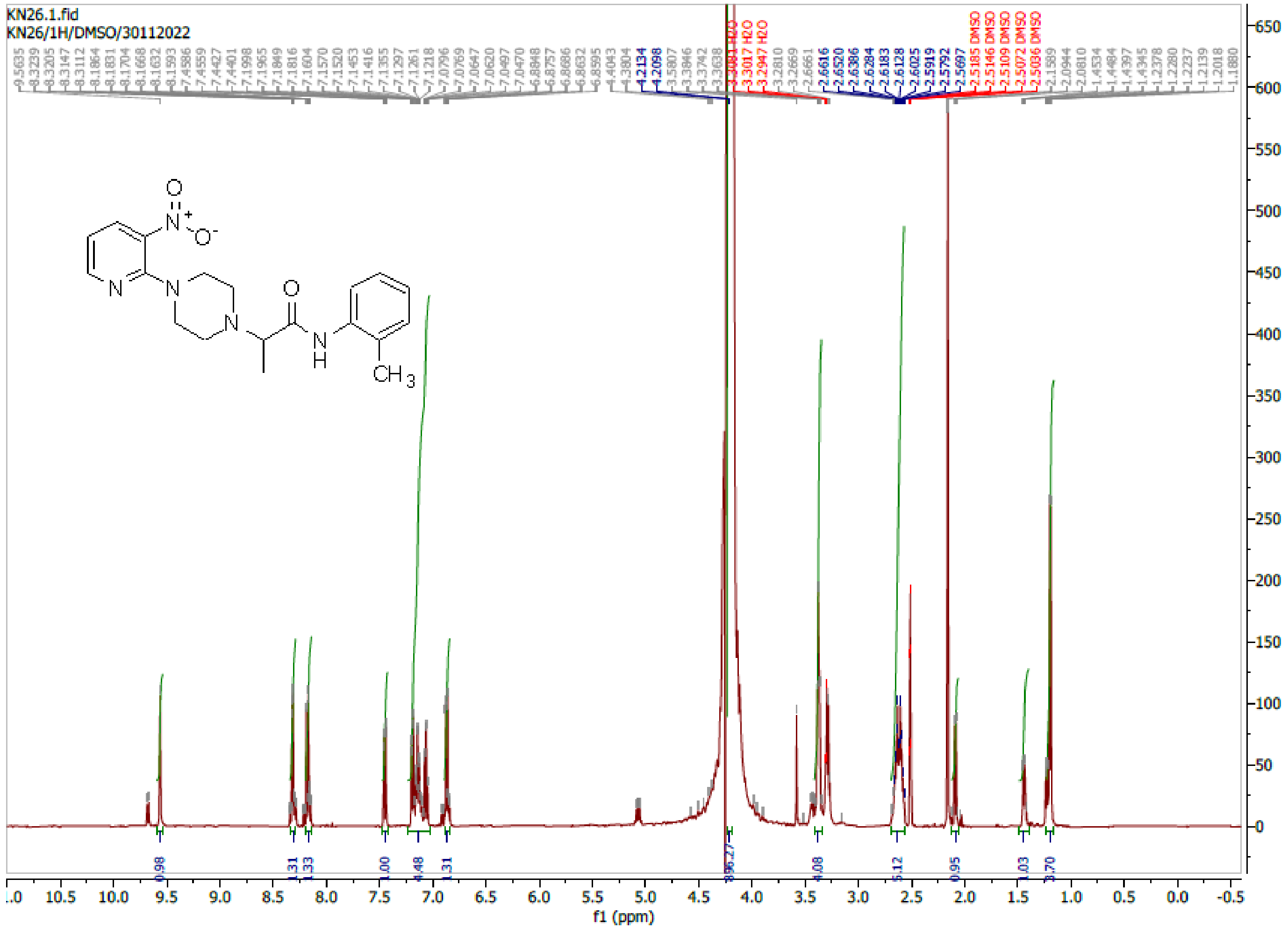

Figure S59. ^{1}HNMR of *N-(o-Tolyl)-2-(4-(3-nitropyridin-2-yl)piperazin-1-yl)propanamide (**7h**)*

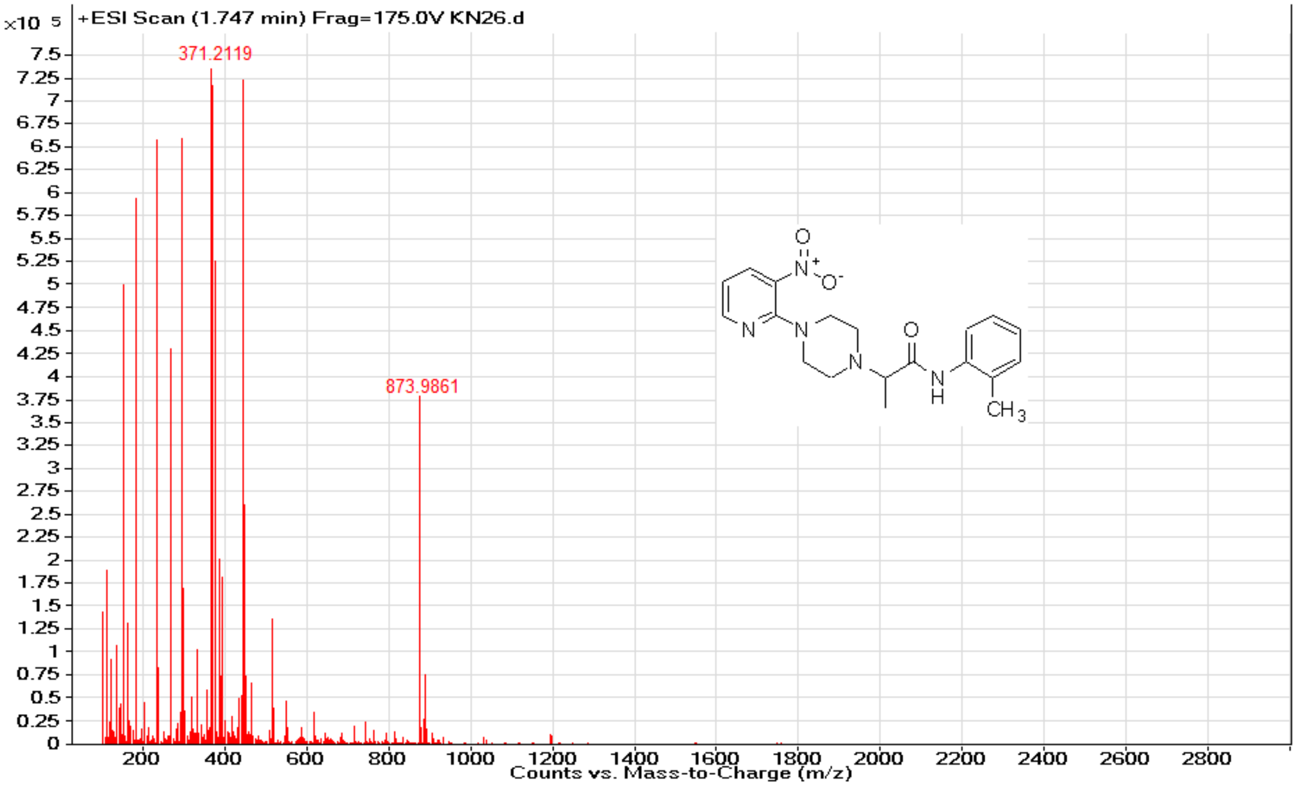

Figure S60. MS of *N-(o-Tolyl)-2-(4-(3-nitropyridin-2-yl)piperazin-1-yl)propanamide* (***7h***)

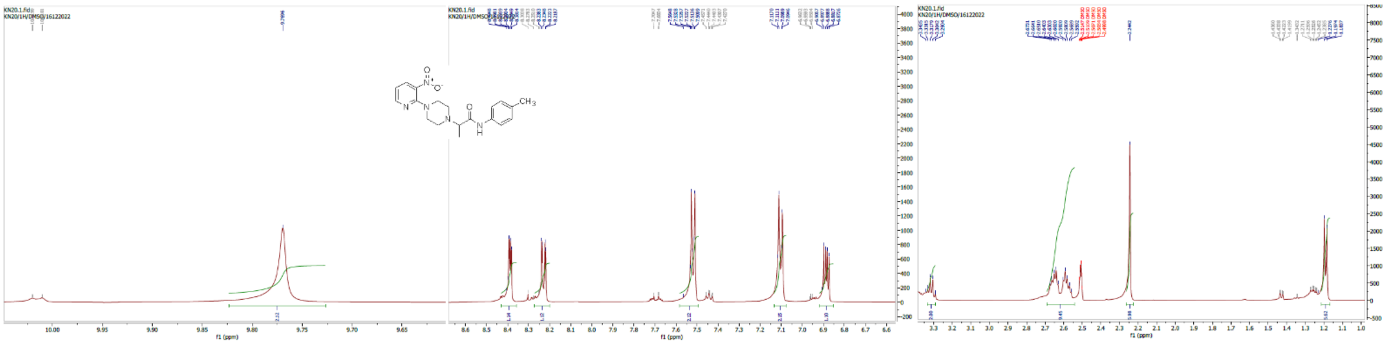

Figure S61. ^{1}HNMR of *N-(p-Tolyl)-2-(4-(3-nitropyridin-2-yl)piperazin-1-yl)propanamide* (***7i***)

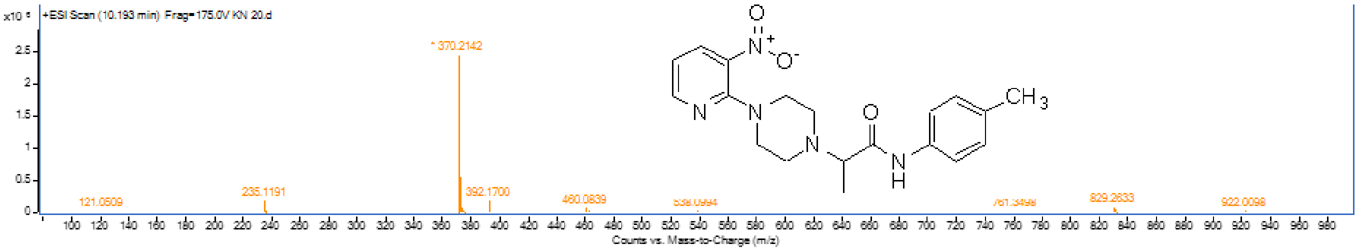

Figure S62. MS of *N-(p-Tolyl)-2-(4-(3-nitropyridin-2-yl)piperazin-1-yl)propanamide* (***7i***)

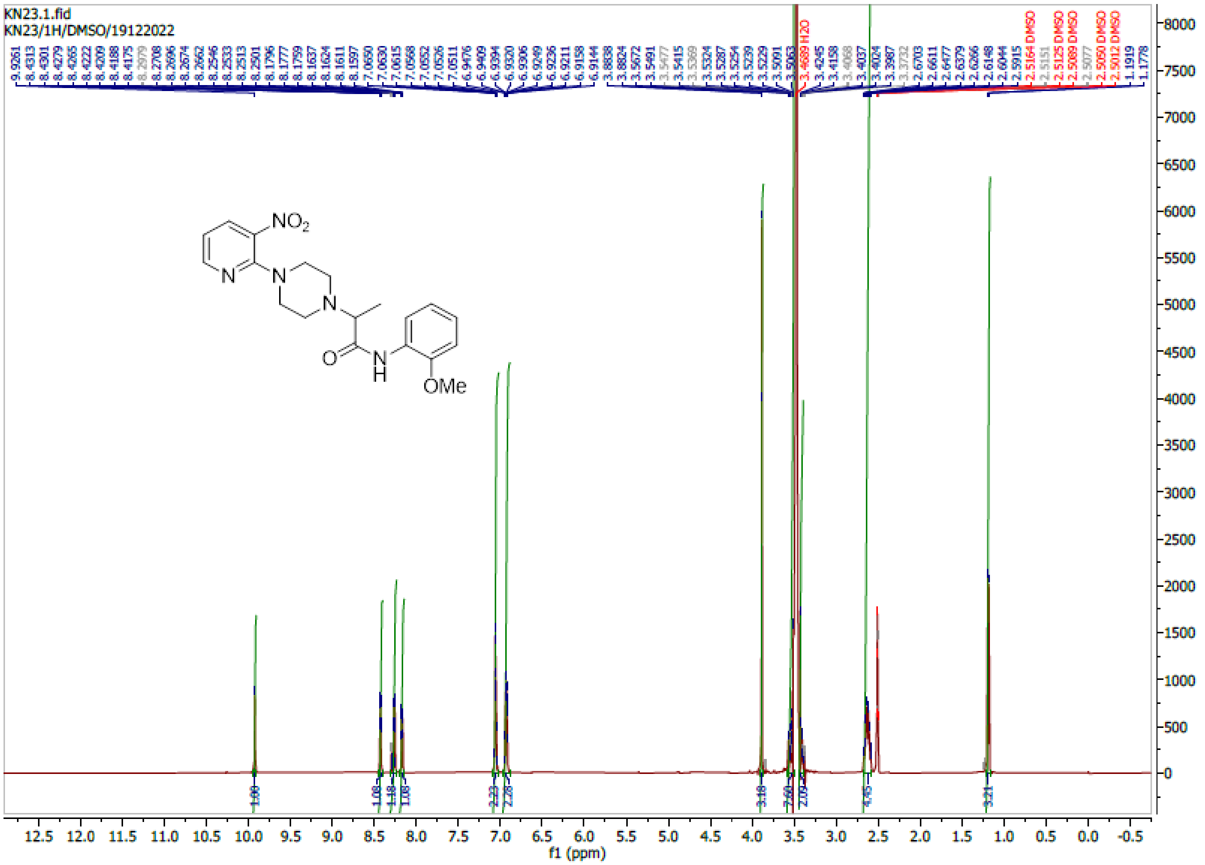

Figure S63. ^{1}HNMR of *N-(2-Methoxyphenyl)-2-(4-(3-nitropyridin-2-yl)piperazin-1-yl)propanamide* (***7j***)

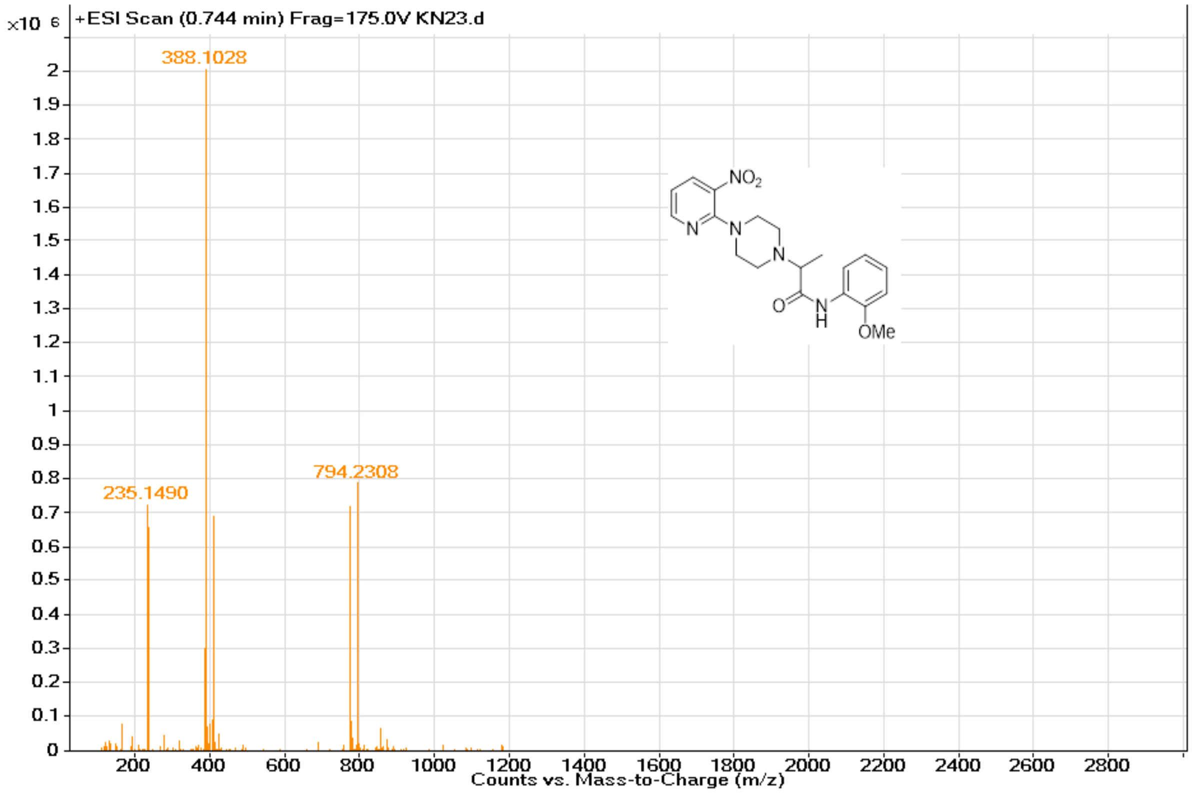

Figure S64. MS of *N-(2-Methoxyphenyl)-2-(4-(3-nitropyridin-2-yl)piperazin-1-yl)propanamide* (***7j***)

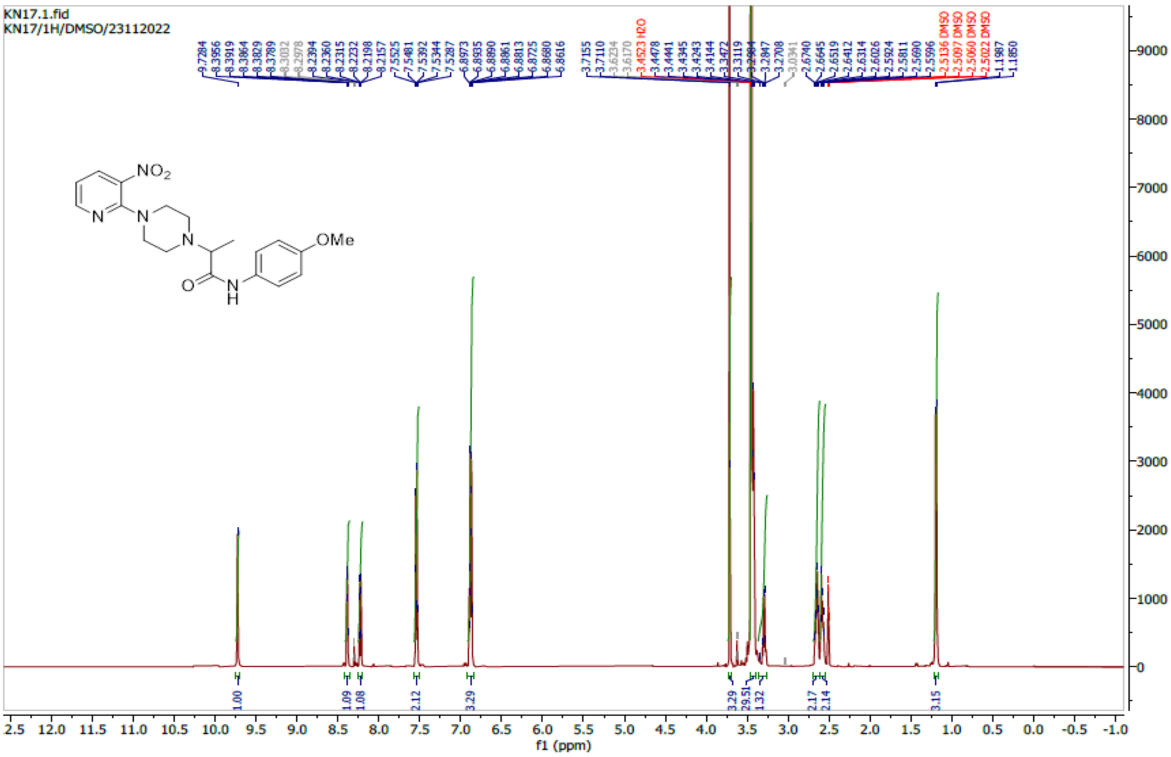

Figure S65. ¹HNMR of *N-(4-Methoxyphenyl)-2-(4-(3-nitropyridin-2-yl)piperazin-1-yl)propanamide* (***7k***)

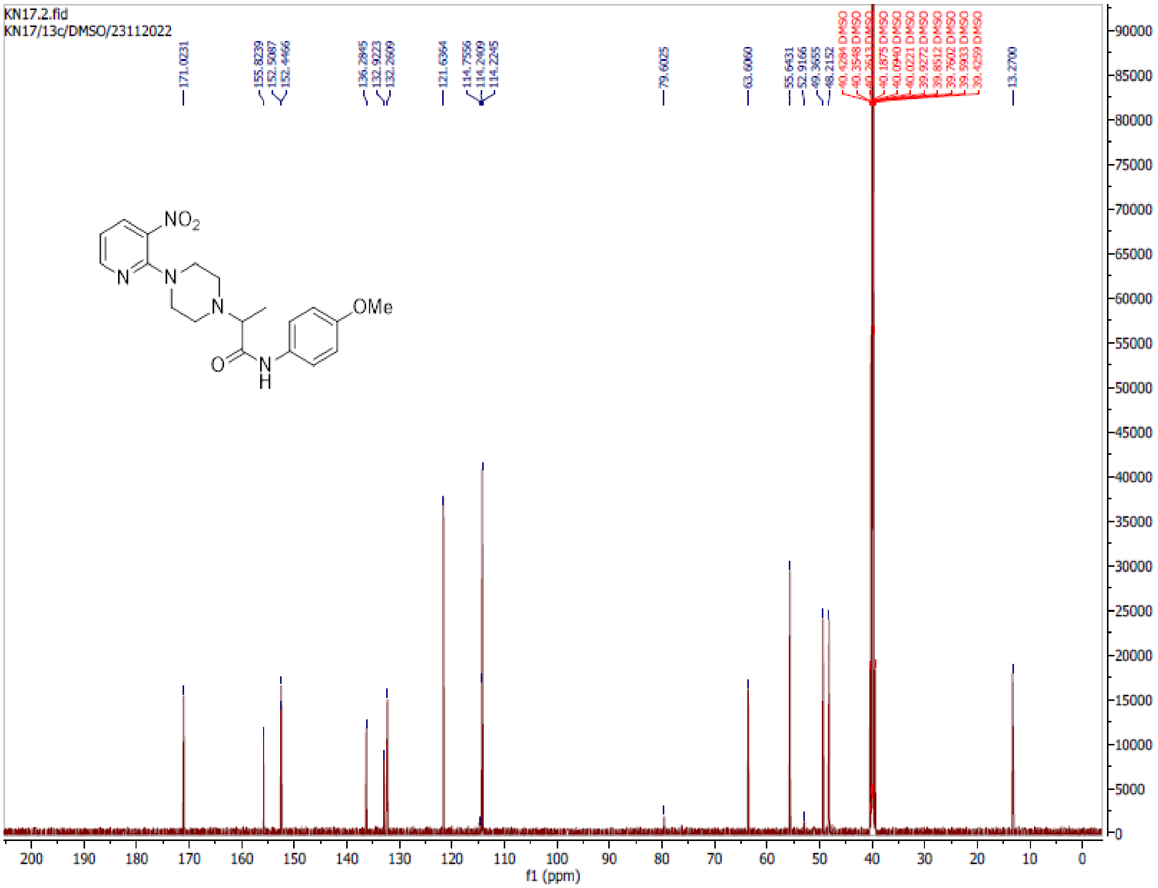

Figure S66. ^{13}CNMR of *N-(4-Methoxyphenyl)-2-(4-(3-nitropyridin-2-yl)piperazin-1-yl)propanamide* (***7k***)

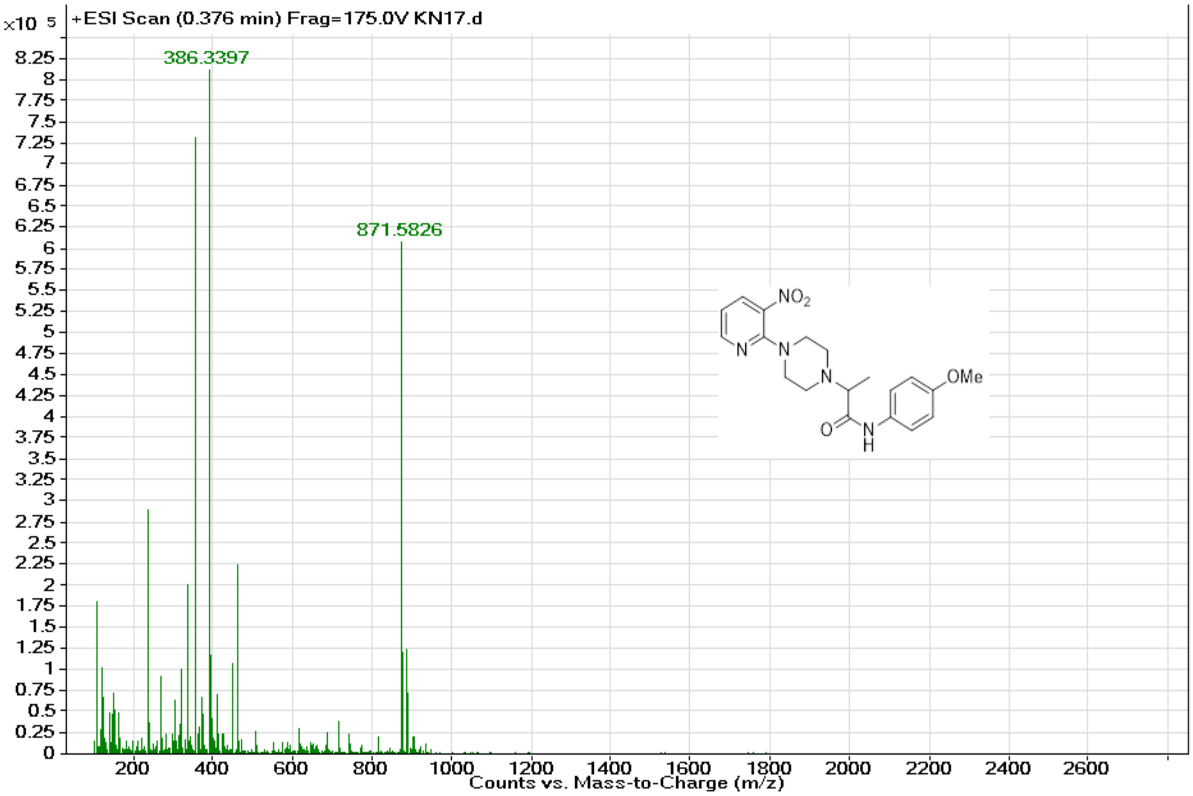

Figure S68. MS of *N-(4-Methoxyphenyl)-2-(4-(3-nitropyridin-2-yl)piperazin-1-yl)propanamide* (***7k***)

Table S1. *Prediction of toxic substructures of the most potent inhibitors (**5b** and **7e**), where the red highlighted atoms are the substructures.*

Nature of toxic substructure	Structure
**5b**	
Alert from Top200 Drug	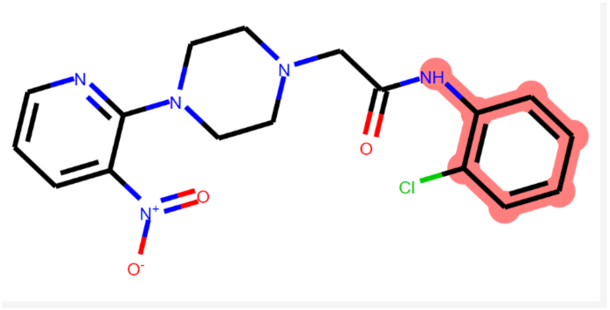

Potential electrophilic agents	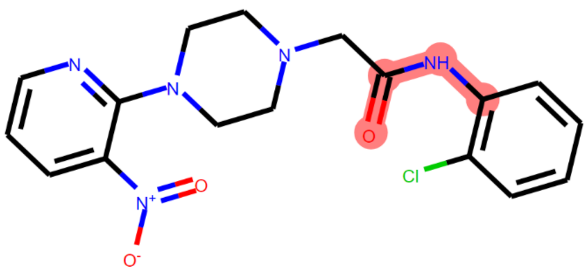
Idiosyncratic toxicity	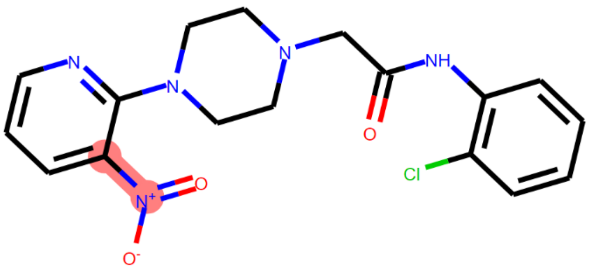
Covalent bind with protein	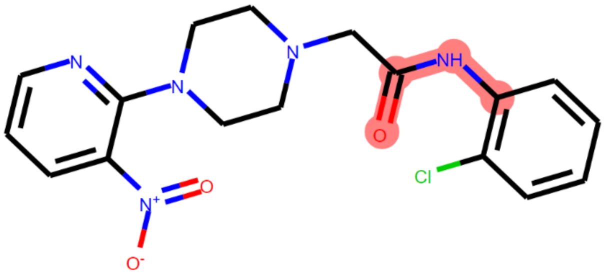
Covalent bind with DNA	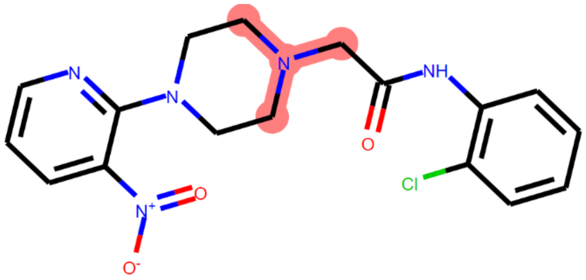
Covalent bind with DNA	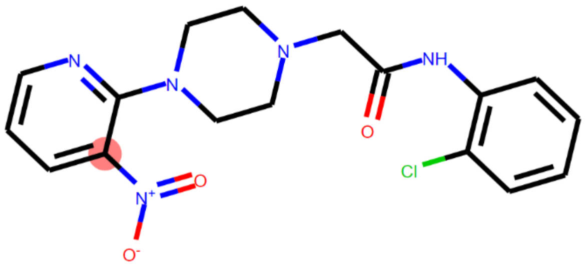

Covalent bind with DNA	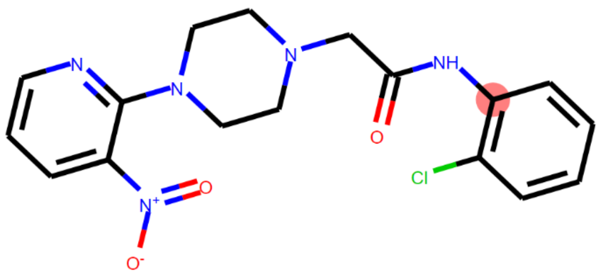
Non-genotoxic carcinogenicity	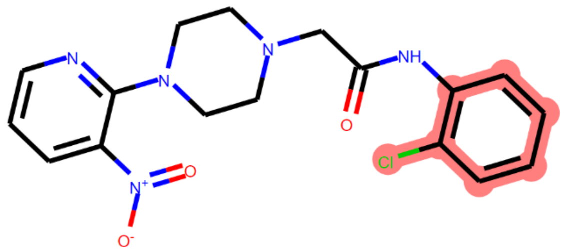
Skin sensitization	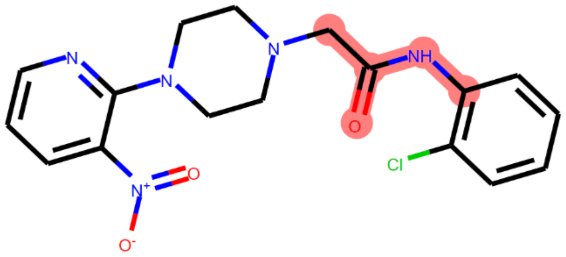
**7e**	
Alter from Top200 Drug	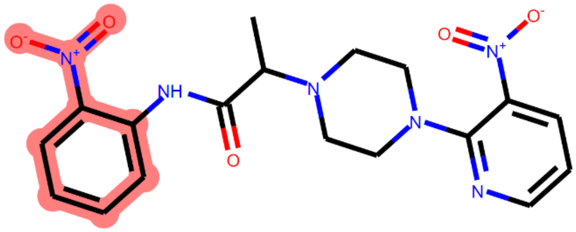
Alter from Top200 Drug	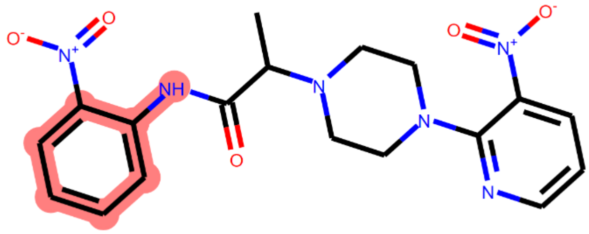

Potential electrophilic agent	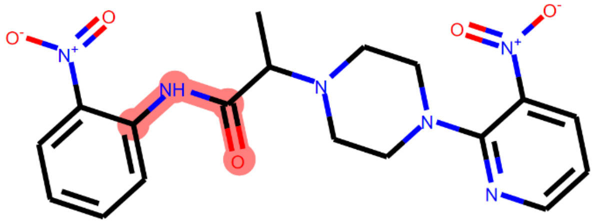
Idiosyncratic toxicity	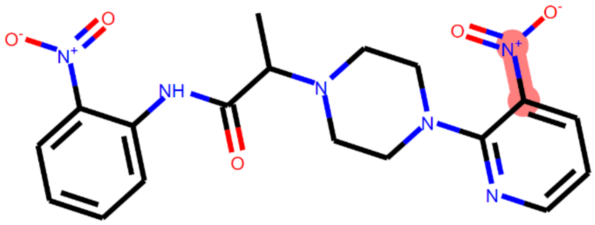
Covalent bind with protein	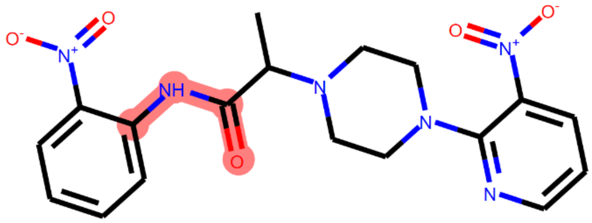
Covalent bind with DNA	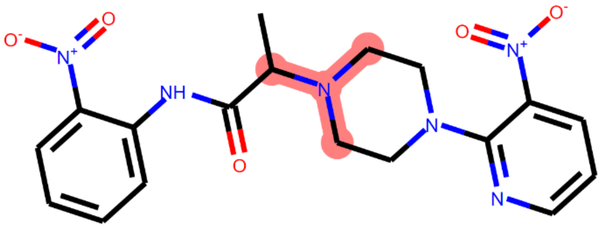
Covalent bind with DNA	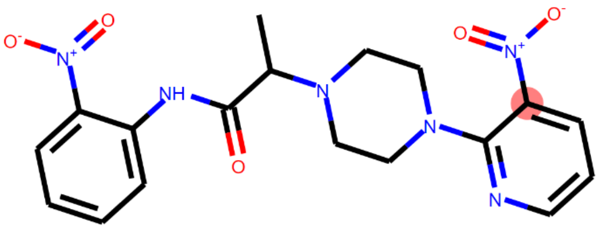
Covalent bind with DNA	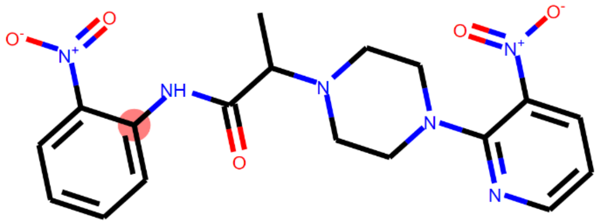

Skin sensitization	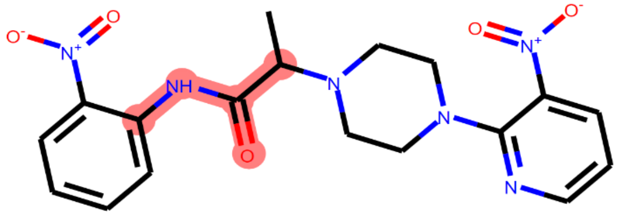

Table S2. GUSAR analysis was employed to identify a less toxic route of administration for the most potent inhibitors (**5b** and **7e**) in rats.

**Compounds**	**Administration route**	**LD_{50} log10 (mmol/kg)**	**LD_{50} (mg/kg)**	**Predicted toxicity class**
**5b**	Intraperitoneal (IP)	0.002	377.7	4
	Intravenous (IV)	-0.719	71.7	4
	Oral administration	0.065	436.3	4
	Subcutaneous (SC)	0.558	1360	5
**7e**	Intraperitoneal (IP)	0.107	511.8	5
	Intravenous (IV)	-0.942	45.780	4
	Oral administration	0.357	911.1	4
	Subcutaneous (SC)	-0.054	353.6	4

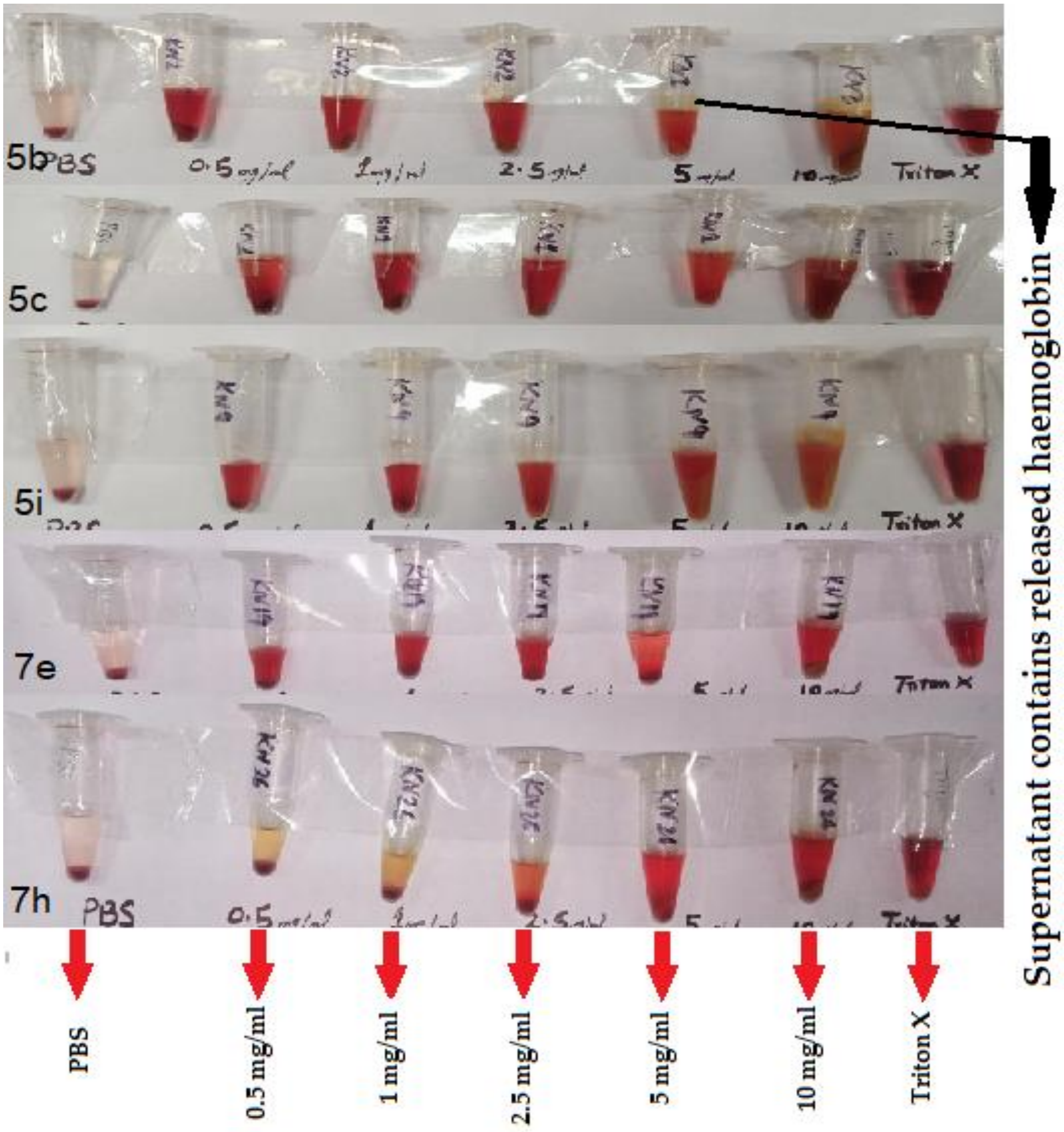

Figure S69: Hemolysis test was employed to identify effects of 1-(3-nitropyridin-2-yl)piperazine derivatives (**5b, 5c, 5i; 7e, 7h**) on human blood.
